# Supplementary figures and images for: Carious lesions in permanent dentitions are reduced in remote Indigenous Australian children taking part in a non-randomised preventive trial
Source: PLoS One. 2021 Jan 28;16(1):e0244927. doi: 10.1371/journal.pone.0244927 (PMC7842954; doi:10.1371/journal.pone.0244927)

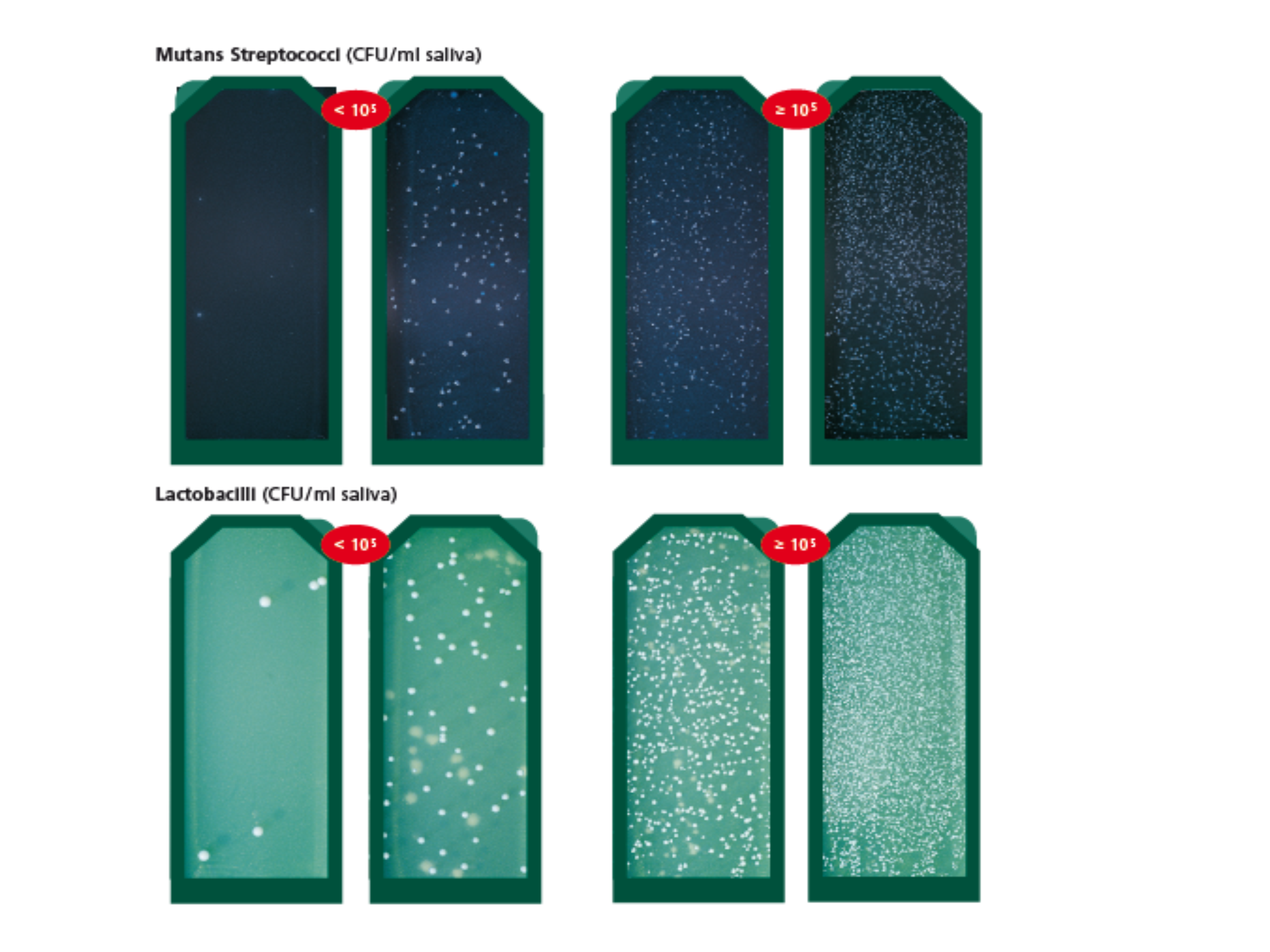

Supplement: S1 Fig — (TIF) [file pone.0244927.s002.tif]
