# Supplementary material for: Carious lesions in permanent dentitions are reduced in remote Indigenous Australian children taking part in a non-randomised preventive trial
Source: PLoS One. 2021 Jan 28;16(1):e0244927. doi: 10.1371/journal.pone.0244927 (PMC7842954; doi:10.1371/journal.pone.0244927)
Supplement: S1 Table — (DOCX) [file pone.0244927.s003.docx]

**Supplementary table 1:** ITT analysis of caries increment at two-year follow-up between Intervention and Comparison groups

|  | | Deciduous tooth surfaces | | Permanent tooth surfaces | |
| --- | --- | --- | --- | --- | --- |
|  |  | Mean (SE) | IRR (95% CI) | Mean (SE) | IRR (95% CI) |
| *Incipient caries increment* | |  |  |  |  |
| Comparison | | 2.83 (0.48) | 1.32 (0.99-1.75) | 6.10 (0.51) | 1.12 (0.88-1.41) |
| Intervention | | 2.28 (2.34) | Ref | 5.17 (0.37) | Ref |
| p-value^a^ |  |  | 0.17 |  | 0.44 |
| Preventive fraction^b^ |  | 19% |  | 15% |  |
| *Advanced caries increment* | |  |  |  |  |
| Comparison | | 2.80 (0.66) | 1.07 (0.81-1.42) | 1.40 (0.11) | 1.61 (1.20-2.17) |
| Intervention | | 2.44 (0.28) | Ref | 0.82 (0.12) | Ref |
| p-value^a^ |  |  | 0.90 |  | **0.03** |
| Preventive fraction^b^ |  | 13% |  | 41% |  |
| *Total caries increment* | |  |  |  |  |
| Comparison | | 5.62 (1.06) | 1.17 (0.90-1.51) | 7.5 (0.51) | 1.18 (0.93-1.48) |
| Intervention | | 4.72 (0.45) | Ref | 5.99 (0.43) | Ref |
| p-value^a^ |  |  | 0.59 |  | 0.23 |
| Preventive fraction^b^ |  | 6% |  | 20% |  |

*aNegative binomial with log link regression adjusted for baseline caries experience, P<0.05 in bold font ^b^Preventive fraction = mean increment in Comparison – mean increment in Intervention ÷ mean increment in Comparison X 100*
