# Supplementary material for: Carious lesions in permanent dentitions are reduced in remote Indigenous Australian children taking part in a non-randomised preventive trial
Source: PLoS One. 2021 Jan 28;16(1):e0244927. doi: 10.1371/journal.pone.0244927 (PMC7842954; doi:10.1371/journal.pone.0244927)
Supplement: S2 Table — (DOCX) [file pone.0244927.s004.docx]

**Supplementary table 2:** Multivariable analyses with increment in advanced caries at two-years follow-up as the dependent variable (ITT) – adjusted analysis

| Explanatory variables | IRR (95% CI) | P |
| --- | --- | --- |
| Group allocation |  |  |
| Comparison | 1.62 (1.18-2.23) | **0.026** |
| Intervention | **Ref** |  |
| Sex |  |  |
| Males | 1.22 (0.90-1.66) | 0.272 |
| Females | **Ref** |  |
| Age | 1.05 (0.99-1.11) | 0.187 |
| Baseline caries experience | 1.04 (0.99-1.09) | 0.148 |
| Brushing group |  |  |
| Once or less | 1.04 (0.74-1.48) | 0.912 |
| Twice or more | **Ref** |  |
| Soft drinks consumption on a typical day |  |  |
| Yes | 1.56 (0.91-2.69) | 0.167 |
| No | **Ref** |  |
| Sweets on Lollies consumption on a typical day |  |  |
| Yes | 1.08 (0.61-1.92) | 0.851 |
| No | **Ref** |  |
| Syrups and Jams consumption on a typical day |  |  |
| Yes | 0.93 (0.51-1.68) | 0.803 |
| No | **Ref** |  |
| Adding sugar to cereal, tea, coffee or milo on a typical day |  |  |
| Yes | 0.96 (0.63-1.46) | 0.856 |
| No | **Ref** |  |
| Salivary Mutans Streptococci |  |  |
| Low risk | 0.63 (0.43-0.92) | 0.100 |
| High risk | **Ref** |  |
| Salivary LB levels |  |  |
| Low risk | 0.70 (0.51-0.97) | 0.066 |
| High risk | **Ref** |  |

*P<0.05 in bold font*
