# Supplementary material for: Carious lesions in permanent dentitions are reduced in remote Indigenous Australian children taking part in a non-randomised preventive trial
Source: PLoS One. 2021 Jan 28;16(1):e0244927. doi: 10.1371/journal.pone.0244927 (PMC7842954; doi:10.1371/journal.pone.0244927)
Supplement: S1 File — (PDF) [file pone.0244927.s005.pdf]

Australian New Zealand Clinical Trials Registry

CREATE ACCOUNT LOGIN

DEFINITIONS HINTS AND TIPS FAQs REGISTER TRIAL MY TRIALS

Trial Review

VIEW TRIAL AT REGISTRATION

VIEW CURRENT TRIAL INFORMATION

The safety and scientific validity of this study is the responsibility of the study sponsor and investigators. Listing a study does not mean it has been endorsed by the ANZCTR. Before participating in a study, talk to your health care provider and refer to this information for consumers

< BACK

Trial registered on ANZCTR

**Registration number**	ACTRN12615000693527
**Ethics application status**	Approved
**Date submitted**	16/06/2015
**Date registered**	3/07/2015
**Date last updated**	24/05/2017
**Type of registration**	Prospectively registered

Titles & IDs

**Public title**	Preventing tooth decay in children in a remote community in Australia.
**Scientific title**	Effectiveness, cost-effectiveness and cost-benefit of a single annual professional intervention for the prevention of childhood dental caries in a remote rural Indigenous community.
**Secondary ID [1]**	Nil
**Universal Trial Number (UTN)**	U1111-1171-1262
**Trial acronym**	
**Linked study record**	

Health condition

Health condition(s) or problem(s) studied:

Dental Caries

Condition category	Condition code
Oral and Gastrointestinal	Other diseases of the mouth, teeth, oesophagus, digestive system including liver and colon
Public Health	Epidemiology

Intervention/exposure

Study type

Interventional

Description of intervention(s) / exposure

The dental prevention model will essentially reduce the microbial load with the topical disinfectant, povidone iodine; inhibit biofilm adherence to susceptible sites by application of fissure sealants and reduce the susceptibility of the tooth to demineralisation by acids generated in the microbial biofilm by the application of a fluoride varnish.

Topical Treatment by PVP-Iodine in conjunction with Fluoride Varnish is simple. The iodine comes in a single application swab. The total treatment time is 3 to 4 minutes and costs less than 20 cents. Clinically

the teeth are brushed to remove debris and disrupt the biofilm; then dried with gauze, and painted with 0.2 ml PVP-Iodine. After the iodine application, the teeth are dried again and coated with fluoride varnish at the same visit. Product Name: Povidone Iodine Pads and Swabsticks.

Fluoride varnishes are a liquid resin or synthetic base that contain a high concentration of fluoride and set quickly on contact with teeth, even in the presence of saliva. The fluoride varnish takes 3-5 minutes to apply. Fluoride ions in the material are released when the pH drops in response to acid production in the biofilm on the tooth surface and these become available to promote remineralisation of damaged tooth enamel in early carious lesions (white spots). The fluorhydroxyapatite formed over time during the remineralisation process in an initial caries lesion is more resistant to future demineralisation. Product Name: Colgate Durphat - Varnish Single Dose 5% Sodium Fluoride (22,600 ppm).

Dental decay most often occurs on the occlusal pits and fissures of permanent molar teeth. A pit and fissure sealant is defined as a material [both glass-ionomer and resin-based materials are widely used] that is introduced into the occlusal pits and fissures of caries susceptible teeth. Fissues sealant applications take about 15-30 minutes to complete. Product Name: Conseal-Clear, Conseal-Light Grey, Conseal F (White).

These interventions will be administered by a dentist or oral/dental health therapist, at baseline, years 1 and 2 of the study. All participants will receive the identical preventive intervention.

Intervention code [1] Prevention

Intervention code [2] Treatment: Other

Comparator / control treatment The expected caries increment will be modelled from the three oral health surveys conducted in this community (2004; 2012 and 2015) and compared with the actual caries increment from 2015-2016; 2016-2017 and 2015-2017. The mean caries increment will be compared between the expected (modelled) and actual findings, and adjusted for known risk factors for dental caries.

Control group Uncontrolled

Outcomes

Primary outcome [1] The International Caries Detection and Assessment system (ICDAS-II) for clinical caries diagnosis will be used to record caries experience.

Timepoint [1] This will be measured at the beginning of the study (baseline) and then 12 and 24 months later.

Primary outcome [2]
The International Caries Detection and Assessment system (ICDAS-II) for clinical caries diagnosis will be

Timepoint [2]
12 and 24 months after the baseline survey.

Secondary outcome [1]
General Child Quality of Life, the social impact of oral disorders and Oral Health-Related Quality of Life (OHRQoL).

Timepoint [1]
This will be measured at the beginning of the study (baseline) and then 12 and 24 months later.

Secondary outcome [2]
The retention of the fissure sealants at the follow-up periods will be assessed, and recorded via a visual clinical examination when the International Caries Detection and Assessment system (ICDAS-II) for clinical caries diagnosis is carried out. The fissure sealant is recorded as intact, partially intact or missing.

Timepoint [2]
12 and 24 months after the baseline survey.

Secondary outcome [3]
The pH of the oral environment is recorded by placing a piece of litmus paper in the mouth. Oral hydration is then visually assessed by observing saliva pooling in the floor of the mouth, by noting its constituency [watery, frothy or sticky], and by blotting the inside of the lower lip and timing how long it takes for beads of saliva to form from the minor salivary glands there. Stimulated saliva is then collected by spitting/dribbling into a cup whilst chewing on a piece of paraffin wax for five minutes. The volume is recorded.

A few drops of saliva/whole mouth fluid are pipetted from this reservoir onto Saliva-check Buffer papers (GC America Inc) to record buffering capacity. Caries Risk Test (CRT) kits (Ivoclar Vivadent, Australia) are used to further assess salivary buffering and the nutrient agar from these kits flooded with saliva and incubated for 48 hours at 37C to determine counts of Mutans streptococci?, Lactobacilli and yeasts.

Timepoint [3]
This will be measured at the beginning of the study (baseline) and then 12 and 24 months later.

Secondary outcome [4]
Resources use and costs of providing the intervention will be recorded throughout the intervention period. Resource use and costs to participants to receive the intervention (e.g. time off work to bring the child to the clinic) will be recorded as well as any emergency treatment required between annual visits by the team.

Timepoint [4]
In years 1, 2 and 3 of the project.

Eligibility

Key inclusion criteria
All children (approximately 600-650) attending the two primary and one secondary school campuses will be invited to participate in the intervention study.

**Minimum age**	4 Years
**Maximum age**	17 Years
**Gender**	Both males and females
**Can healthy volunteers participate?**	No
**Key exclusion criteria**	None

Study design

**Purpose of the study**	Prevention
**Allocation to intervention**	Non-randomised trial
**Procedure for enrolling a subject and allocating the treatment (allocation concealment procedures)**	All children (approximately 600-650) attending the two primary and one secondary school campuses will be invited to participate in the intervention study.
**Methods used to generate the sequence in which subjects will be randomised (sequence generation)**	
**Masking / blinding**	Open (masking not used)
**Who is / are masked / blinded?**	
**Intervention assignment**	Single group
**Other design features**	
**Phase**	Not Applicable
**Type of endpoint(s)**	Efficacy
**Statistical methods / analysis**	All baseline socio-demographic characteristics will be described for the study participants using counts and frequencies. Baseline and follow-up caries experience and questionnaire related information will be reported. Dental caries increment (incidence) will be the main outcome measure used to determine the effectiveness of the preventive intervention. The expected caries increment will be modelled from the

three oral health surveys conducted in this community (2004; 2012 and 2015) and compared with the actual caries increment from 2015-2016; 2016-2017 and 2015-2017. The mean caries increment will be compared between the expected (modelled) and actual findings, and adjusted for known risk factors for dental caries. The hypothesis will be that caries increment observed in the period of 2015 to 2017 is smaller than the modelled caries increments. Two independent samples t-test will be used for the analysis with significance being determined if $p<0.05$. The entire study population will be invited to participate and therefore no sample size calculation was determined.

Children who receive only a part of the intervention will be separately assessed: for example we will have children who fully participate, those with baseline and only a year 1 follow-up, those with baseline and only a year 2 follow-up. This will 'naturally' further inform us on the most appropriate frequency of this preventive strategy. Both a group and matched analysis will be conducted to account for children who receive only part of the intervention.

Development of Markov Model
A health state transition Markov model will be developed using Tree Age pro software (TreeAge Software Inc., Williamstown, Massachusetts, USA) to analyse the cost effectiveness of the intervention. The model will be populated with the caries experience of the children in NPA using the intervention caries data and compared with modelled data from baseline in a non-intervention scenario. The model cohort will start at the age of 6 years where mixed dentition is emerging. The model will track these children up to 17 years using the data from the study for each year. Health states for the model will include "healthy" and "caries" health states. It is anticipated to have health states for conditions such as pulpal abscess as well. The model will be made sensitive for waiting periods, available treatment facilities, availability and costs of resident or fly-in/fly-out professional staff and common practices of the local dental clinics.

Costs calculations
The costs of providing the preventive intervention, the costs of all treatment for carious lesions, and the out-of-pocket costs in relation to caries experience will be assessed. These costs will be assigned to each child taking into account the number of surfaces treated. Cost intervention will include sealants, an oral anti-septic application, application of a fluoride varnish and including cost for human resources and logistics. The costs of treating incremental caries will be estimated using government costs for treatments. Total out-of-pocket costs for parents of children with caries will be calculated based on the quantities of resource use provided in the surveys. Mean, median and interquartile range costs will be presented for each major treatment category in caries. All costs will be presented in 2015 AUD.

Estimation of Utility weights
The utility values for dental health states will be estimated from the CHU-9D data. Using the CHU-9D

(Child Health Utility) multi attribute utility instrument, quality of life scores (utility scores) for each caries severity level experienced by the children will be determined. A scoring algorithm that has been validated in the UK for CHU-9D for children will be applied. Utility scores will be presented for different age groups and gender. These values will contribute to estimate Quality Adjusted Life Years (QALYs). The CHU-9D will be validated in a similar Indigenous population prior to the application in the study population.

Transition probabilities
Caries increment prior to intervention and post intervention will be used to calculate transition probabilities respectively for the two scenarios of non-intervention and intervention examined by the Markov model. The caries increment rates for intervention will be directly observed. These rates for the non-intervention will be estimated from the modelled data.

Cost utility analysis
The cost utility of the “Big Bang” prevention strategy will be estimated using the Markov model. This analysis will adhere to the best modelling practices as given by ISPOR guidelines. All costs will be presented in 2015 AUD. Costs and outcomes will be discounted at 5% per year. The model will present the societal perspective. Incremental cost effectiveness ratio (ICER) will be generated by calculating incremental costs for caries treatment divided by the outcome (number of carious lesions prevented and QALYs gained separately). The intervention group will be compared with the modelled values for a non-intervention scenario. To address the uncertainty in the costs and effectiveness estimates, univariate sensitivity analyses will be used. For all probabilities, the 95% confidence intervals will be used, and for costs high and low values will be estimated. A probabilistic sensitivity analysis will also be performed by re-sampling 1000 times at random from the probability distributions for each parameter. This procedure is similar to multivariate sensitivity analysis and will address the uncertainty of all estimates simultaneously. Gamma distributions will be used for cost estimates and beta distributions will be used for probabilities.

Recruitment

Recruitment status

Active, not recruiting

Updated from
Not yet recruiting

Reason: All potential participants were invited; and 434 consented to the study. They all underwent the baseline clinical examination. About half consented to be treated for existing dental conditions and were provided the three preventive interventions. All the children who

consented for the study will be invited to be re-examined clinically to assess their oral health status, and all who accept this invitation will be provided the three preventive interventions again; and be referred to Queensland Health for treatment of any existing dental conditions. This is planned for October and November 2016.
Updated on 7/06/2016 10:05:10 AM

Date of first participant enrolment

Anticipated 10/08/2015

Actual 1/08/2015

Updated from

Reason: The actual date of first recruitment and last enrollment for baseline survey stays the same.
Updated on 7/06/2016 10:05:10 AM

Date of last participant enrolment

Anticipated 30/11/2015

Actual 30/11/2015

Updated from

Reason: The actual date of first recruitment and last enrollment for baseline survey stays the same.
Updated on 7/06/2016 10:05:10 AM

Date of last data collection

Anticipated 10/10/2017

Updated from

Reason: Year 3 data collection
Updated on 24/05/2017 4:48:22 PM

Actual

Sample size

**Target**	600	**Accrual to date**		**Final**	434
					**Updated from**
					**Reason:** Of those invited to participate, 434 consented. Updated on 7/06/2016 10:05:10 AM

Recruitment in Australia

Recruitment state(s) QLD

Funding & Sponsors

Funding source category [1] Government body

Name [1] National Health and Medical Research Council

Address [1] GPO Box 1421
CANBERRA ACT 2601

Country [1] Australia

Primary sponsor type University

Name Griffith University

Address Gold Coast campus
GRIFFITH UNIVERSITY QLD 4222

Country Australia

Secondary sponsor category [1] None

Name [1]

Address [1]

Country [1]

Ethics approval

**Ethics application status**	Approved
**Ethics committee name [1]**	Griffith University Human Ethics Committee
**Ethics committee address [1]**	Office for Research<br>Gold Coast campus<br>Room 4.25, The Learning Commons Building (G11)<br>Griffith University<br>Parklands Drive, Southport QLD 4215
**Ethics committee country [1]**	Australia
**Date submitted for ethics approval [1]**	
**Approval date [1]**	14/04/2015
**Ethics approval number [1]**	DOH/05/15/HREC
**Ethics committee name [2]**	Far North Queensland Humans Research Ethics Committee
**Ethics committee address [2]**	Level 7 East, 5B Sheridan Street Cairns QLD 4870<br>PO Box 902 CAIRNS Queensland 4870 Australia
**Ethics committee country [2]**	Australia
**Date submitted for ethics approval [2]**	
**Approval date [2]**	01/06/2015
**Ethics approval number [2]**	HREC/15/QCH/39 - 970

Summary

Brief summary

Background: The aim of the study is to reduce the high prevalence of tooth decay in children in a remote, rural Indigenous community in Australia, by application of a single annual dental preventive intervention. The study seeks to (1) assess the effectiveness of an annual oral health preventive intervention in slowing

the incidence of dental caries in children in this community, (2) identify the mediating role of known risk factors for dental caries and (3) assess the cost-effectiveness and cost-benefit of the intervention. Methods/Design: The intervention is novel in that most dental preventive interventions require regular re-application, which is not possible in resource constrained communities. While tooth decay is preventable, self-care and healthy habits are lacking in these communities, placing more emphasis on health services to deliver an effective dental preventive intervention. Importantly, the study will assess cost-benefit and cost-effectiveness for broader implementation across similar communities in Australia and internationally. Discussion: There is an urgent need to reduce the burden of dental decay in these communities, by implementing effective, cost-effective, feasible and sustainable dental prevention programs. Expected outcomes of this study include improved oral and general health of children within the community; an understanding of the costs associated with the intervention provided, and its comparison with the costs of allowing new lesions to develop, with associated treatment costs. Findings should be generalisable to similar communities around the world.

Trial website

Trial related presentations / publications

Lalloo R, Kroon J, Tut O, Kularatna S, Jamieson LM, Wallace V, Boase R, Fernando S, Cadet-James Y, Scuffham PA, Johnson NW. (2015) Effectiveness, cost-effectiveness and cost-benefit of a single annual professional intervention for the prevention of childhood dental caries in a remote rural Indigenous community. BMC Oral Health 15: 99. doi: 10.1186/s12903-015-0076-9.

Updated from

Reason: The protocol for the study has been published.
Updated on 7/06/2016 10:05:10 AM

Public notes

Contacts

Principal investigator

**Name**	Prof Newell W Johnson
**Address**	Building G40, Room 9.16, Gold Coast Campus, Griffith University, Queensland 4222
**Country**	Australia
**Phone**	61, 07, 56789306

Fax

Email n.johnson@griffith.edu.au

Contact person for public queries

Name Prof Newell W Johnson

Address Building G40, Room 9.16, Gold Coast Campus, Griffith University, Queensland 4222

Country Australia

Phone 61, 07, 56789306

Fax

Email n.johnson@griffith.edu.au

Contact person for scientific queries

Name Prof Newell W Johnson

Address Building G40, Room 9.16, Gold Coast Campus, Griffith University, Queensland 4222

Country Australia

Phone 61, 07, 56789306

Fax

Email n.johnson@griffith.edu.au

No information has been provided regarding IPD availability

Summary results

Have study results been published in a peer-reviewed journal?

Other publications

Have study results been made

publicly available in another format?

Results – basic reporting

Results – plain English summary

< BACK

ANZCTR

Home
About us
Statistics
Useful links
News
Contact
Privacy
Terms and conditions

Register a trial

Create account
Login
How to register a trial
How to update a trial
Data item definitions
Hints and tips
FAQs

Search for a trial

Find a trial
How to search
How to get involved

Major funders

Privacy | Disclaimer
Web design by G Squared

Copyright © Australian New Zealand Clinical Trials Registry. All rights reserved.
