# Supplementary material for: Carious lesions in permanent dentitions are reduced in remote Indigenous Australian children taking part in a non-randomised preventive trial
Source: PLoS One. 2021 Jan 28;16(1):e0244927. doi: 10.1371/journal.pone.0244927 (PMC7842954; doi:10.1371/journal.pone.0244927)
Supplement: S3 File — (PDF) [file pone.0244927.s007.pdf]

2015
Far North Queensland HREC EC00157

Research Study Checklist for Coordinating/Principal Investigators

All study documentation must have a version number, date and page numbers displayed in the footer

	A) Mandatory components for all submissions to an HREC	YES	No. of copies required
1.	**Cover letter,** (signed by Coordinating Principal Investigator) should include:<br>• Brief description of project (including phase of study if a clinical trial)<br>• List of all sites where study is to occur, applicable to this HREC application<br>• List of supporting documents submitted and uploaded onto online forms<br>• For commercially sponsored studies the name and address of the sponsor organisation/CRA for the HREC review invoice (must be Australian address) must be included in the cover letter	✓	14
2.	**For low and negligible risk (LNR) research studies –** A completed LNR application form accessed from: https://ethicsform.org/au/SignIn.aspx including all supporting documentation.<br>If you are accessing patient data including electronic data, medical records, e.g. auditing medical charts without patient consent and **request a waiver of consent**, LNR's will need to go to full HREC Review and 14 copies of the LNR including your entire submission is required.	☐<br>☐	1<br>14
3.	**For all other studies (NEAF):** Completed online NEAF with '*Submission Code"* accessed from online NEAF website: https://ethicsform.org/au/SignIn.aspx One NEAF with original signatures is required in addition to the 14 copies is mandatory.	✓	14
4.	**Mandatory Study protocol** (Although the protocol may have the same information as the NEAF or LNR application form, the protocol is the study working document. It is the formal design or specific plan for the research. When revisions occur during the course of the research you will need to submit a revised protocol as an amendment. The protocol should include a version date/number which is changed as the document is updated)	✓	14
5.	**Mandatory CV's** of researchers participating in the study (maximum 2 pages)	✓	14

	B) Other items that may be required (depending on the particular research project application being submitted)	YES	NO	N/A	No. of copies required
6.	**Data collection tool**(s) e.g CRF	✓	☐	☐	14
7.	**Master Participant Information Sheet and Consent Form** (PICF)	✓	☐	☐	14
8.	**CTN/CTX form**(s)	☐	☐	☐	1
9.	**Investigator's Brochure**	☐	☐	☐	14
10.	**Questionnaires/other instruments**	✓	☐	☐	14
11.	**Form of indemnity** (Medicines Australia HREC Review Only form) if HREC is not located at a participating site. (for industry sponsored studies)	☐	☐	☐	2
12.	**Advertising materials** (including a copy of transcript for advertisement, e-mail, website, letter or telephone call)	☐	☐	☐	14
13.	**Letter of invitation/Letter** to GP, etc.	☐	☐	☐	14
14.	**Participant diaries**	☐	☐	☐	14
15.	**Participant wallet card**	☐	☐	☐	14
16.	**Indigenous targeted research in remote communities requires a letter of support from Apunipima (Cape) or a relevant Indigenous Health Group.**	✓	☐	☐	14
	**Research using gene technology**				
17.	**Institutional Biosafety Committee (IBC) approval**	☐	☐	☐	14
18.	**Licence for dealings with a Genetically Modified Organism (GMO)**	☐	☐	☐	14
	**Research which is using radiological procedures that are performed specifically for research**				
19.	**Independent assessment report or verification by a Medical Physicist** (or District Radiation Safety Officer) of the total effective dose and relevant organ doses for those radiological procedures that are performed specifically for the research protocol	☐	☐	☐	14

Please ensure all documents are forwarded to the HREC Administrator collated and in sets

e.g Cover letter, NEAF or LNR, Letters of Support, Protocol, Consent Forms, Information Sheets, Data Collection Tools, Pamphlets/Posters & Researcher CV's ***= 1 set.***

Include this checklist with every new research project application

Assoc. Professor Jeroen Kroon
Deputy Head of School (Learning and Teaching)
Lead: Public Health, Community and Preventive Dentistry

School of Dentistry and Oral Health
Street address:
c/o Olsen & Parklands Dr, Southport,
Queensland, Australia, 4215

Postal address:
School of Dentistry and Oral Health,
Gold Coast Campus,
Griffith University,
Queensland 4222
Australia

Telephone +61 (0)7 5678 0742
Facsimile +61 (0)7 5678 0708
E-mail: j.kroon@griffith.edu.au
www.griffith.edu.au

Dr Paul Cullen
Chair: Far North Queensland Human Research Ethics Committee
PO Box 902
Cairns 4870

9 April 2015

Dear Dr Cullen

PROJECT TITLE: EFFECTIVENESS, COST-EFFECTIVENESS AND COST-BENEFIT OF A SINGLE ANNUAL PROFESSIONAL INTERVENTION FOR THE PREVENTION OF CHILDHOOD DENTAL CARIES IN A REMOTE RURAL INDIGENOUS COMMUNITY

A 2004 survey in the Northern Peninsula Area (NPA) of Far North Queensland, found that dental caries experience of 6- and 12-year-old children was more than twice that of the state average and more than four times greater than the comparable figures for Australian children overall. Soon after this survey the reticulated water supply of the five small rural communities in this area was fluoridated. A follow-up oral health survey in NPA conducted in November 2012 by this team, in which 70% of known resident schoolchildren we examined (n=339), suggests that the dental caries status has improved significantly since the 2004 survey. The fluoridation plant has been out of operation since April 2011 and the likelihood that the water will again be fluoridated is uncertain due to budget constraints and Queensland State Government legislated to give local governments the power to decide to fluoridate or not. Dental caries rates may again increase in the absence of water fluoridation.

The aim of this study, supported by a NHMRC project grant, is to reduce the high prevalence of tooth decay in children in a remote, rural Indigenous community in Australia, by application of a single annual dental preventive intervention, to identify the mediating role of known risk factors for dental caries and to assess the cost-effectiveness and cost-benefit of the intervention. Extensive community consultations with all stakeholders have already been completed and permission obtained from relevant authorities prior to commencement of the study.

The study will be conducted concurrently with the annual Queensland Health Well Persons' Health Check survey at the three NPA State College campuses. Treatment of children in need, as well as the preventive intervention, will be conducted in the dental clinic at Bamaga Hospital or in a Queensland Health mobile dental clinic.

The following supporting documentation is attached to this cover letter (numbered as per the checklist):

3. NEAF (Submission Code: AU/1/05FD114)
4. Study protocol
5. Researcher CV's
6. Data Collection Tool (ICDAS-II)
7. Participant Information Sheet and Consent Form (Parents/guardians, students)
10. Questionnaire
16. Letters of Support (Queensland Health Chief Dental Officer, Cape York Health Council (Apunipima), Queensland Health Torres and Cape Health and Hospital Service, Northern Peninsula Area Regional Council, Northern Peninsula Area State College).

Yours sincerely

Professor Jeroen Kroon
Coordinating Principal Researcher/Investigator

Online Forms
National Ethics Application Form

Within which Jurisdictions will your research application be submitted to: *(tick all that apply)*

- [ ] New South Wales
- [x] Queensland
- [ ] South Australia
- [ ] Victoria

HREC Application Reference Number: Griffith University HREC

1. TITLE AND SUMMARY OF PROJECT

1. Title

What is the formal title of this research proposal?
Effectiveness, cost-effectiveness and cost-benefit of a single annual professional intervention for the prevention of childhood dental caries in a remote rural Indigenous community

What is the short title / acronym of this research proposal (if applicable)?
Outcome of a single annual professional intervention on the prevention

2. Description of the project in plain language

Give a concise and simple description (not more than 400 words), in plain language, of the aims of this project, the proposal research design and the methods to be used to achieve those aims.

The aim of the study is to reduce the high prevalence of tooth decay in children in a remote, rural Indigenous community in Australia, by application of a single annual dental preventive intervention, to identify the mediating role of known risk factors for dental caries and to assess the cost-effectiveness and cost-benefit of the intervention.

Extensive community consultations with all stakeholders have been completed and permission obtained from relevant authorities prior to commencement of the study. These include the Community Elders, Queensland Health Chief Dental Officer, Cape York Health Council (Apunipima), Queensland Health Torres and Cape Health and Hospital Service (TCHHS), Northern Peninsula Area Regional Council (NPARC) and the Northern Peninsula Area State College (NPASC).

Following ethics approval by the Griffith University Human Research Ethics Committee (HREC), further ethics approval will be sought from the The Far North Queensland Human Research Ethics Committee (FNQHREC) and Queensland Government Department of Education and Training (DET). A research governance review will then lead to the project being provided with research authorisation by the Chief Executive Torres and Cape Hospital and Health Service (TCHHS) for implementation at TCHHS sites.

2. RESEARCHERS / INVESTIGATORS

1. Chief researcher(s)/investigator(s)
This question only applies to multi-centre research. If your research is not multi-centre, please leave this question blank. See Guidance Text (G) for the definition of a Chief Researcher

Chief researcher

	Title:	Forename/Initials:	Surname:
	Prof	Newell	Johnson
Mailing Address:	Menzies Health Institute of Queensland		
	Gold Coast Campus		

	Griffith University
Suburb/Town:	Southport
State:	QLD
Postcode:	4222
Country:	Australia
Organisation:	Griffith University
Department*:	Menzies Health Institute of Queensland
Position:	Emeritus Professor
E-mail:	n.johnson@griffith.edu.au
Phone (BH):	07 567 89306
Phone (AH)*:	
Mobile*:	0448 954 344
Pager*:	
Fax:	

Is this person the contact person for this application?
○ Yes ◉ No

Summary of qualifications and relevant expertise.
MDSc (Melb), PhD (Bristol), FDSRCS (Eng), FRACDS, FRCPath (UK), FFOP(RCPA), FICD, FILT, FMedSci

Fully registered dentist with registration as specialist in oral medicine, in oral pathology and in periodontology with the Australian Health Practitioner Authority. Formal research qualifications. Many years experience of epidemiological and clinical research involving children and adults, and of the examination of the state of the mouth and dentition as part of this. Experience of data entry and the analysis, interpretation, and publication of such results. Over 400 books, chapters and refereed publications reporting the results of these and other studies. Experience of such work in both urban and rural communities in Africa, India, Sri Lanka and Australia.

Please declare any general competing interests.
None.
As an investigator and as a clinician I will ensure that any treatment needs are recognised,and families advised on how to seek further care. In the first instance this will be referred to the dental clinic at Bamaga hospital, with staff funded from the project grant.

Name the site(s) for which this chief researcher / investigator is responsible.
Northern Peninsula Area (NPA) State College:
Injinoo Junior Campus (Pre-prep - Year 7)
Bamaga Junior Campus (Pre-prep - Year 7)
Bamaga Senior Campus

Describe the role of the chief researcher / investigator in this project.
I initiated and lead the baseline study of oral health in the NPA community, and performed much of the hands on work. The shape of the NHMRC grant was proposed by me, and developed with colleagues very experienced in relevant preventive dentistry and epidemiology. I will be the strategic manager of the project; of the budget; convene regular team meetings for monitoring progress [as I already do], and be hands-on at every site visit. I will convene brainstorms on data analysis and on where we seek to determine what our data teach us about effective and cost-effective ways to improve oral and general health in resource-poor communities.

Is the chief researcher / investigator a student? ○ Yes ◉ No

2. Principal researcher(s) / investigator(s)

Principal researcher / investigator 1

	Title:	Forename/Initials:	Surname:
	Prof	Ratilal	Lalloo
Mailing Address:	15/16 Mulyan Place		
Suburb/Town:	Ashmore		
State:	QLD		
Postcode:	4214		

Country:	Australia
Organisation:	Griffith University
Department*:	Dentistry and Oral Health
Position:	Professor (academic title)
E-mail:	r.lalloo@griffith.edu.au
Phone (BH):	0425248665
Phone (AH)*:	
Mobile*:	
Pager*:	
Fax:	

Is this person the contact person for this application?
◯ Yes ◉ No

Summary of qualifications and relevant expertise
BChD, BSc Med Hons (Epidemiol), MChD, PhD (London)

I am currently a Professor (academic title) in the School of Dentistry and Oral Health at Griffith University and Adjunct Associate Professor in the Australian Research Centre for Population Oral Health (ARCPOH) in the School of Dentistry at the University of Adelaide.

I was a Senior Research Fellow and the Acting Director of the Dental Practice Education Research Unit and Oral Health Promotion Clearinghouse at ARCPOH from April 2014 to March 2015. Prior to this I was the Colgate Chair and Professor: Rural, Remote and Indigenous Oral Health, in the School of Dentistry & Oral Health, Griffith University, Queensland, a position occupied from January 2009 to January 2014. Prior to this I was an academic at two dental schools in South Africa for almost 20 years. Over my proferssional career I have been extensively involved in the training of under- and post-graduate dental students, dental public health related research and management responsibilities at department, school and university levels. I am a dental public specialist, having completed his undergraduate degree in dentistry in 1986, an honours degree in Epidemiology in 1992, a Masters degree in Community Dentistry in 1994 and a PhD in Dental Public Health at the University College London in 2002, under the supervision of Professor Aubrey Sheiham.

I have published widely and my main research interest vary across many dental public health issues including preventive dentistry, evidence-based dentistry, oral health-related quality of life and health inequalities.

Please declare any general competing interests
None.
As an investigator and as a clinician I will ensure that any treatment needs are recognised,and families advised on how to seek further care. In the first instance this will be referred to the dental clinic at Bamaga hospital, with staff funded from the project grant.

Name the site(s) for which this principal researcher / investigator is responsible.
Northern Peninsula Area (NPA) State College:
Injinoo Junior Campus (Pre-prep - Year 7)
Bamaga Junior Campus (Pre-prep - Year 7)
Bamaga Senior Campus

Describe the role of the principal researcher / investigator in this project.
I will co-lead the proposed project, and manage its overall implementation. This will include involvement in initial and ongoing community and organisational consultations and discussions, ethics approval processes, recruitment of research assistants and delegation of work to research team, obtaining consent from all stakeholders including the parents and children, oral health screening, monitoring of intervention, dealing with adverse events, overseeing the capturing of the data, integral involvement in the data analysis, and the preparation of reports and publications.

Is the principal researcher a student? ◯ Yes ◉ No

Principal researcher / investigator 2

	Title:	Forename/Initials:	Surname:
	Prof	Jeroen	Kroon

Mailing Address: School of Dentistry and Oral Health
Gold Coast Campus
Griffith University

Suburb/Town: Southport
State: QLD
Postcode: 4222
Country: Australia
Organisation: Griffith University
Department*: School of Dentistry and Oral Health
Position: Public Health, Preventive and Community Dentistry
E-mail: j.kroon@griffith.edu.au
Phone (BH): 07 567 80742
Phone (AH)*:
Mobile*: 0418 650 248
Pager*:
Fax:

Is this person the contact person for this application?
(•) Yes ( ) No

Summary of qualifications and relevant expertise
BChD, BChD (Hons), DipHlthAdm (Dent), DipPubHlth (Dent), MChD (Comm Dent), DipTertInstr, PhD

I am the School of Dentistry and Oral Health Lead in Public Health, Community and Preventive Dentistry, a position held since March 2006. Prior to this I held an academic appointment at two dental schools in South Africa for almost 20 years. During this time I was extensively involved in the training of under- and post-graduate dental students, dental public health related research and management responsibilities at department, school and university levels. I am a dental public health specialist, having completed my undergraduate degree in dentistry in 1985, an honours degree in Epidemiology in 1987, a Masters degree in Community Dentistry in 1992 and a PhD in 2008. I have published widely and my main research interests include preventive dentistry, oral epidemiology and health economics. I have extensive experience in oral epidemiology in culturally diverse communities since graduating as a Specialist in Community Dentistry in 1992.

Please declare any general competing interests
None.
As an investigator and as a clinician I will ensure that any treatment needs are recognised,and families advised on how to seek further care. In the first instance this will be referred to the dental clinic at Bamaga hospital, with staff funded from the project grant.

Name the site(s) for which this principal researcher / investigator is responsible.
Northern Peninsula Area (NPA) State College:
Injinoo Junior Campus (Pre-prep - Year 7)
Bamaga Junior Campus (Pre-prep - Year 7)
Bamaga Senior Campus

Describe the role of the principal researcher / investigator in this project.
I was involved in the planning and implementation of the baseline study of oral health in the NPA community, and performed much of the hands on work. I was subsequently also closely involved in the current proposal, developed with colleagues experienced in the various fields required for this study.
I will be responsible for obtaining ethics approval for the project and will be part of the implementation of the project, analysis and interpretation of the data and writing up the results.

Is the principal researcher a student? ( ) Yes (•) No

Principal researcher / investigator 3

Title:	Forename/Initials:	Surname:
Mrs	Valda	Wallace

Mailing Address: Indigenous Centre
Cairns Campus
James Cook University
Suburb/Town: Cairns
State: QLD
Postcode: 4870
Country: Australia

Organisation:	James Cook University
Department*:	Indigenous Centre
Position:	Lecturer
E-mail:	valda.wallace@jcu.edu.au
Phone (BH):	07 423 21041
Phone (AH)*:	
Mobile*:	
Pager*:	
Fax:	

Is this person the contact person for this application?
○ Yes ◉ No

Summary of qualifications and relevant expertise
My qualifications include Associate Diploma Education (Adult Vocational),Bachelor of Teaching (Adult Vocational) and Master of Indigenous Studies.
I am a lecturer in Indigenous Studies and have worked with Aboriginal and Torres Strait Islander peoples both as colleagues and clients. I have previously worked in the health sector at Royal Darwin Hospital in the infection control office for a period of 9 years and I have also been a director on the Wuchopperen Health Service Board for a period of 6 years.Research projects I have been involved in include Indigenous Women's Homelessness, Gauging the value of Flexible Learning Centres, Improving Perinatal Care for indigenous Mothers and Impaired decision-making and Indigenous Queenslanders.

Please declare any general competing interests
None

Name the site(s) for which this principal researcher / investigator is responsible.
Northern Peninsula Area (NPA) State College:
Injinoo Junior Campus (Pre-prep - Year 7)
Bamaga Junior Campus (Pre-prep - Year 7)
Bamaga Senior Campus

Describe the role of the principal researcher / investigator in this project.
I am an Aboriginal woman with family ties to the Torres Strait Islander community; as such I will be providing advice from an Indigenous perspective and ensuring that the voice of Indigenous stakeholders and participants is core to the research. I will actively contribute to Study Management Committees and liaise with the research team in Bamaga to support the effective conduct of research.

Is the principal researcher a student? ○ Yes ◉ No

Principal researcher / investigator 4

	Title:	Forename/Initials:	Surname:
	A/Prof	Lisa	Jamieson

Mailing Address:	Australian Research Centre for Population Oral Health
	The University of Adelaide
	122 Frome Street (Cnr Pirie Street)
Suburb/Town:	Adelaide
State:	SA
Postcode:	5000
Country:	Australia
Organisation:	The University of Adelaide
Department*:	ARCPOH
Position:	Research Fellow
E-mail:	lisa.jamieson@adelaide.edu.au
Phone (BH):	08 8313 4858
Phone (AH)*:	
Mobile*:	
Pager*:	
Fax:	

Is this person the contact person for this application?

◯ Yes ◉ No

Summary of qualifications and relevant expertise
Bachelor of Dental Science, Master of Community Dentistry, PhD

Dr Jamieson hold vast experience of conducted studies in indigenous communities in Australia.

Please declare any general competing interests
None.
As an investigator and as a clinician I will ensure that any treatment needs are recognised, and families advised on how to seek further care. In the first instance this will be referred to the dental clinic at Bamaga hospital, with staff funded from the project grant.

Name the site(s) for which this principal researcher / investigator is responsible.
Northern Peninsula Area (NPA) State College:
Injinoo Junior Campus (Pre-prep - Year 7)
Bamaga Junior Campus (Pre-prep - Year 7)
Bamaga Senior Campus

Describe the role of the principal researcher / investigator in this project.
I will support the CIA in protocol development, monitoring and oversight of research conduct, analysis of results and drafting of research outputs. I will actively contribute to Study Management Committees and liaise with the research team in Bamaga to support the effective conduct of research.

Is the principal researcher a student? ◯ Yes ◉ No

Principal researcher / investigator 5

	Title:	Forename/Initials:	Surname:
	Dr	Ohnmar	Tut

Mailing Address:	Menzies Health Institute of Queensland
	Gold Coast Campus
	Griffith University
Suburb/Town:	Southport
State:	QLD
Postcode:	4222
Country:	Australia
Organisation:	Griffith University
Department*:	Menzies Health Institute of Queensland
Position:	Adjunct Senior Research Fellow
E-mail:	ohnmar.tut@griffith.edu.au
Phone (BH):	07 3161 6358
Phone (AH)*:	
Mobile*:	
Pager*:	
Fax:	

Is this person the contact person for this application?
◯ Yes ◉ No

Summary of qualifications and relevant expertise
BDS, MPhil

Dr Tut holds vast experience in the application of the proposed intervention in studies conducted in the Pacific Islands.

Please declare any general competing interests
None.
As an investigator and as a clinician I will ensure that any treatment needs are recognised, and families advised on how to seek further care. In the first instance this will be referred to the dental clinic at Bamaga hospital, with staff funded from the project grant.

Name the site(s) for which this principal researcher / investigator is responsible.
Northern Peninsula Area (NPA) State College:
Injinoo Junior Campus (Pre-prep - Year 7)
Bamaga Junior Campus (Pre-prep - Year 7)

Bamaga Senior Campus

Describe the role of the principal researcher / investigator in this project.
I was a main examiner of children in the 2012 survey. I will assist in planning, implementation and coordination of the study project, staff training on the intervention procedures (FV, sealant, iodine), and oral health education as required. I will actively contribute to Study Management Committees and liaise with the research team in Bamaga to support the effective conduct of research.

Is the principal researcher a student? ○ Yes ◉ No

Principal researcher / investigator 6

Title:	Forename/Initials:	Surname:
Dr	Sanjeewa	Kularatna

Mailing Address:	School of Medicine
	Logan Campus
	Griffith University
Suburb/Town:	Meadowbrook
State:	QLD
Postcode:	4131
Country:	Australia
Organisation:	Griffith University
Department*:	School of Medicine
Position:	Research Fellow
E-mail:	s.kularatna@griffith.edu.au
Phone (BH):	07 338 21567
Phone (AH)*:	
Mobile*:	0450574376
Pager*:	
Fax:	

Is this person the contact person for this application?
○ Yes ◉ No

Summary of qualifications and relevant expertise
BDS, MSc, MD

Completed a PhD in Health Economics.

Please declare any general competing interests
None.
As an investigator and as a clinician I will ensure that any treatment needs are recognised, and families advised on how to seek further care. In the first instance this will be referred to the dental clinic at Bamaga hospital, with staff funded from the project grant.

Name the site(s) for which this principal researcher / investigator is responsible.
Northern Peninsula Area (NPA) State College:
Injinoo Junior Campus (Pre-prep - Year 7)
Bamaga Junior Campus (Pre-prep - Year 7)
Bamaga Senior Campus

Describe the role of the principal researcher / investigator in this project.
I was involved in the planning stage of the proposal and provided my inputs in planning for economic analysis of the intervention.I will support the research team in determining costing of intervention, treatment and quality of life measurements. I will populate a decision analysis model to evaluate the intervention and produce cost effectiveness results. I will actively contribute to Study Management Committees and liaise with the research team in Bamaga to support the effective conduct of research.

Is the principal researcher a student? ○ Yes ◉ No

3. Associate Researcher(s) / investigator(s)

How many known associate researchers are there? (You will be asked to give contact details for these associate researchers / investigators) 4

Do you intend to employ other associate researchers / investigators? ◯ Yes ◉ No

Associate Researcher / Investigator 1

Title:	Forename/Initials:	Surname:
Prof	Paul	Scuffham

Mailing Address:	School of Medicine<br>Logan Campus<br>Griffith University
Suburb/Town:	Meadowbrook
State:	QLD
Postcode:	4131
Country:	
Organisation:	Griffith University
Department*:	School of Medicine
Position:	Chair in Health Economics
E-mail:	p.scuffham@griffith.edu.au
Phone (BH):	07 338 21367
Phone (AH)*:	
Mobile*:	
Pager*:	
Fax:	

Is this person the contact person for this application?
◯ Yes ◉ No

Summary of qualifications and relevant expertise
PhD, PGDip (Arts), BA

Please declare any general competing interests
None

Description of the role of the associate researcher / investigator in this project.
Prof Schuffham is a health economist and in this capacity he will provide input to the project.

Name the site at which the associate researcher / investigator has responsibility.
Prof Scuffham might not visit the NPA. He will provide his support to the project from Griffith University.

You have indicated that this research will be undertaken with Aboriginal and Torres Strait Islander communities. Is this researcher / investigator a cultural mentor, an interpreter or an analyst? ◯ Yes ◉ No

Is this associate researcher / investigator a student? ◯ Yes ◉ No

Associate Researcher / Investigator 2

Title:	Forename/Initials:	Surname:
A/Prof	Robyn	Boase

Mailing Address:	College of Medicine and Dentistry<br>Smithfield Campus<br>James Cook University
Suburb/Town:	Cairns
State:	QLD
Postcode:	4870
Country:	
Organisation:	James Cook University
Department*:	College of Medicine and Dentistry
Position:	Preventive Dentistry and Indigenous Oral Health
E-mail:	robyn.boase@jcu.edu.au
Phone (BH):	07 4042 1295

Phone (AH)*:

Mobile*:

Pager*:

Fax:

Is this person the contact person for this application?
○ Yes ◉ No

Summary of qualifications and relevant expertise
BDS, BSc, FRACDS (Children's Dentistry), Grad Cert Management

Please declare any general competing interests
None

Description of the role of the associate researcher / investigator in this project.
Advise the research team in her capacity as a previous Director Oral Health Services Cairns & Hinterland Health Service District. Currently employed at the College of Medicine and Dentistry, James Cook University as A/Prof in Preventive Dentistry and Indigenous Oral Health.

Name the site at which the associate researcher / investigator has responsibility.
A/Prof Boase might not visit the NPA. She will provide her support from James Cook University in Cairns.

You have indicated that this research will be undertaken with Aboriginal and Torres Strait Islander communities. Is this researcher / investigator a cultural mentor, an interpreter or an analyst? ○ Yes ◉ No

Is this associate researcher / investigator a student? ○ Yes ◉ No

Associate Researcher / Investigator 3

	Title:	Forename/Initials:	Surname:
	Prof	Yvonne	Cadet-James

Mailing Address:	Australian Aboriginal and Torres Strait Islander Studies
	Townsville Campus
	James Cook University
Suburb/Town:	Townsville
State:	QLD
Postcode:	4811
Country:	
Organisation:	James Cook University
Department*:	Australian Aboriginal and Torres Strait Islander S
Position:	Chair of Indigenous Australian Studies
E-mail:	yvonne.cadetjames@jcu.edu.au
Phone (BH):	07 47815594
Phone (AH)*:	
Mobile*:	
Pager*:	
Fax:	

Is this person the contact person for this application?
○ Yes ◉ No

Summary of qualifications and relevant expertise
Diploma of Applied Science, Bachelor of Nursing, Graduate Diploma of Adult Education

Please declare any general competing interests
None

Description of the role of the associate researcher / investigator in this project.
Advise the research team in her capacity as Chair of the Indigenous Australian Studies unit at James Cook University.

Name the site at which the associate researcher / investigator has responsibility.
Prof Cadet-James might not visit the NPA. She will provide his support to the project from James Cook University in Townsville.

You have indicated that this research will be undertaken with

Aboriginal and Torres Strait Islander communities. Is this researcher / investigator a cultural mentor, an interpreter or an analyst? (●) Yes ( ) No

Is this associate researcher / investigator a student? ( ) Yes (●) No

Associate Researcher / Investigator 4

Title:	Forename/Initials:	Surname:
Dr	Surani	Fernando

Mailing Address:	Menzies Health Institute of Queensland<br>Gold Coast Campus<br>Griffith University
Suburb/Town:	Southport
State:	QLD
Postcode:	4222
Country:	
Organisation:	Griffith University
Department*:	Menzies Health Institute of Queensland
Position:	Research Fellow
E-mail:	surani.fernando@griffith.edu.au
Phone (BH):	07 555 29636
Phone (AH)*:	
Mobile*:	
Pager*:	
Fax:	

Is this person the contact person for this application?
( ) Yes (●) No

Summary of qualifications and relevant expertise
BDS, MSc, MD

Please declare any general competing interests
None.
As an investigator and as a clinician I will ensure that any treatment needs are recognised, and families advised on how to seek further care. In the first instance this will be referred to the dental clinic at Bamaga hospital, with staff funded from the project grant.

Description of the role of the associate researcher / investigator in this project.
Clinical examination of research participants

Name the site at which the associate researcher / investigator has responsibility.
Northern Peninsula Area (NPA) State College:
Injinoo Junior Campus (Pre-prep - Year 7)
Bamaga Junior Campus (Pre-prep - Year 7)
Bamaga Senior Campus

You have indicated that this research will be undertaken with Aboriginal and Torres Strait Islander communities. Is this researcher / investigator a cultural mentor, an interpreter or an analyst? ( ) Yes (●) No

Is this associate researcher / investigator a student? (●) Yes ( ) No

What is the educational organisation, faculty and degree course of the student?

Organisation:	Griffith University
Faculty:	Dentistry and Oral Health
Degree course:	PhD

Is this research project part of the evaluation of the student? ( ) Yes (●) No

Is the student's involvement in this project elective or compulsory? (●) Elective ( ) Compulsory

What training has the student received in the | Research methodology training and workshops conducted by

relevant research methodology?	Higher Degree Research student center
What training has the student received in the ethics of research?	Student has applied for and obtained ethics approval from Griffith Human Research ethics committe
Describe the supervision to be provided to the student.	The student will be supervised during clinical examination of participants during field work by Professor Newell Johnson
How many supervisors does the student have?	4

Supervisor 1
Provide the name, qualifications, and expertise, relevant to this research, of the students' supervisor.

Title: Prof
First Name: Newell
Surname: Johnson
Summary of qualifications and relevant expertise
MDSc (Melb), PhD (Bristol), FDSRCS (Eng), FRACDS, FRCPath (UK), FFOP(RCPA), FICD, FILT, FMedSci

Fully registered dentist with registration as specialist in oral medicine, in oral pathology and in periodontology with the Australian Health Practitioner Authority. Formal research qualifications. Many years experience of epidemiological and clinical research involving children and adults, and of the examination of the state of the mouth and dentition as part of this. Experience of data entry and the analysis, interpretation, and publication of such results. Over 400 books, chapters and refereed publications reporting the results of these and other studies. Experience of such work in both urban and rural communities in Africa, India, Sri Lanka and Australia.

Supervisor 2
Provide the name, qualifications, and expertise, relevant to this research, of the students' supervisor.

Title: Prof
First Name: Paul
Surname: Scuffham
Summary of qualifications and relevant expertise
PhD, PGDip (Arts), BA

Prof Schuffham is a health economist and in this capacity he will provide input to the project.

Supervisor 3
Provide the name, qualifications, and expertise, relevant to this research, of the students' supervisor.

Title: A/Prof
First Name: Rodney
Surname: Lea
Summary of qualifications and relevant expertise
BSc (H), PhD

A/Prof Lea is not directly involved in this project

Supervisor 4
Provide the name, qualifications, and expertise, relevant to this research, of the students' supervisor.

Title: Dr
First Name: David
Surname: Speicher
Summary of qualifications and relevant expertise
PhD

Dr Speicher is not directly involved in this project

5. Other personnel relevant to the research project

5a. How many known other people will play a specified role in the conduct of this research project?

6

5b. Describe the role, and expertise where relevant (e.g. counsellor), of these other personnel.

A Project Manager
Two oral health professionals and two dental assistants appointed to the project to perform dental treatment and apply the preventive intervention
A member from the community to conduct the participant interviews

5c. Is it intended that other people, not yet known, will play a specified role in the conduct of this research project?

(•) Yes ( ) No

6. Certification of researchers / investigators

6a. Are there any relevant certification, accreditation or credentialing requirements relevant to the conduct of this research?

(•) Yes ( ) No

Describe the certification, accreditation or credentialing requirements.
Registration with the Dental Board of Australia

Specify and advise whether the principal researcher or any of the associate researchers have been so certified and/or accredited or credentialed.
All researchers / investigators involved in this project will hold current appropriate registration with the Dental Board of Australia prior to the commencement of the project

7. Training of researchers

7a. Do the researchers / investigators or others involved in any aspect of this research project require any additional training in order to undertake this research?

(•) Yes ( ) No

What is this training?
Calibration of examiners as to the criteria of the World Health Organisation’s standardised Oral Health Surveys protocols

How and by whom will the training be provided?
At Griffith University by Professor Newell Johnson
Ten per cent of subjects will be re-examined during the project to track intra-examiner reproducibility

How will the outcome of the training be evaluated?
Calculation of inter and intra examiner agreements according to a kappa scale of 0.8

3. RESOURCES

Project Funding / Support

1. Indicate how the project will be funded?

Type of funding.

[Please note that all fields in any selected funding detail column (with the exception of the code) will need to be completed.]

Funding	Confirmed or Sought?			
External Competitive Grant	(●) Confirmed	( ) Sought	( ) Not Sought	*Amount of funding* $1,020,590
Internal Competitive Grant	( ) Confirmed	( ) Sought	(●) Not Sought	
Sponsor	( ) Confirmed	( ) Sought	(●) Not Sought	
By Researchers Department or Organisation	( ) Confirmed	( ) Sought	(●) Not Sought	

1a. External Competitive Grant

Name of Grant / Sponsor	NHMRC Project Grant
Code (optional)	1081320
Detail in kind support	N/A
Indicate the extent to which the scope of the grant and the scope of this HREC application are aligned:	N/A

2. How will you manage a funding shortfall (if any)?

No shortfall is currently envisaged

3. Will the project be supported in other ways eg. in-kind support/equipment by an external party eg. sponsor?

(●) Yes ( ) No

Describe the support and indicate the provider:
The following facilities will be provided by Queensland Health for treatment of participants and application of the preventive intervention:
One surgery in the dental clinic at Bamaga Hospital
A one surgery mobile dental clinic

4. Is this a study where capitation payments are to be made, and will participants be made aware of these payments to clinicians or researchers / investigators?

No

Duality of Interest

5. Describe any commercialisation or intellectual property implications of the funding/support arrangement.

None

6. Does the funding/support provider(s) have a financial interest in the outcome of the research?

( ) Yes (●) No

7. Does any member of the research team have any affiliation with the provider(s) of funding/support, or a financial interest in the outcome of the research?

(●) Yes ( ) No

Describe affiliation(s) and/or interest(s):
Investigators hold academic appointments at either Griffith University, The University of Adelaide or James Cook University
Do you consider the relationship between the research team and the funding/support provider constitutes:

- [ ] a potential conflict of interest
- [ ] a potential duality of interest
- [x] no ethical issue

Provide an explanation:
NHMRC Project Grant

8. Does any other individual or organisation have an interest in the outcome of this research?

(•) Yes ( ) No

Indicate the interested party and describe the interest:
Queensland Health Directorate of Oral Health:
Outcome of an improvement in oral health following implementation and 2 year follow-up of the intervention is of interest to Queensland Health

Queensland Health Well Persons' Health Check survey team:
Any data on treatment needs as found in our survey will be shared with the Well Persons' Health Check survey team as part of the duty of care principle of epidemiological surveys of this nature

9. Are there any restrictions on the publication of results from this research?

( ) Yes (•) No

4. PRIOR REVIEWS

Ethical Review

Some HRECs may require researchers to provide information additional to that contained in a NEAF proposal. For this reason, it is prudent to check whether the HRECs to whom you propose to submit this proposal require additional information.

Duration and location

1. In how many Australian sites, or site types, will the research be conducted?

1

2. In how many overseas sites, or site types, will the research be conducted?

0

3. Provide the following information for each site or site type (Australian and overseas, if applicable) at which the research is to be conducted

1

Site / Site Type Name:	Northern Peninsula Area of Far North Queensland
Site / Site Type Location:	Northern Peninsula Area (NPA) State College:<br>Injinoo Junior Campus (Pre-prep to Year 7)<br>Bamaga Junior Campus (Pre-prep to Year 7)<br>Bamaga Senior Campus

4. Provide the start and finish dates for the whole of the study including data analysis

Anticipated start date: 01/07/2015 *(dd/mm/yyyy)*
Anticipated finish date: 30/06/2018 *(dd/mm/yyyy)*

5. Are there any time-critical aspects of the research project of which an HREC should be aware?

(•) Yes ( ) No

Describe the time-critical aspects:
As agreed with the Queensland Health Well Persons' Health Check survey team this study will be conducted simultaneously with their survey at the three school campuses. The initial data collection period will only be about 2 weeks. This will be followed by treatment of children in need as identified during the survey, as well as the application of the preventive intervention. This will be followed by a 1 and 2-year follow up data collection.

6. To how many Australian HRECs (representing site organisations or the researcher's / investigator's organisation) is it intended that this research proposal be submitted?

2
A list of NHMRC registered Human Research Ethics Committees (HRECs), along with their institutional affiliations and contact details is available on the NHMRC website at the following web address: http://www.nhmrc.gov.au/health_ethics/hrecs/overview.htm#d.

7. HRECs

HREC 1

Name of HREC:
Griffith University Human Research Ethics Committee (EC00162)

Provide the start and finish dates for the research for which this HREC is providing ethical review:
Anticipated start date or date range: 01/07/2015 *(dd/mm/yyyy)*
Anticipated finish date or date range: 30/06/2018 *(dd/mm/yyyy)*

For how many sites at which the research is to be conducted will this HREC provide ethical review?
1

Site 1

Name of Site: Northern Peninsula Area of Far North Queensland

Principal Researcher 1

Principal Researcher Name:
Prof Newell Johnson

HREC 2

Name of HREC:
Far North Queensland Human Research Ethics Committee (EC00157)

Provide the start and finish dates for the research for which this HREC is providing ethical review:

Anticipated start date or date range: 01/07/2015 *(dd/mm/yyyy)*

Anticipated finish date or date range: 30/06/2018 *(dd/mm/yyyy)*

For how many sites at which the research is to be conducted will this HREC provide ethical review?

1

Site 1

Name of Site: Northern Peninsula Area of Far North Queensland

Principal Researcher 1

Principal Researcher Name:

Prof Newell Johnson

Associate Researcher 1

Associate Researcher Name:

Prof Newell Johnson

8. Have you previously submitted an application, whether in NEAF of otherwise, for ethical review of this research project to any other HRECs?

○ Yes ◉ No

9. HRECs

Research conducted overseas

Peer review

11. Has the research proposal, including design, methodology and evaluation undergone, or will it undergo, a peer review process?

○ Yes ◉ No

Explain why the research proposal will not undergo a peer review process.

The researchers believe that the project has been peer reviewed as part of the successful NHMRC Project Grant application (see attached confirmation letter of grant success)

5. PROJECT

1. Type of Research

Tick as many of the following 'types of research' as apply to this project. Your answers will assist HRECs in considering your proposal. A tick in some of these boxes will generate additional questions relevant to your proposal (mainly because the National Statement requires additional ethical matters to be considered), which will appear in Section 9 of NEAF.

The project involves:

- [x] Research using qualitative methods
- [x] Research using quantitative methods, population level data or databanks, e.g survey research, epidemiological research
- [x] Clinical research
- [x] Research involving the collection and / or use of human biospecimens
- [ ] Genetic testing/research
- [ ] A cellular therapy
- [ ] Research on workplace practices or possibly impacting on workplace relationships
- [ ] Research conducted overseas involving participants
- [ ] Research involving ionising radiation
- [ ] Research involving gametes or use or creation of embryos
- [ ] None of the above

Does the research involve limited disclosure to participants?

( ) Yes (•) No

Does the research involve:

- [ ] Opt out approach
- [ ] Waiver
- [ ] None of the above

Research plan

2. Describe the theoretical, empirical and/or conceptual basis, and background evidence, for the research proposal, eg. previous studies, anecdotal evidence, review of literature, prior observation, laboratory or animal studies.

The intervention is novel in that most dental preventive interventions require regular re-application, which is not possible in resource constrained communities. While tooth decay is preventable, self-care and healthy habits are lacking in these communities, placing more emphasis on health services to deliver an effective dental preventive intervention. Importantly, the study will assess cost-benefit and cost-effectiveness for broader implementation across similar communities in Australia and internationally.

There is an urgent need to reduce the burden of dental decay in these communities, by implementing effective, cost-effective, feasible and sustainable dental prevention programs. Expected outcomes of this study include improved oral and general health of children within the community; an understanding of the costs associated with the intervention provided, and its comparison with the costs of allowing new lesions to develop, with associated

treatment costs. Findings should be generalisable to similar communities around the world.

As agreed with the Queensland Health Well Persons' Health Check survey team this study will be conducted simultaneously with their survey at the three school campuses. Any data on treatment need as found in our survey will be shared with the Well Persons' Health Check survey team as part of the duty of care principle of epidemiological surveys of this nature.

Globally four of the fifty most prevalent chronic diseases are related to oral health [1, 2]. Dental caries in the deciduous dentition is a significant predictor of dental caries in the permanent dentition [3] and is a preventable condition with adequate self-care, healthy lifestyle habits and preventive interventions at the health services level [4, 5]. Appropriate self-care is largely dependent on the social and health capital of the community [6]. This is often absent in rural and Indigenous communities. They also have the additional burden of limited services, a significant health burden and seriously reduced oral health-related and overall quality of life [7, 8].

Most dental services require professionally trained personnel and equipment regularly for the re-application of interventions. Several dental preventive interventions require re-application 2 or more a year [9, 10]. In disadvantaged and especially remote communities this is usually not possible. The result is that the burden of disease is never dealt with, nor prevented. There is currently no evidence on the effectiveness, cost-effectiveness and cost-benefit of a less frequent and therefore sustainable dental preventive intervention strategy.

A 2004 survey in the Northern Peninsula Area (NPA) of Far North QLD, found that dental caries experience of 6- and 12-year-old children was more than twice that of the state average and more than four times greater than the comparable figures for Australian children overall. Soon after this survey the reticulated water supply of the five small rural communities in this area was fluoridated. A follow-up oral health survey in NPA conducted in November 2012 by this team, in which 70% of known resident schoolchildren we examined (n=339), suggests that the dental caries status has improved significantly since the 2004 survey [11]. The fluoridation plant has been out of operation since April 2011 and the likelihood that the water will again be fluoridated is uncertain due to budget constraints and Queensland State Government legislated to give local governments the power to decide to fluoridate or not. Dental caries rates may again increase in the absence of water fluoridation.

The proposed study will implement a novel dental caries preventive intervention in children to (1) reduce the microbial load with the topical disinfectant, povidone iodine [10], (2) inhibit biofilm adherence to susceptible sites by application of fissure sealants [12] and (3) reduce the susceptibility of the tooth to demineralisation by acids generated in the microbial biofilm by the application of a fluoride varnish [9, 13] in a rural remote Indigenous community in Far North Queensland, Australia. While data are available on the effectiveness of the three specific interventions, our novel approach will assess the effectiveness and its cost effectiveness of a less frequent combined application of these interventions.

1. Vos T, et al: Years lived with disability (YLDs) for 1160 sequelae of 289 diseases and injuries 1990-2010: a systematic analysis for the Global Burden of Disease Study 2010. Lancet 2012, 380(9859):2163-2196.
2. Marcenes W, et al:: Global burden of oral conditions in 1990-2010: a systematic analysis. J Dent Res 2013, 92 (7):592-597.
3. Skeie MS, et al: The relationship between caries in the primary dentition at 5 years of age and permanent dentition at 10 years of age - a longitudinal study. Int J Paediatr Dent 2006, 16(3):152-160.
4. Selwitz RH, Ismail AI, Pitts NB: Dental caries. Lancet 2007, 369(9555):51-59.
5. Pihlstrom BL, Michalowicz BS, Johnson NW: Periodontal diseases. Lancet 2005, 366(9499):1809-1820.
6. Rouxel PL, et al: Social capital: theory, evidence, and implications for oral health. Community Dent Oral Epidemiol 2014, 10.1111/cdoe.12141.
7. Mejia GC, Parker EJ, Jamieson LM: An introduction to oral health inequalities among Indigenous and non-Indigenous populations. Int Dent J 2010, 60(3 Suppl 2):212-215.
8. Roberts-Thomson KF, Spencer AJ, Jamieson LM: Oral health of Aboriginal and Torres Strait Islander Australians. Med J Aust 2008, 188(10):592-593.
9. Marinho VC, et al: Fluoride varnishes for preventing dental caries in children and adolescents. Cochrane Database Syst Rev 2013, 7:CD002279.
10. Berkowitz RJ, et al: Adjunctive chemotherapeutic suppression of mutans streptococci in the setting of severe early childhood caries: an exploratory study. J Public Health Dent 2009, 69(3):163-167.
11. Johnson NW, et al: Effectiveness of water fluoridation in caries reduction in a remote Indigenous community in Far North Queensland. Aust Dent J 2014, 59(3):366-371.
12. Ahovuo-Saloranta A, et al: Sealants for preventing dental decay in the permanent teeth. Cochrane Database Syst Rev 2013, 3:CD001830.
13. Weintraub JA, et al: Fluoride varnish efficacy in preventing early childhood caries. J Dent Res 2006, 85(2):172-176.

3. State the aims of the research and the research question and/or hypotheses, where appropriate.

The study seeks to:
(1) assess the effectiveness of an annual oral health preventive intervention in slowing the incidence of dental caries in children in a remote, rural Indigenous setting;
(2) identify the mediating role of known risk factors for dental caries; and
(3) assess the cost-effectiveness and cost-benefit of the intervention.

We hypothesise that 1 and 2 years after the intervention:
(1) the actual incidence of dental caries in children will be significantly lower than the expected incidence, based on modelling from the two oral health surveys conducted over the past 11 years in the same community, the current survey and
(2) the intervention will be cost-effective and cost beneficial, and therefore feasible and sustainable for broader implementation across similar communities in Australia and internationally.

4. Has this project been undertaken previously?

( ) Yes (•) No

Benefits/Risks

In answering the following questions (Q 5 – 11) please ensure that you address all issues relevant to the type of participants that will be involved in your research project. Refer for guidance to relevant chapters of the National Statement.

5. Does the research involve a practice or intervention which is an alternative to a standard practice or intervention?

( ) Yes (•) No

6. Describe how the research demonstrates an understanding of and respect for and engages with the knowledge systems, cultural practices, heritage, beliefs, experiences and values of Aboriginal or Torres Strait Islander individuals and communities. Include, as appropriate:
-how the proposal responds to the diversity between communities eg. Different languages, cultures, histories, decision-making and perspectives (refer to Chapter 4.7 of the National Statement, Values and Ethics: Guidelines for Ethical Conduct in Aboriginal and Torres Strait Islander Health Research, and the AIATSIS Guidelines for Ethical Research in Indigenous Studies)
-how the proposal contributes to and does not erode social and cultural bonds among Aboriginal and Torres Strait Islander participants and communities,
-how the research respects the values based expectations and identity and protects and promotes cultural distinctiveness of Aboriginal and Torres Strait Islander people participants.

The 12 guidelines described in Chapter 4.7 of the NHMRC's National Statement on Ethical Conduct in Human Research on research with Aboriginal and Torres Strait Islander Peoples, the six values described in the NHMRC's document on Values and Ethics: Guidelines for Ethical Conduct in Aboriginal and Torres Strait Islander Health Research as well as the 14 principles described in AIATSIS' Guidelines for Ethical Research in Indigenous Studies have been circulated to all researchers and will be upheld during the implementation of the project.

All researchers involved in this project have many years of experience in oral epidemiological surveys in culturally diverse communities. As part of a recent visit of the Chief Investigator of the project, Professor Newell Johnson, to the NPA he met with the Community Elders, Cape York Health Council (Apunipima), Queensland Health Torres and Cape Health and Hospital Service (TCHHS), Northern Peninsula Area Regional Council (NPARC), Principal of the Northern Peninsula Area State College (NPASC) and staff from the NPA Primary Health Care Services who will be responsible for conducting the Well Persons' Health Check survey simultaneously with our survey. The project also has the support of the Queensland Health Chief Dental Officer (CDO). All of these parties have provided the project team with letters of support (see attached).

The survey team will respect the interests and aspirations of Aboriginal and Torres Strait Islander Peoples and will treat all partners as equal, notwithstanding that they may be different, through all stages of the research process. As part of this project there is no intention to do harm to Aboriginal and Torres Strait Islander individuals or communities and also to those things that they value. The overarching value of spirit and integrity that binds all others into a coherent whole in Aboriginal and Torres Strait Islander Peoples through time is well understood and will be respected by the survey team.

7. What expected benefits (if any) will this research have for the wider community?

A survey of the dental health of children in this community conducted in 2004 prior to the introduction of water fluoridation in 2005 showed that dental caries was a substantial problem: Caries experience for 6- and 12-year-old children was more than twice the state average and more than four times greater than the average figures for Australian children; the tercile of children with the highest caries experience were 7-8 times the national average; those in the top 10% had mean dmfs (decayed, missing and filled surfaces of teeth in the deciduous dentition) and DMFT (decayed, missing and filled teeth in the permanent dentition) scores more than 10 times that of the rest of Australia. A follow-up oral health survey in NPA conducted in November 2012 by this team, in which we examined over 70% of known resident schoolchildren (n=339), suggests that the dental caries status has improved significantly since the 2004 survey. However, significant unmet treatment needs exist at all ages.

There is an urgent need to reduce the burden of dental decay in these communities, by implementing effective, cost-effective, feasible and sustainable dental prevention programs. Expected outcomes of this study include improved oral and general health of children within the community; an understanding of the costs associated with the intervention provided, and its comparison with the costs of allowing new lesions to develop, with associated treatment costs. The work will benefit Indigenous children and reduce disparities. If found to be effective and cost-effective in reducing dental caries, this initiative could be implemented in similar communities elsewhere.

8. What expected benefits (if any) will this research have for participants?

As part of the duty of care principle of this survey information on treatment need will be shared with the Well Persons' Health Check survey team to ensure children will receive early intervention and/or immediate treatment. Participants will also be presented with general and oral health promotion activities.

Over and above this treatment being provided to participants, improved oral and general health of children within the community, an understanding of the costs associated with the intervention provided, and its comparison with the costs of allowing new lesions to develop with associated treatment costs, will benefit Indigenous children and reduce disparities.

9. Are there any risks to participants as a result of participation in this research project?

○ Yes ◉ No

10. Explain how the likely benefit of the research justifies the risks of harm or discomfort to participants.

Oral examinations will be conducted by calibrated dental surgeons registered with the Dental Board of Australia, with considerable experience of field epidemiology, based on the World Health Organisation's standardised Oral Health Surveys protocols. These protocols are no different to those of a standard oral examination with no additional risk of harm or discomfort to participants.

All treatment and application of preventive interventions will be provided by oral health professionals who hold full registration with AHPRA and the Dental Board of Australia in clinical facilities approved by the Chief Executive Torres and Cape Hospital and Health Service (TCHHS) as part of the research governance review of this project.

11. Are there any other risks involved in this research? eg. to the research team, the organisation, others

○ Yes ◉ No

12. Is it anticipated that the research will lead to commercial benefit for the investigator(s) and or the research sponsor (s)?

○ Yes ◉ No

16. Is there a risk that the dissemination of results could cause harm of any kind to individual participants - whether their physical, psychological, spiritual, emotional, social or financial well-being, or to their employability or professional relationships - or to their communities?

○ Yes ◉ No

Monitoring

17. What mechanisms do the researchers / investigators intend to implement to monitor the conduct and progress of the research project?

At least two of the chief researchers / investigators listed for this project will be present during the baseline and follow-up surveys to monitor the conduct and progress of the research project to ensure that the research conforms to the approved proposal.

All treatment and application of preventive interventions will be provided by oral health professionals who hold full registration with AHPRA and the Dental Board of Australia. This treatment will be monitored by the Project Manager and members of the project team.

18. Please detail your Data and Safety Monitoring Board (DSMB) and its nominee for this trial.

The Chief Investigators make up the Data and Safety Monitoring Board (DSMB) with Professor Newell Johnson (CIA) as its nominee for this project.

The DSMB:
(1) holds responsibility for monitoring the conduct of the approved research;
(2) will ensure that the project is conducted in compliance with the approved protocol; and
(3) will adhere to mechanisms for reporting and reviewing as specific by the HREC's who have approved this project.

6. PARTICIPANTS

1. Research participants

The National Statement identifies the need to pay additional attention to ethical issues associated with research involving certain specific populations.

This question aims to assist you and the HREC to identify and address ethical issues that are likely to arise in your research, if its design will include one or more of these populations. Further, the National Statement recognizes the cultural diversity of Australia’s population and the importance of respect for that diversity in the recruitment and involvement of participants. Your answer to this question will guide you to additional questions (if any) relevant to the participants in your study.

Tick as many of the following 'types of research participants’ who will be included because of the project design, or their inclusion is possible, given the diversity of Australia’s population. If none apply, please indicate this below.

If you select column (a) or (b), column (c) will not apply.

**The participants who may be involved in this research are:**<br><br>***If you select column (a) or (b), column (c) will not apply.***	**a) Primary intent of research**	**b) Probable coincidental recruitment**	**c) Design specifically excludes**
People whose primary language is other than English (LOTE)	☐	☑	☐
Women who are pregnant and the human fetus	☐	☑	☐
Children and/or young people (ie. <18 years)	☑	☐	

People in existing dependent or unequal relationships	☐	☐	☐
People highly dependent on medical care	☐	☐	☐
People with a cognitive impairment, an intellectual disability or a mental illness	☐	☑	☐
Aboriginal and/or Torres Strait Islander peoples	☑	☐	
People who may be involved in illegal activity	☐	☑	☐
None apply	☐		

You have indicated that it is probable that
- People whose primary language is other than English (LOTE)
- Women who are pregnant and the human fetus
- People with a cognitive impairment, an intellectual disability or a mental illness
- People who may be involved in illegal activity

may be coincidentally recruited into this project. The National Statement identifies specific ethical considerations for these groups(s).

Please explain how you will address these considerations in your proposed research.
People whose primary language is other than English:
The Principal of the NPA State College has confirmed that members of the community are available in the school who can interpret and assist with providing written consent

Women who are pregnant and the human foetus:
The basic oral examination to be conducted as part of this survey is a low risk procedure which poses no risk to either the mother or foetus. No X-rays will be taken as part of the protocol.

People with a cognitive impairment, an intellectual disability or a mental illness:
The school has a special needs unit on every campus and children from these units will be included in the survey. Should they not be able/willing to cooperate during the examination they will be excluded from the survey, but not discriminated against in terms of oral health care samples being provided to the participants

People who may be involved in illegal activity:
Although this does not form part of the survey we have a professional duty of care towards children who disclose substance abuse, smoking and use of alcohol. The Well Persons' Health Check team will be informed of any such cases

Participant description

2. How many participant groups are involved in this research project?

1

3. What is the expected total number of participants in this project at all sites?

Approximately 650

4. Groups

Group 1

Group name for participants in this group: Students at Northern Peninsula Area (NPA) State College
Expected number of participants in this group: 600
Age range: 6 to 17

Other relevant characteristics of this participant group:
It is expected that the majority of participants will be of Aboriginal and Torres Strait Islander descent
Why are these characteristics relevant to the aims of the project?
Both the baseline and follow-up studies conducted in 2004 and 2012 respectively were conducted in this age range as well

Participant experience

6. Provide a concise detailed description, in not more than 200 words, in terms which are easily understood by the lay reader of what the participation will involve.

All children (approximately 600-650) attending the three primary and one secondary school campuses of the Northern Peninsula Area State College will be invited to participate in the intervention study. These children will be 6-17 years of age, and almost all are Indigenous. All participants will have been given consent for participation in the study.

Relationship of researchers / investigators to participants

7. Specify the nature of any existing relationship or one likely to rise during the research, between the potential participants and any member of the research team or an organisation involved in the research.

None

9. Describe what steps, if any, will be taken to ensure that the relationship does not impair participants' free and voluntary consent and participation in the project.

Not applicable

10. Describe what steps, if any, will be taken to ensure that decisions about participation in the research do not impair any existing or foreseeable future relationship between participants and researcher / investigator or organisations.

All information of participants is confidential and will only be disclosed to the Well Persons' Health Check survey team as part of the duty of care principle of this project. Treatment will be provided to children in need as part of the project protocol.

11. Will the research impact upon, or change, an existing relationship between participants and researcher / investigator or organisations?

○ Yes ◉ No

12. Is it intended that the interview transcript will be shown or made available to participants?

◉ Yes ○ No

Why is it considered important that participants have access to this information?
Respect for the participants requires that the accuracy or completeness of each questionnaire should be verified by the relevant participant.

Recruitment

13. What processes will be used to identify potential participants?

All primary and secondary schoolgoing children who are enrolled at the Northern Peninsula Area State College will be considered as potential participants

14. Is it proposed to 'screen' or assess the suitability of the potential participants for the study?

( ) Yes (•) No

15. Describe how initial contact will be made with potential participants.

As agreed with the Queensland Health Well Persons' Health Check survey team this study will be conducted simultaneously with their survey at the three school campuses. Information and the informed consent mechanism for this project will be distributed to parents/guardians and the same time as the Well Persons' Health Check survey team information being sent to parents/guardians.

16. Do you intend to include both males and females in this study?

(•) Yes ( ) No

What is the expected ratio of males to females that will be recruited into this study and does this ratio accurately reflect the distribution of the disease, issue or condition within the general community?
The 2012 study consisted of 53% males and 47% females. It is expected to be similar in this study.

17. Is an advertisement, e-mail, website, letter or telephone call proposed as the form of initial contact with potential participants?

(•) Yes ( ) No

Provide details and a copy of text/script
As agreed with the Queensland Health Well Persons' Health Check survey team this study will be conducted simultaneously with their survey at the three school campuses. Information and the informed consent mechanism for this project will be distributed to children and parents/guardians at the same time as the Well Persons' Health Check survey team information being sent to parents/guardians (see attached).

18. If it became known that a person was recruited to, participated in, or was excluded from the research, would that knowledge expose the person to any disadvantage or risk?

( ) Yes (•) No

Consent process

Do you propose to obtain consent from individual participants for your use of their stored data/samples for this research project?
(•) Yes ( ) No

7. Participants Specific

Children or young people

You have indicated that the project involves children and/or young people.

10. Why is participation of children or young people indispensable to this research?

How has this study been designed to be appropriate for children or young people?
Oral examinations will be conducted based on the World Health Organisation’s standardised Oral Health Surveys protocols for this age cohort

11. Explain why there is no reason to believe that the research participation is not contrary to the best interests of the children or young people.

The methodology employed in this project is no different to that of a standard oral examination, treatment protocols, and protocols for the application of preventive interventions, all of which include proper infection control measures. Early diagnosis of disease is a recognised public health measure to ensure early intervention and arrest of the disease process.

12. Explain why the consent of the child/young person will not be sought.

The research prefers that informed consent be granted by the parent/guardian

13. Explain why the consent of the parent/guardian will not be sought.

Not applicable

8. CONFIDENTIALITY/PRIVACY

Answers to the questions in section 8.1 will establish whether an HREC will need to apply guidelines under federal or State/territory privacy legislation in reviewing your application. Answers to questions in the remaining parts of section 8 will show how confidentiality of participants is to be protected in your research.

1. Do privacy guidelines need to be applied in the ethical review of this proposal?

Indicate whether the source of the information about participants which will be used in this research project will involve:

- [x] collection directly from the participant
- [ ] collection from another person about the participant
- [ ] use or disclosure of information by an agency, authority or organisation other than your organisation
- [ ] use of information which you or your organisation collected previously for a purpose other than this research project

Information which will be collected for this research project directly from the participant

Describe the information that will be collected directly from participants. Be specific where appropriate.
A questionnaire on basic demography (gender and age), residential history, own general and oral health perceptions, oral health behaviour, attitude and knowledge, dental visits, diet and oral health-related quality of life. This questionnaire will be administered by a trained member of the community.

Determination of oral health status and treatment need following clinical examination.

The information collected by the research team about participants will be in the following form(s). Tick more than one box if applicable.

- [x] individually identifiable
- [ ] re-identifiable
- [ ] non-identifiable

Give reasons why it is necessary to collect information in individually identifiable or re-identifiable form
Any data on treatment need as found in our survey will be shared with the Well Persons' Health Check survey team as part of the duty of care principle of epidemiological surveys and to ensure treatment is provided

1c. Will the information to be used in medical research?

( ) Yes (•) No

1d. Does this application include an attachment relevant to state/territory privacy legislation?

○ Yes ◉ No

1e. Is the information health information?

◉ Yes ○ No

Using information from participants

2. Describe how information collected about participants will be used in this project.

The International Caries Detection and Assessment system (ICDAS-II) for clinical caries diagnosis will be used to record caries experience and to determine incidence. This will be measured annually across the 3 years of the study, at baseline and after years 2 and 3 of the project.

General Child Quality of Life, the social impact of oral disorders and Oral Health-Related Quality of Life (OHRQoL) will be measured at baseline and at years 2 and 3. Existing validated and reliable instruments will be used (CHU-9D, OHIP-14 and Child-OIPD), appropriately modified for our participants.

The retention of the fissure sealants at the follow-up periods will be assessed and recorded. Saliva of the participants will be collected again in years 2 and 3. Findings will be compared to baseline to assess the impact of the less frequently applied anti-bacterial component of the intervention.

Any data on treatment need as found in our survey will be shared with the Well Persons' Health Check survey team to ensure immediate treatment.

3. Will any of the information be used by the research team be in identified or re-identifiable (coded) form?

◉ Yes ○ No

Indicate whichever of the following applies to this project:

- [ ] Information collected for, used in, or generated by, this project will not be used for any other purpose.
- [x] Information collected for, used in, or generated by, this project will/may be used for another purpose by the researcher for which ethical approval will be sought.
- [ ] Information collected for, used in, or generated by, this project is intended to be used for establishing a database/data collection/register for future use by the researcher for which ethical approval will be sought.
- [ ] Information collected for, used in, or generated by, this project will/may be made available to a third party for a subsequent use for which ethical approval will be sought.

4. List ALL research personnel and others who, for the purposes of this research, will have authority to use or have access to the information and describe the nature of the use or access. Examples of others are: student supervisors, research monitors, pharmaceutical company monitors.

The project team
The Queensland Health Well Persons' Health Check survey team
Staff from the Queensland Health Oral Health Services in the NPA

Storage of information about participants during and after completion of the project

5. In what formats will the information be stored during and after the research project? (eg. paper copy, computer file on floppy disk or CD, audio tape, videotape, film)

During the data collection phase all information will be on paper and stored with the principal researcher. If time allows during the data collection phase the information will be captured electronically. The paper copies will then be brought back to the School of Dentistry and Oral Health, Griffith University, where the outstanding information will be captured electronically and the paper copies secured in a locked cabinet in the office of one of the chief

investigators. The electronic data will be saved on at least two computers of the chief investigators and a back-up copy on an external hard drive of a chief investigator.

6. Specify the measures to be taken to ensure the security of information from misuse, loss, or unauthorised access while stored during and after the research project? (eg. will identifiers be removed and at what stage? Will the information be physically stored in a locked cabinet?)

All paper copies of the survey will be secured in a locked cabinet in a lockable office of a chief investigator. Electronic copies of the data will be saved to at the university computers of two chief reseachers and a back-up copy to an external hard drive of a chief investigator. Three copies are considered appropriate in case of computer failure or damage. Names and surnames will not be captured electronically, only an identifier number will be entered to link the electronic database to the paper copy.

9. The information which will be stored at the completion of this project is of the following type(s). Tick more than one box if applicable.

- [x] individually identifiable
- [ ] re-identifiable
- [ ] non-identifiable

Give reasons why it is necessary to store information in individually identifiable or re-identifiable form.
Data will be made available to the Well Persons' Health Check survey team and staff from the Queensland Health Oral Health Services in the NPA to ensure prompt treatment of the children where a need has been identified

10. For how long will the information be stored after the completion of the project and why has this period been chosen?

The paper copies will be securely stored for 5-years (the minimum data retention period) from the date reports and academic publications are in the public domain. The chief investigators will authorise for all paper copies to be destroyed through shredding and then appropriatedly discarded. The electronic database will be kept indefinitely as any ability to match individual information to a person will no longer be possible once the paper copies are destroyed and the data may need to re-analysed at a later stage for comparison to follow-up studies.

11. What arrangements are in place with regard to the storage of the information collected for, used in, or generated by this project in the event that the principal researcher / investigator ceases to be engaged at the current organisation?

All information collected and generated by this project will be passed to one of the other chief investigators in the event of one of the chief investigators leaving the current organisation

Ownership of the information collected during the research project and resulting from the research project

12. Describe how the research will respect and acknowledge the contribution of Aboriginal or Torres Strait Islander peoples to the research. Include, as appropriate:
- acknowledgement of cultural property rights in relation to knowledge, ideas, cultural expressions and cultural materials,
- acknowledgement of the sources of information and those who have contributed to the research
- a description of any agreement (preferably written) between the researchers / investigators and the community regarding research intentions, methods and potential results.

The project team will fully respect and acknowledge the contribution of the community in all reports and publications that emanate from the project. This acknowledgement will relate to the community and its governmental and non-governmental organisations. The roles of the community and representative organisations will be appropriately acknowledged as all reports and publications. The written text of this acknowledgement will be informed and negotiated with the elders of the community and its representative organisations.

A written agreement will be entered into between the researchers and the community and its representative organisations regarding the research intentions, methods and reporting of the results of the project.

13. Who is understood to own the information resulting from the research, eg. the final report or published form of the results?

The chief investigators own the information and the reports and publications of the results.

14. Does the owner of the information or any other party have any right to impose limitations or conditions on the publication of the results of this project?

○ Yes ◉ No

Disposal of the information

15. Will the information collected for, used in, or generated by this project be disposed of at some stage?

◉ Yes ○ No

At what stage will the information be disposed?
All paper copies will be disposed of after 5-years from date of publication of the project. The electronic database, which will not be identified, will be indefinitely retained for future scholarly analysis.
How will information, in all forms, be disposed?
Paper copies will be shredded and discarded

Reporting individual results to participants and others

16. Is it intended that results of the research that relate to a specific participant be reported to that participant?

○ Yes ◉ No

Explain/justify why results will not be reported to participants:
Results of individual participants will be provided to the Queensland Health Well Persons' Health Check survey team to ensure appropriate treatment is delivered as part of the project protocol

17. Is the research likely to produce information of personal significance to individual participants?

◉ Yes ○ No

18. Will individual participant's results be recorded with their personal records?

◉ Yes ○ No

19. Is it intended that results that relate to a specific participant be reported to anyone other than that participant?

◉ Yes ○ No

To whom will the results be reported other than the participant?
Queensland Health Well Persons' Health Check survey team
Queensland Health Oral Health Services responsible for providing treatment to these children
Explain why the results will be reported to a person other than the participant?
Oral health treatment where identified
Will the participant be told that their results will be reported to another person?
◉ Yes ○ No

20. Is the research likely to reveal a significant risk to the health or well being of persons other than the participant, eg

family members, colleagues

○ Yes ◉ No

21. Is there a risk that the dissemination of results could cause harm of any kind to individual participants - whether their physical, psychological, spiritual, emotional, social or financial well-being, or to their employability or professional relationships - or to their communities?

○ Yes ◉ No

22. How is it intended to disseminate the results of the research? eg report, publication, thesis

Peer reviewed publications
A report to the Queensland Health Well Persons' Health Check survey team
A report to the Queensland Health Chief Dental Officer
Parent/guardians are invited to access the results from the project team or one of these organisations when it becomes available

23. Will the confidentiality of participants and their data be protected in the dissemination of research results?

◉ Yes ○ No

Explain how confidentiality of participants and their data will be protected in the dissemination of research results:
Data will be de-identified and presented as grouped results

9. PROJECT SPECIFIC

Your responses to question 5.1 "Type of Research" and question 6.1 "Research participants" indicate that the HREC will require additional information which is specific to your research project. The following table indicates the question sets relating to the project that you will need to complete. If this is not correct please return to question 5.1 and 6.1 at to amend your answer.

- 9.1. Type of research/trial
- 9.2. Clinical research
- 9.5. Research involving the collection and / or use of human biospecimens
- 9.7. Research Involving Aboriginal and Torres Strait Islander Peoples

9.1 Type of research/trial

1. The study involves:

- [x] The administration of a drug / medicine (includes a complementary / alternative medicine)
- [ ] The use of a medical device
- [ ] The administration of human somatic cell gene therapy
- [ ] The use of a xenotransplant
- [ ] The use of stem cells (adult or embryonic) as therapy
- [ ] Other

2. The project will be conducted as follows:

Under the Clinical Trial Notification Scheme (CTN) — ○ Yes ◉ No

Under the Clinical Trial Exemption Scheme (CTX) — ○ Yes ◉ No

You have indicated that you are conducting a clinical trial under neither the CTN or CTX scheme. Please ensure that this is correct by referring back to your answer at Page 16, Section 5, Question 1 'Type of Research' If you are conducting a trial in clinical setting, which will not take place under CTN or CTX, please ensure that enough detail has been provided about the research to allow a HREC to adequately review it. This may require you to review your answers in Page 16, Section 5, Quest Type of Research and/or Page 20, Section 6, Question 1 Research participants

3. Provide the following details for the clinical trial protocol:

Protocol name:	Effectiveness, cost-effectiveness and cost-benefit of a single annual professional intervention for
Protocol version number:	N/A
Protocol version date:	01/03/2015 *(dd/mm/yyyy)*

*If you intend to/have registered this trial in a publicly accessible register, please provide the details of it here*Prior to the annual intervention the research team will undertake clinical examination of all consenting school-age children to assess dental caries experience. A team comprising of a dentist and/or oral health therapist will treat all existing tooth decay and other oral health problems. Each child will receive the preventive intervention when other treatments are completed.

The proposed preventive intervention aims to reduce the pathogenic bacterial load, seal grooves on posterior (molar) teeth and strengthen the tooth structure.

All treatment and preventive intervention procedures are conducted in accordance with standard Australian protocols.

4. Provide the following details for the investigator's brochure/product information (as relevant):

Title of Investigator's Brochure:	Project Information Sheet (see attached)
Investigator's brochure version number:	N/A
Investigator's brochure version date:	01/03/2015 *(dd/mm/yyyy)*

9.2 Clinical research

1. The study examines:

- [x] The administration of a drug / medicine (includes a complementary / alternative medicine)
- [ ] The use of a medical device
- [ ] Other

2. Provide the following details for the study protocol:

Protocol title:	Effectiveness, cost-effectiveness and cost-benefit of a single annual professional intervention for
Protocol version number:	N/A
Protocol version date:	01/03/2015 *(dd/mm/yyyy)*

3. Provide a statement addressing the following as may be applicable to the project.

a) Method of randomisation
b) Whether the hypothesis offers a realistic possibility that the intervention is at least as effective as standard treatment

c) The justification for the use of placebo or non-treatment control group, including alternative effective treatments and any risk of harm in the absence of treatment.
d) How variations in response will be treated
e) Endpoints
f) Details of contingencies and management of these
g) Explain the arrangements in place to ensure there is adequate compensation for participants.

This is a longitudinal preventive intervention study. All school children in the NPA will be invited to participate. As it is unethical to withhold any proven intervention from any child, no control group will be created. Children who do not consent to participate may be natural controls if they consent to a dental examination at the end of the study. The actual caries increment in the children who participate will be compared to the expected caries increment modelled on oral health surveys carried out in this community in 2004 (pre-water fluoridation); in 2012 (by the Griffith University team post-partial water fluoridation) and 2015 (baseline survey for this study). We hypothesise that 1 and 2 years after the intervention:
1) the actual incidence of dental caries in children will be significantly lower than the expected incidence, based on modelling from the two oral health surveys conducted over the past 11 years in the same community, the current survey; and
2) the intervention will be cost-effective and cost beneficial, and therefore feasible and sustainable for broader implementation across similar communities in Australia and internationally.All school children in the NPA will be invited to participate. As it is unethical to withhold any proven intervention from any child, no control group will be created.   The use of a placebo does not form part of the project protocol.N/AAll active disease will be treated prior to implementing the dental caries preventive intervention. In years 2 and 3 of the study, all participating children will be invited to return for a dental examination, treatment of new incident disease and repeat of the prevention regime.The project team will be responsible for the management of contingencies withing the funding and protocol constraints of the project designN/A

4. How many drugs will be used in this research project?

4

5. Provide the following information for each drug:

Drug 1

Approved name:	Restorative dental materials required in the treatment of dental caries
Trade name:	Various
Approved therapeutic indication, dose and duration in Australia:	As per approved Australian standards
Dosage regimen:	As per approved Australian standards
Known adverse effects:	None
Known contra-indications/warnings:	As per approved Australian standards
Concurrent drugs to be avoided:	None

Drug 2

Approved name:	Pit and Fissure Sealants
Trade name:	Conseal f; Fuji VII
Approved therapeutic indication, dose and duration in Australia:	Conseal f: http://www.sdi.com.au/en-au/conseal-f/<br><br>Fuji VII: http://www.gcasia.info/proddet.asp?prodid=17
Dosage regimen:	As per approved Australian standards
Known adverse effects:	None
Known contra-indications/warnings:	As per approved Australian standards
Concurrent drugs to be avoided:	None

Drug 3

Approved name:	Fluoride Varnish
Trade name:	Colgate Duraphat Single Dose
Approved therapeutic indication, dose and duration in Australia:	http://www.colgateprofessional.com.au/products/Colgate-Duraphat-Varnish-Single-Dose/specifics
Dosage regimen:	0.4 ml of a 5% Sodium Fluoride as a single topical application
Known adverse effects:	None
Known contra-indications/warnings:	As per approved Australian standards
Concurrent drugs to be avoided:	None

Drug 4

Approved name:	Povidone (PVP) Iodine
Trade name:	PDI Povidone-Iodene Swabsticks (1's)
Approved therapeutic indication, dose and duration in Australia:	http://pdihc.com/all-products/pvp-iodine-prep-pads-and-swabsticks
Dosage regimen:	0.2ml of a 1% iodine (10% PVP) solution as a single topical application
Known adverse effects:	None
Known contra-indications/warnings:	As per approved Australian standards
Concurrent drugs to be avoided:	None

9.5 Research involving the collection and / or use of human biospecimens

You have indicated that the project involves the use of human biospecimens.

1. What is the nature of biospecimen/s you plan to use?

Prior to the treatment of existing disease we will investigate saliva of the participants as a component of caries risk assessment

2. What is the source of the biospecimen/s you wish to use? (tick all boxes that apply)

- [ ] Collected from participants recruited to this research project who are not concurrently undergoing diagnosis or treatment
- [x] Collected from participants recruited to this research project who are concurrently undergoing diagnosis or treatment
- [ ] Obtained from biobank/s
- [ ] Obtained from clinical pathology service/s
- [ ] Obtained from other researcher/s directly
- [ ] Obtained from health care provider/s directly
- [ ] Obtained from research institute/s
- [ ] Obtained from commercial entity/ies
- [ ] Obtained from, or accessed during, autopsy
- [ ] Sourced / obtained from overseas

2a. By whom will the biospecimen/s be collected?

- (selected) A member of the research team
- ( ) A third party

2b. Is the nature, volume and quantity of the biospecimen/s collected from each participant different/additional to that collected for standard diagnostic or therapeutic purposes for that participant? Please detail how these biospecimen/s will be collected.

( ) Yes (selected) No

Please detail how these biospecimen/s will be collected.

Saliva will be collected using commercially available test kits for measurement of flow rate, pH, buffering capacity and then cultured for bacterial assessment

3. In what form will the biospecimen/s be used by the investigators in the conduct of this project?

Biospecimen/s will be individually identified

Does the proposed research have the potential to reveal information that may be important for the health of the donor/s, their blood relatives or their community?

( ) Yes (selected) No

4. Will the biospecimen/s used for this project be destroyed once the project is completed?

(selected) Yes ( ) No

5. Does this research involve the development of a cell line?

( ) Yes (selected) No

6. Provide details of the collection and management of the biospecimen/s.

Saliva will be collected using commercially available test kits for measurement of flow rate, pH, buffering capacity and then cultured for bacterial assessment. The number of teeth, fillings and other retentive sites in mouth influence the bacterial load and a high count of bacteria in dental plaque correlates with salivary bacterial counts, making it possible to assess saliva for cariogenic microbes. Such kits use selective media for mutans streptococci and for lactobacilli.

7. Describe how you will ensure that all biospecimen/s used in this project will be stored securely and describe how you will monitor this as well as the use of the biospecimen/s.

Saliva will be collected using commercially available test kits and will be stored securely at Griffith University

9.7 Research Involving Aboriginal and Torres Strait Islander Peoples

You have indicated that the research involves Aboriginal and/or Torres Strait Islander peoples. You should refer to relevant guidelines as appropriate eg. Values and Ethics: Guidelines for Ethical Conduct in Aboriginal and Torres Strait Islander Health Research http://www.nhmrc.gov.au/publications/synopses/e52syn.htm
National Statement Chapter 4.7 and AIATSIS Guidelines for Ethical Research in Indigenous Studies.

1. What is the estimated proportion of Aboriginal and Torres Strait Islanders peoples in the population from which

participants will be recruited?

Close to 100%

2. Will the Aboriginal or Torres Strait Islander status of participants be recorded?

○ Yes ◉ No

Explain why the Aboriginal or Torres Strait Islander status of participants will be not recorded:
Status will not be recorded as close to 100% of participants will be of Aboriginal or Torres Strait Islander status

3. Will there be or has there been a process of consultation and negotiation between Aboriginal or Torres Strait Islander peoples and the researchers regarding the proposed research?

◉ Yes ○ No

Describe this process of consultation and negotiation.
Include, as appropriate:
- how the consultation process and the research proposal demonstrates the integrity of the researcher,
- negotiation of the aims, anticipated outcomes and priorities of the research,
- consultation regarding community and individual consent to participation in the research,
- the process for negotiating ongoing advice as the research progresses, to monitor ethical standards and minimise unintended consequences,
- how the processes show engagement with the values and processes of participating communities, and
- the process of negotiating access to, and /or control of the results of the research.
The project team has identified the following stakeholders:
The Community Elders
Queensland Health Chief Dental Officer
Cape York Health Council (Apunipima)
Queensland Health Torres and Cape Health and Hospital Service (TCHHS)
Northern Peninsula Area Regional Council (NPARC)
Northern Peninsula Area State College (NPASC).

Professor Johnson visited the community in February 2015, during which face-to-face conversations were held with the above, all of whom expressed their enthusiasm for the project and gave constructive advice as to process.

Since that visit many emails and telephone conversations have been held with the stakeholders. Letters of support are attached.

4. Has there been a role for Aboriginal or Torres Strait Islander peoples in the development of the research and or will there be a role for Aboriginal or Torres Strait Islander peoples in the implementation of the research proposal?

◉ Yes ○ No

Describe the role of Aboriginal or Torres Strait Islander peoples in the development and or implementation of the research.
Include, as appropriate:
- whether any or all of the researchers are Aboriginal or Torres Strait Islander people,
- how Aboriginal or Torres Strait Islander peoples from the community involved in, or affected by, the research have collaborated in the development of the research,
- whether the participating communities have expressed satisfaction with the research agreement, potential benefits and their distribution,
- the extent to which reciprocal obligations, responsibilities and benefits is demonstrated between the researchers and the community.
The oral health project team includes the following members from Aboriginal or Torres Strait Islander descent:
Mrs Valda Wallace (James Cook University)
Prof Yvonne Cadet-James (James Cook University)

Since the project will be conducted simultaneously with the Queensland Health Well Persons' Health Check survey, joint planning is currently being undertaken. Members of the Well Persons' Health Check survey team are of Aboriginal or Torres Strait Islander descent.

5. Describe how the research will provide benefits to the Aboriginal and Torres Strait Islander peoples.

Include, as appropriate
- a description of how the research relates to the health priorities and needs of participant communities,
- a description of benefits for participants and the communities, including establishment and/or enhancement of capacities, opportunities and outcomes beyond the project,
- a description of how the research shows an intent to contribute to the advancement of the health and well being of participants and their communities.

A survey of the dental health of children in this community conducted in 2004 prior to the introduction of water fluoridation in 2005 showed that dental caries was a substantial problem: Caries experience for 6- and 12-year-old children was more than twice the state average and more than four times greater than the average figures for Australian children; the tercile of children with the highest caries experience were 7-8 times the national average; those in the top 10% had mean dmfs (decayed, missing and filled surfaces of teeth in the deciduous dentition) and DMFT (decayed, missing and filled teeth in the permanent dentition) scores more than 10 times that of the rest of Australia. A follow-up oral health survey in NPA conducted in November 2012 by this team, in which we examined over 70% of known resident schoolchildren (n=339), suggests that the dental caries status has improved significantly since the 2004 survey. However, significant unmet treatment needs exist at all ages.

There is an urgent need to reduce the burden of dental decay in these communities, by implementing effective, cost-effective, feasible and sustainable dental prevention programs. Expected outcomes of this study include improved oral and general health of children within the community; an understanding of the costs associated with the intervention provided, and its comparison with the costs of allowing new lesions to develop, with associated treatment costs. The work will benefit Indigenous children and reduce disparities. If found to be effective and cost-effective in reducing dental caries, this initiative could be implemented in similar communities elsewhere.

Over and above this treatment being provided to participants, improved oral and general health of children within the community, an understanding of the costs associated with the intervention provided, and its comparison with the costs of allowing new lesions to develop with associated treatment costs, will benefit Indigenous children and reduce disparities.

10. Declarations And Signatures

Applicant / Principal Researchers (including students where permitted)

Project Title (in full):	Effectiveness, cost-effectiveness and cost-benefit of a single annual professional intervention for the prevention of childhood dental caries in a remote rural Indigenous community
HREC to which this application is made:	
HREC Reference number:	Griffith University HREC

I/we certify that:

- All information is truthful and as complete as possible.
- I/we have had access to and read the National Statement on Ethical Conduct in Research InvolvingHumans.
- The research will be conducted in accordance with the National Statement.
- The research will be conducted in accordance with the ethical and research arrangements of the organisations involved.
- The research will be conducted in accordance with the ethical and research arrangements of the organisations involved.
- I/we have consulted any relevant legislation and regulations, and the research will be conducted in accordance with these.
- I/we will immediately report to the HREC anything which might warrant review of the ethical approval of the proposal (NS 2.37), including:
- serious or unexpected adverse effects on participants;
- proposed changes in the protocol; and
- unforseen events that might affect continued ethical acceptability of the project.

- I/we will inform the HREC, giving reasons, if the research project is discontinued before the expected date of completion (NS 2.38);
- I/we will not continue the research if ethical approval is withdrawn and will comply with any special conditions required by the HREC (NS. 2.45);
- I/we will adhere to the conditions of approval stipulated by the HREC and will cooperate with HREC monitoring requirements. At a minimum annual progress reports and a final report will be provided to the HREC.

Applicant / Chief Researcher(s) / Principal Researcher(s)

Prof Newell Johnson<br>Griffith University	.... Signature	27/3/15. Date
Prof Ratilal Lalloo<br>Griffith University	.... Signature	27/03/2015 Date
Prof Jeroen Kroon<br>Griffith University	.... Signature	27/3/15 Date
Mrs Valda Wallace<br>James Cook University	Valda L Wallace. Signature	27/03/2015. Date
A/Prof Lisa Jamieson<br>The University of Adelaide	.... Signature	27/3/2015 Date
Dr Ohnmar Tut<br>Griffith University	.... Signature	27/03/2015 Date
Dr Sanjeewa Kularatna<br>Griffith University	.... Signature	27/3/2015 Date

Associate Researchers

Prof Paul Scuffham<br>Griffith University	.... Signature	27/3/2015 Date
A/Prof Robyn Boase<br>James Cook University	R. Boase .... Signature	27/3/15 Date
Prof Yvonne Cadet-James<br>James Cook University	.... Signature	27/03/2015 Date
Dr Surani Fernando<br>Griffith University	.... Signature	27/03/2015 Date

Supervisor(s) of student(s)

Project Title (in full):	Effectiveness, cost-effectiveness and cost-benefit of a single annual professional intervention for the prevention of childhood dental caries in a remote rural Indigenous community
HREC to which this application is made:	
HREC Reference number:	Griffith University HREC

I/we certify that:

- I/we will provide appropriate supervision to the student to ensure that the project is undertaken in accordance with the undertakings above;
- I/we will ensure that training is provided necessary to enable the project to be undertaken skilfully and ethically.

Prof Newell Johnson	[signature] Signature	27/3/15. Date
Prof Paul Scuffham	[signature] Signature	27/3/2015 Date
A/Prof Rodney Lea (NO INVOLVEMENT IN STUDY)	NOT APPLICABLE Signature	..../..../........ Date
Dr David Speicher (NO INVOLVEMENT IN STUDY)	NOT APPLICABLE Signature	..../..../........ Date

Heads of departments/schools/research organisation

Project Title (in full):	Effectiveness, cost-effectiveness and cost-benefit of a single annual professional intervention for the prevention of childhood dental caries in a remote rural Indigenous community
HREC to which this application is made:	
HREC Reference number:	Griffith University HREC

I/we certify that:

- I/we are familiar with this project and endorse its undertaking;
- the resources required to undertake this project are available;
- the researchers have the skill and expertise to undertake this project appropriately or will undergo appropriate training as specified in this application.

PROF	DAVID	SITUM
Title	First Name	Surname

Dean Research Health	GRIFFITH UNIVERSITY
Position	Organisation Name
[signature]	2/4/15
Signature	Date

RESEARCH STUDY PROTOCOL

Effectiveness, cost-effectiveness and cost-benefit of a single annual professional intervention for the prevention of childhood dental caries in a remote rural Indigenous community

Investigators:

Chief researcher/investigator:

Professor Newell Johnson (Griffith University)

Principal researchers/investigators:

Professor Ratilal Lalloo (Griffith University)

Professor Jeroen Kroon (Griffith University)

Mrs Valda Wallace (James Cook University)

A/Professor Lisa Jamieson (University of Adelaide)

Dr Ohnmar Tut (Griffith University)

Dr Sanjeewa Kularatna (Griffith University)

Associate researchers/investigators:

Professor Paul Scuffham (Griffith University)

A/Professors Robyn Boase (James Cook University)

Professor Yvonne Cadet-James (James Cook University)

Dr Surani Fernando (Griffith University)

Background:

A 2004 survey in the Northern Peninsula Area (NPA) of Far North QLD, found that dental caries experience of 6- and 12-year-old children was more than twice that of the state average and more than four times greater than the comparable figures for Australian children overall [1]. Soon after this survey the reticulated water supply of the five small rural communities in this area was fluoridated. A follow-up oral health survey in NPA conducted in November 2012 by this team, in which 70% of known resident schoolchildren we examined (n=339), suggests that the dental caries status has improved significantly since the 2004 survey [2]. The fluoridation plant has been out of operation since April 2011 and the likelihood that the water will again be fluoridated is uncertain due to budget constraints and Queensland State Government legislated to give local governments

the power to decide to fluoridate or not. Dental caries rates may again increase in the absence of water fluoridation.

The proposed study will implement a novel dental caries preventive intervention in children to:

(1) reduce the microbial load with the topical disinfectant, povidone iodine [3];

(2) inhibit biofilm adherence to susceptible sites by application of fissure sealants [4]; and

(3) reduce the susceptibility of the tooth to demineralisation by acids generated in the microbial biofilm by the application of a fluoride varnish [5, 6].

While data are available on the effectiveness of the three specific interventions, our novel approach will assess the effectiveness and its cost effectiveness of a less frequent combined application of these interventions.

Research Questions:

The study seeks to:

(1) assess the effectiveness of an annual oral health preventive intervention in slowing the incidence of dental caries in children in a remote, rural Indigenous setting;

(2) identify the mediating role of known risk factors for dental caries; and

(3) assess the cost-effectiveness and cost-benefit of the intervention.

Hypothesis:

We hypothesise that 1 and 2 years after the intervention:

(1) the actual incidence of dental caries in children will be significantly lower than the expected incidence, based on modelling from the two oral health surveys conducted over the past 11 years in the same community, the current survey and

(2) the intervention will be cost-effective and cost beneficial, and therefore feasible and sustainable for broader implementation across similar communities in Australia and internationally.

Aims:

The aim of the study is to reduce the high prevalence of tooth decay in children in a remote, rural Indigenous community in Australia, by application of a single annual dental preventive intervention, to identify the mediating role of known risk factors for dental caries and to assess the cost-effectiveness and cost-benefit of the intervention.

Purpose:

To assess the cost-effectiveness and cost-benefit of the intervention.

Study Design:

This is a longitudinal preventive intervention study. All school children in the NPA will be invited to participate. As it is unethical to withhold any proven intervention from any child, no control group will be created. Children who do not consent to participate may be natural controls if they consent to a dental examination at the end of the study. The actual caries increment in the children who participate will be compared to the expected caries increment modelled on oral health surveys carried out in this community in 2004 (pre-water fluoridation); in 2012 (by the Griffith University team post-partial water fluoridation) and 2015 (baseline survey for this study).

Study Setting:

The study will be conducted in a number of small towns in the remote Northern Peninsula Area (NPA) of Far North Queensland. In the 2011 Census the population of the NPA was estimated at 1,046 and is comprised of 52.8% females and 47.2% males. The median/average age of the NPA population is 22 years, 15 years below the Australian average. 98.7% of people living in these communities were born in Australia.

Study Period:

July 2015 to June 2018.

Informed Consent:

An information sheet will accompany the informed consent form to the parents/guardians of potential participants for approval prior to examination, treatment and any preventive intervention being performed.

Study Subjects:

All children (approximately 600-650) attending the two primary and one secondary school campuses of the NPA State College will be invited to participate in the study. These children will be 6-17 years of age, and almost all are Indigenous.

Data Collection:

Prior to the annual intervention the research team will undertake clinical examination of all consenting school-age children to assess dental caries experience.

The International Caries Detection and Assessment system (ICDAS-II) for clinical caries diagnosis will be used to record caries experience and to determine incidence. This will be measured annually across the 3 years of the study, at baseline and after years 2 and 3 of the project.

General Child Quality of Life and Oral Health-Related Quality of Life (OHRQoL) will be measured at baseline and at years 2 and 3. Existing validated and reliable instruments will be used (CHU-9D [7] and OHIP-14 [8]), appropriately modified for our participants.

Prior to the treatment of existing disease, we will investigate saliva of the participants as a component of caries risk assessment. Saliva will be collected using commercially available test kits for measurement of flow rate, pH, buffering capacity and then cultured for bacterial assessment [9]. The number of teeth, fillings and other retentive sites in mouth influence the bacterial load and a high count of bacteria in dental plaque correlates with salivary bacterial counts, making it possible to assess saliva for cariogenic microbes [10, 11]. Such kits use selective media for mutans streptococci and for lactobacilli.

The retention of the fissure sealants at the follow-up periods will be assessed, and recorded. Saliva of the participants will be collected again in years 2 and 3. Findings will be compared to baseline to assess the impact of the less frequently applied anti-bacterial component of the intervention.

Resources use and costs of providing the intervention will be recorded throughout the intervention period. Resource use and costs to participants to receive the intervention (e.g. time off work to bring the child to the clinic) will be recorded as well as any emergency treatment required between annual visits by the team.

Restorative dental materials required in the treatment of dental caries are approved for use in private practice and the public oral health services.

The following approved materials will be used in the preventive intervention:

Fissure sealants:

Conseal f: http://www.sdi.com.au/en-au/conseal-f/

Fuji VII: http://www.gcasia.info/proddet.asp?prodid=17

Fluoride Varnish:

Colgate Duraphat Single Dose: http://www.colgateprofessional.com.au/products/Colgate-Duraphat-Varnish-Single-Dose/specifics

Povidone (PVP) Iodine:

PDI Povidone-Iodene Swabsticks (1's): http://pdihc.com/all-products/pvp-iodine-prep-pads-and-swabsticks

Data Analysis:

All baseline socio-demographic characteristics will be described for the selected sample using counts and frequencies. Baseline and follow-up caries experience and questionnaire related information will be reported. Dental caries increment (incidence) will be the main outcome measure used to determine the effectiveness of the preventive intervention. The expected caries increment will be modelled from the three oral health surveys conducted in this community (2004; 2012 and 2015) and compared with the actual caries increment from 2015-2016; 2016-2017 and 2015-2017. The mean caries increment will be compared between the expected (modelled) and actual findings, and adjusted for known risk factors for dental caries. The hypothesis will be that caries increment observed in the period of 2015 to 2017 is smaller than the modelled caries increments. Two independent samples t-test will be used for the analysis with significance being determined if $p<0.05$.

Children who receive only a part of the intervention will be separately assessed: for example we will have children who fully participate, those with baseline and only a year 1 follow-up, those with baseline and only a year 2 follow-up. This will 'naturally' further inform us on the most appropriate frequency of this preventive strategy. Both a group and matched analysis will be conducted to account for children who receive only part of the intervention.

A health state transition Markov model will be developed using Tree Age pro software (TreeAge Software Inc., Williamstown, Massachusetts, USA) to analyse the cost effectiveness of the intervention.

The costs of providing the preventive intervention, the costs of all treatment for carious lesions, and the out-of-pocket costs in relation to caries experience will be assessed. These costs will be assigned to each child taking into account the number of surfaces treated. Cost intervention will include sealants, an oral anti-septic application, application of a fluoride varnish and including cost for human resources and logistics. The costs of treating incremental caries will be estimated using government costs for treatments. Total out-of-pocket costs for parents of children with caries will be calculated based on the quantities of resource use provided in the surveys. Mean, median and interquartile range costs will be presented for each major treatment category in caries. All costs will be presented in 2015 AUD.

References:

1. Hopcraft M, Chow W: Dental caries experience in Aboriginal and Torres Strait Islanders in the Northern Peninsula Area, Queensland. Aust Dent J 2007, 52(4):300-304.

2. Johnson NW, et al: Effectiveness of water fluoridation in caries reduction in a remote Indigenous community in Far North Queensland. Aust Dent J 2014, 59(3):366-371.

3. Berkowitz RJ, et al: Adjunctive chemotherapeutic suppression of mutans streptococci in the setting of severe early childhood caries: an exploratory study. J Public Health Dent 2009, 69(3):163-167.

4. Ahovuo-Saloranta A, et al: Sealants for preventing dental decay in the permanent teeth. Cochrane Database Syst Rev 2013, 3:CD001830.

5. Marinho VC, et al: Fluoride varnishes for preventing dental caries in children and adolescents. Cochrane Database Syst Rev 2013, 7:CD002279.

6. Weintraub JA, et al: Fluoride varnish efficacy in preventing early childhood caries. J Dent Res 2006, 85(2):172-176.

7. Stevens KJ: Developing a descriptive system for a new preference-based measure of health-related quality of life for children . Qual Life Res 2009, 18(9):1105-1113.

8. Slade GD, Spencer AJ: Development and evaluation of the Oral Health Impact Profile. Community Dent Hlth 1994, 11(1):3-11.

9. Walsh LJ, Tsang AK: Chair side testing for cariogenic bacteria: current concepts and clinical strategies. JMID 2008, 78(3):143-147.

10. Gabris K, Nagy G, Madlena M, Denes Z, Marton S, Keszthelyi G, Banoczy J: Associations between microbiological and salivary caries activity tests and caries experience in Hungarian adolescents. Caries Res 1999, 33(3):191-195.

11. Ollila PS, Larmas MA: Long-term predictive value of salivary microbial diagnostic tests in children. Eur Arch Paediatr Dent 2008, 9(1):25-30.

Effectiveness, cost-effectiveness and cost-benefit of a single annual professional intervention for the prevention of childhood dental caries in a remote rural Indigenous community

Researcher CV's:

Chief researcher/investigator:

Professor Newell Johnson (Griffith University)

Principal researchers/investigators:

Professor Ratilal Lalloo (Griffith University)

Professor Jeroen Kroon (Griffith University)

Mrs Valda Wallace (James Cook University)

A/Professor Lisa Jamieson (University of Adelaide)

Dr Ohnmar Tut (Griffith University)

Dr Sanjeewa Kularatna (Griffith University)

Associate researchers/investigators:

Professor Paul Scuffham (Griffith University)

A/Professors Robyn Boase (James Cook University)

Professor Yvonne Cadet-James (James Cook University)

Dr Surani Fernando (Griffith University)

Abbreviated curriculum vitae of

Emeritus Professor Newell W Johnson CMG, FMedSci,

MDSc (Melbourne), PhD (Bristol), FDSRCS (England), FRACDS, FRCPAth (UK), FFOP(RCPA), FHEA (UK), FICD

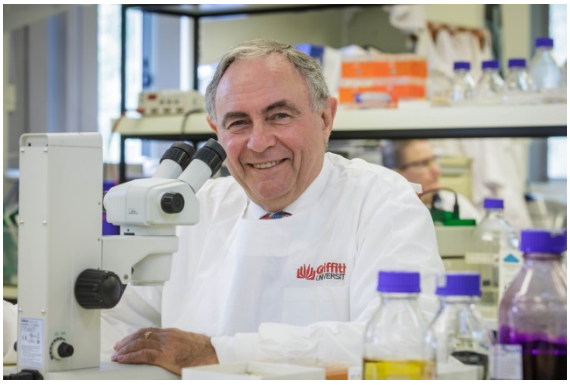

Professor Johnson qualified BDS Melbourne, LDS Victoria in 1960. He worked as a general dental practitioner in country Victoria and as a clinical teacher in dental prosthetics in the University of Melbourne, whilst undertaking a Master's degree by research in the Department of Pathology there. His thesis described tissue reactions to [dental] implant materials.

In 1963 he travelled to the UK where he taught clinical dentistry at University College London and obtained his [dental] surgical Fellowship at the Royal College of Surgeons of England. This was followed by a three year appointment to the Medical Research Council Dental Research Unit in the University of Bristol from which he obtained his PhD on the pathology of dental caries.

In 1968 he was appointed Reader in Experimental Oral Pathology at the London Hospital Medical College [University of London] where he served for 25 years, becoming Professor of Oral Pathology and Head of Department in 1978. During this period he built the largest department of oral pathology in the UK, introduced a Masters' programme in Experimental Oral Pathology, taught undergraduates and supervised a series of PhD students. He, and his department, developed recognition as a centre of excellence in dental education, diagnostic oral pathology, in mucosal biology and diseases, and especially in oral oncology.

He was invited to assume the Nuffield Chair of Dental Sciences at the Royal College of Surgeons of England in 1983 and carried the existing research programme on development of a vaccine against dental caries forward, at the same establishing a programme in oral oncology. Both produced substantial published data.

Concurrent with the Nuffield Chair he was invited to create a new Medical Research Council Dental Research Unit. He led this from 1983 to 1993. This was focussed on the aetiology, pathogenesis and prevention of periodontal diseases. This produced many publications and PhDs. Notably, the Unit was instrumental in moving thinking to the concept of recognising the existence of high risk groups and individuals for oral diseases, and studying methods for their detection.

From 1993 to 2003 he moved to the Chair of Oral Pathology at King's College School of Medicine and Dentistry, University of London. Here was responsible for the diagnostic service in oral pathology, for the clinical service in oral medicine and for undergraduate and postgraduate teaching in these disciplines. The research programme, and the PhDs resulting therefrom, were focussed on oral/head

and neck oncology. During this period he was Director of the World Health Organisation Collaborating Centre for Oral Cancer and Precancer

He was a Consultant in the National Health Service of the United Kingdom between 1968 and 2005 holding appointments with Health Authorities in Bristol, East London, Central London and South London. Concurrent General Dental Practitioner and Specialist Registrations were held with the General Dental Council [UK]. Currently these are held with the Australian Health Practitioner Registration Authority.

He is a former Member of the Global IADR Council and was President of the British Society for Dental Research 2004-2006. He is former President of the British Society for Periodontology and of the section of Odontology of the Royal Society of Medicine, twice. He was Foundation Dean, 2005-2009, School of Dentistry and Oral Health, Griffith University, inaugural Chair of the Australasian Council of Dental Schools and Chair of the Faculty of Oral Pathology of the Royal College of Pathologists of Australasia 2007-2010. He is current Chair of the Queensland Committee of the RACDS.

He is highly cited [h-Index 58; i10-index 205] in head and neck oncology, HIV-disease, cariology, periodontology and oral medicine with over 500 publications and currently leads a team of PhD students and international collaborators from his current base in the Menzies Health Institute Queensland. He was a member of the original Global Oral Health Inequalities Research Agenda [GOHIRA] team and is currently promoting policies for reducing inequalities in risk for oral and oropharyngeal cancer through the IADR, World Health Organisation, FDI World Dental Federation, International Academy of Oral Oncology and governments .

. He is Editor and/or contributor to a number of major textbooks on head and neck cancer and on oral cancer, and to textbooks of periodontology and cariology.

Today, hee leads and/or collaborates in research on epidemiology, risk factors, pathogenesis and prevention of head and neck cancer, of oral aspects of HIV/AIDS and of dental caries and periodontal diseases in most continents; and on studies of dental education. He has trained over twenty Postgraduate Trainees/PhD students from many countries and has projects currently active in India, Bangladesh, Sri Lanka, West Africa and East Africa and is a regular visitor to all continents.

He has received awards for his professional work including: Election as Fellow of the Academy of Medical Sciences [UK]; a Distinguished Scientist Award from the International Association for Dental Research; the Tomes' Medal of the British Dental Association; Membre Etranger of the Academie Dentaire Francais; Academician of the Argentine Academy of Medical Sciences; Award for Research Excellence from Griffith University; and appointment by Her Majesty Queen Elizabeth ll as Companion of the Most Excellent Order of Saint Michael and Saint George for services to public health internationally.

Research publications and other details can be accessed
at **http://www.ncbi.nlm.nih.gov/pubmed/?term=johnson+nw**
Google Scholar researcher profile
BiomedExperts profile
05 April 2015

Recent Curriculum Vitae: Ratilal Lalloo

Home address: 15/16 Mulyan Place, Ashmore, Gold Coast 4214
Mobile: 04 2524 8665
E-mail: Ratilal.Lalloo@gmail.com; rati.lalloo@adelaide.edu.au; r.lalloo@griffith.edu.au
Country of Residence: Australia
Citizenship: Australia and South Africa

Professional Qualifications

1999 – 2002:	PhD in Epidemiology and Dental Public Health, University College London, London, United Kingdom.
1994:	Policy, Planning and Management for Health Sector Reform, Committee on Public Health Education (Western Cape), Sea Point, Cape Town.
1991 - 1994:	Master's (Speciality) in Community Dentistry (M Ch D), University of the Western Cape, Bellville, Cape Town.
1991 - 1992:	Honours Degree in Epidemiology (B Sc Med Hons [Epi]), University of Cape Town, Rondebosch, Cape Town.
1981 - 1986:	Bachelor's Degree in Dentistry (B Ch D), University of the Western Cape, Bellville, Cape Town.

Recent Employment History

Current Positions – April 2015

Chief Investigator B (0.2 appointment), Population Health Program, Menzies Health Institute Queensland, Griffith University, Queensland.

Professor (academic title), School of Dentistry and Oral Health, Griffith University, Queensland.

Adjunct Associate Professor, Australian Research Centre for Population Oral Health, School of Dentistry, The University of Adelaide, Adelaide, South Australia.

April 2014 to March 2015:
Senior Research Fellow, Australian Research Centre for Population Oral Health (ARCPOH), The University of Adelaide, South Australia.
Acting Director, Dental Practice Education Research Unit and The Oral Health Promotion Clearinghouse, Australian Research Centre for Population Oral Health (ARCPOH), The University of Adelaide, South Australia.

January 2009 to January 2014:
Professor and Colgate Chair: Rural, Remote & Indigenous Oral Health, School of Dentistry and Oral Health, Griffith University, Gold Coast, Queensland, Australia – contract expired mid-January 2014.

Recent Publications – in peer review journals

1. Kisely S, Baghaie H, **Lalloo R**, Siskind D, Johnson NW. (2015) A systematic review and meta-analysis of the association between poor oral health and severe mental illness. *Psychosomatic Medicine* 77(1):83-92.
2. Amarasena N, **Lalloo R**. Teething and Sleep Difficulties: findings from the Longitudinal Study of Indigenous children. *ANZ Journal of Public Health* (in press).
3. Kisely S, Baghaie H, **Lalloo R**, Johnson NW. A systematic review and meta-analysis of the association between poor oral health and eating disorders. *British Journal of Psychiatry* (in press).

4. GBD Mortality and Causes of Death Collaborators. (2014) Global, regional, and national levels of age-sex specific all-cause and cause-specific mortality for 240 causes of death, 1990-2013: a systematic analysis for the Global Burden of Disease Study 2013. *Lancet* pii: S0140-6736(14)61682-2. doi: 10.1016/S0140-6736(14)61682-2.
5. **Lalloo R**, Jamieson LM, Ha D, Ellershaw A, Luzzi L. (2014) Does fluoride in the water close the dental caries gap between Indigenous and non-Indigenous children in Australia? *Australian Dental Journal* doi: 10.1111/adj.12239.
6. **Lalloo R**, Kroon J. (2014) Impact of initiatives to reduce public dental waiting lists in Queensland, Australia. *Australian Journal of Primary Health* doi: 10.1071/PY14063.
7. Johnson NW, **Lalloo R**, Kroon J, Fernando S, Tut O. (2014) Effectiveness of water fluoridation in caries reduction in a remote Indigenous community in Far-North Queensland. *Australian Dental Journal* **59**(3): 366-71.
8. Srisilapanan P, Korwanich N, **Lalloo R**. (2014) Associations between social inequality and tooth loss in a household sample of elderly Thai people aged ≥ 60 years old. *Gerodontology* DOI: 10.1111/ger.12140.
9. Ongtengco K, Anthonappa R, Itthagarun A, King NM, **Lalloo R**, Nair, R. (2014) Remineralization of initial enamel carious lesions using fluoridated milk in vitro. *Acta Odontologica Scandinavica* **72**(8): 737-44.
10. Kassebaum NJ, Bertozzi-Villa A, Coggeshall MS … **Lalloo R** … et al. (2014) Global, regional, and national levels and causes of maternal mortality during 1990-2013: a systematic analysis for the Global Burden of Disease Study 2013. *Lancet* **384**(9947): 980-1004.
11. Kumar S, Kroon J, **Lalloo R**. (2014) A systematic review on the impact of parental socio-economic status and home environment characteristics on children's Oral Health Related Quality of Life. *Health and Quality of Life Outcomes* **12**: 41.

From 1994-2013 – Published 58 peer-reviewed publications.

Recent papers delivered (in bold) / co-authored at conferences/courses/workshops

1. **Lalloo R**, Jamieson LM, Ha D, Ellershaw A, Luzzi L. *Does fluoride in the water close the dental caries gap between Indigenous and non-Indigenous children in Australia?* International Indigenous Oral Health Conference, Adelaide, 27th-29th August 2014.
2. **Lalloo R**, Kroon J. *Impact of Funding Incentives on Dental Public Health Waiting Lists.* 92nd General Session & Exhibition of the IADR and Africa/Middle East Regional Meeting, 25th-28th June 2014, Cape Town International Convention Centre, South Africa.
3. Gupta B, Lalloo R, Johnson NW. *Life–course risk factors for cancers of the upper aero–digestive tract in Indian population, special reference to socio–economic determinants and oral health risk factors.* 6th International Meeting: The Dental Biostats Conference. 1st-3rd April 2014. National Wine Centre, Adelaide, Australia.
4. Ha D, Jamieson LM, Luzzi L, Lalloo R, Do L. Trends of caries experience and associated factors among Indigenous children. Annual Scientific Meeting of the ANZ Division of the IADR, 29th Sept-1st Oct 2014, Brisbane, Queensland.

Presented or co-authored more than 50 other conferences from 1995-20013.

Recent Research Grants

2013-2015:

1. Johnson NW, **Lalloo R**, Kroon J, Wallace V, Jamieson LM, Tut O, Kularatna S. *Effectiveness, cost-effectiveness and cost-benefit of a single annual professional intervention for the prevention of childhood dental caries in a remote rural Indigenous community'.* NHMRC Project Grant, 2015-2017; $1,020,590.
2. Gupta B, Johnson NW, **Lalloo R**. *To determine life-course risk factors for cancers of upper-aero digestive tract in India and Australia, with special reference to potential socio-economic determinants and to oral health risk factors.* Population and Social Health Research Program, PhD Researcher Grant, Griffith University; 2013; $3,000.

Abbreviated Curriculum Vitae of
Professor Jeroen Kroon

8 April 2015

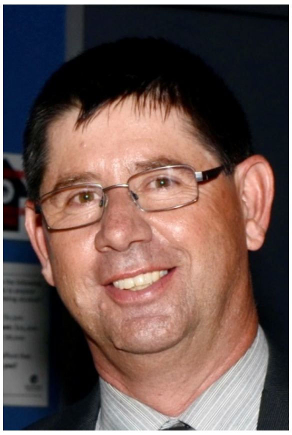

Professional Biography:

After qualifying as a dentist in June 1985, Jeroen was offered a full-time appointment in the Faculty of Dentistry, University of Pretoria, South Africa in July 1987. In 2001 he was appointed as Head of Community Dentistry at the Faculty of Dentistry, University of Limpopo, a position he held until he joined Griffith University in March 2006. His teaching and research interests are in preventive dentistry, epidemiology and public health and he was responsible for the implementation of several outreach projects in South Africa. He was also closely involved in developing new innovative curricula at both schools he was employed at. He obtained a PhD in 2008 from the University of Pretoria, South Africa. His thesis was on "The economic assessment of water fluoridation in South Africa and its impact on human resources and oral health service delivery". Jeroen has served as President of the South African Division and as Regional Board Member of the International Association for Dental Research. At Griffith University he has taken a leading role in curriculum development. He prides himself in his teaching and was awarded a Griffith Award for Excellence in Teaching in 2008 and a Highly Commended award in 2012. He is a member of the Australia and New Zealand Division of IADR.

Present appointments:

- Professor in Public Health, Community and Preventative Dentistry: March 2006 - Present
- Deputy Head of School: January 2008 – February 2011; May 2014 - Present

Qualifications:

- Bachelor of Dentistry (BChD) - 1985
- Bachelor of Dentistry (Honnours) - 1987
- Postgraduate Diploma in Health Administration (Dentistry) - 1990
- Master of Dentistry (Community Dentistry) (MChD) - 1992
- Postgraduate Diploma in Public Health (Dentistry) - 1993
- Diploma in Tertiary Education - 1994
- Philosophiae Doctor (PhD) - 2008
- Fellow of the International College of Dentists (Australasian Section) – 2011

Professional memberships:

- Australia and New Zealand Division of the International Association for Dental Research

Research interest and research expertise:

- Preventive dentistry
- Fluoride
- Mobile health care systems
- Human resources planning
- Oral health systems
- Educational research

Articles published in accredited scientific journals (n=23) - only articles published in the last 5 years are listed below:

1. **KROON, J.** & VAN WYK, P.J. 2012. A retrospective view on the viability of water fluoridation in South Africa to prevent dental caries. *Community Dentistry and Oral Epidemiology,* 40 (5), p. 441-450.
2. **KROON, J.** & VAN WYK, P.J. 2012. A model to determine the economic viability of water fluoridation. *Journal of Public Health Dentistry,* 72 (4), p. 327-333.
3. LALLOO, R. & **KROON, J.** 2013. Analysis of public dental service waiting lists in Queensland. *Australian Journal Of Primary Health*. http://dx.doi.org/10.1071/PY13048
4. **KROON, J.**, REID, K. E., CUTTING, J. R., LALLOO, R. & CHIU, K. C. 2014. Opinion of residents from the Gold Coast, Queensland, on community water fluoridation. *Journal Of Investigative And Clinical Dentistry,* 5 (1)**,** p. 58-64.
5. KUMAR, S., **KROON, J.** & LALLOO, R. 2014. A systematic review of the impact of parental socio-economic status and home environment characteristics on children's Oral Health Related Quality of Life. *Health and Quality of Life Outcomes,* 12 (41), p. 1-15.
6. JOHNSON, N.W., LALLOO, R., **KROON, J.**, FERNANDO, S. & TUT, O. 2014. Effectiveness of water fluoridation in caries reduction in a remote Indigenous community in Far-North Queensland. *Australian Dental Journal*, 59, p. 366-371.
7. LALLOO, R. & **KROON, J.** 2014. Impact of initiatives to reduce public dental waiting lists in Queensland, Australia. *Australian Journal of Primary Health.* DOI: 10.1071/PY14063
8. STORRS, M., ALEXANDER, H., SUN, J., **KROON, J.** & EVANS, J. 2015. Measuring Teams-Based Interprofessional Education Outcomes in Clinical Dentistry: Psychometric Evaluation of a New Scale. *Journal of Dental Education,* 79 (3), p. 249-258*.*
9. KROON, J., COX, J.A., KNIGHT, J.E. NEVINS, P.N. & KONG, W.W. Mouthguard use and awareness of junior rugby league players in the Gold Coast, Australia. *Journal of Science and Medicine in Sport.* **(accepted for publication)**

Current research grants:

1. Economic Assessment of Water Fluoridation in South East Queensland and the Northern Peninsula Area of Far North Queensland. Griffith University, Griffith Health Institute Population & Social Health Research Program, Early Career Researcher Grant Scheme; 2012. $5,000.
2. Effectiveness, cost-effectiveness and cost-benefit of a single annual professional intervention for the prevention of childhood dental caries in a remote rural Indigenous community. NHMRC Application: APP1081320 for funding in 2015. $1.1 million.

Valda Wallace

Curriculum Vitae Outline

2015

Personal Details

Name:	Valda Wallace
Phone Nos:	(07) 42321041 work<br>0408211046 mobile
Email Address:	valda.wallace@jcu.edu.au
Nationality:	Australian<br>Aboriginal Language Group – Gugu Badhun People of the Valley of Lagoons, north Queensland

Qualifications

- Master of Indigenous Studies
  James Cook University
- Bachelor of Teaching (Adult/Vocational)
  Northern Territory University
- Associate Diploma of Education (Adult/Vocational)
  Northern Territory University

Professional Membership

Recent:

- Lateral Violence Steering Committee/Working Group -2008-2012.
- Wuchopperen Health Service - Cairns and regional north Qld – Board Member 2003 - 2009
- Aborigines and Islanders Alcohol Relief Service – Cairns and Atherton Tablelands – Board Member 2005 - 2010

James Cook University Boards and Committees:

- Human Ethics Monitor for research involving Indigenous people/matters
- Member Human Ethics Committee
- Education Committee Faculty Representative – March 2010 – February 2012
- Faculty Board of Studies Committee 2010-2014
- Faculty Teaching and Learning Committee 2008-2014

Community Involvement

Current:

- Gugu Badhun Aboriginal Group (family language group)
- Member – Girringun Aboriginal Elders Reference Group (Body representing 9 Traditional Owner Groups in the Cardwell Wet Tropics and inland Dry Tropics region).

Employment History

**January 2003 to current date:**	Lecturer<br>Deputy Head 2003-2014.<br>School of Indigenous Australian Studies now Australian Indigenous Studies, Office of the Senior Deputy Vice Chancellor.<br>James Cook University
**July 2002 – January 2003:**	Coordinator – Cairns Indigenous Youth Outreach Program<br>Department of Families.
**July 1999 –July 2002:**	Indigenous Liaison Officer<br>Indigenous Policing Development Unit<br>Northern Territory Police Service
**April 1992 – July 1999:**	Police Auxiliary Officer<br>Northern Territory Police Service

Current Grants

- Chief Investigator – "Effectiveness, cost-effectiveness and cost-benefit of a single annual professional intervention for the prevention of childhood dental caries in a remote rural Indigenous community". NH&MRC Grant 2014
- Chief Investigator – "Gauging the value of Flexible Learning Options for disenfranchised youth and the Australian community" ARC Linkage Grant 2013

Recent Publications

Graham DG, Wallace VL, Selway DA, Howe EC and Kelly AT (2014) *Why are so many Indigenous women homeless in Far North and North West Queensland, Australia? Service providers' views of causes*. Journal of Tropical Psychology, 4. pp. 1-9

Wallace VL, Graham DG, Selway DA and Kelly AT (2014) *Their story: homeless Indigenous women in Cairns and Mount Isa, Australia*. Journal of Tropical Psychology, 4. pp. 1-11

Two page CV; Lisa Jamieson

Top 5 publications in last 5 years

1. Kapellas K, Maple-Brown LJ, **Jamieson LM** et al. Effect of periodontal therapy on arterial structure and function among Aboriginal Australians: a randomized, controlled trial. Hypertension. 2014;64:702-708. An important contribution to the literature, demonstrating that comprehensive periodontal therapy can improve cardiovascular health among Aboriginal Australians. Hypertension has an IF of 7.6, with the number of dental-related papers in this journal being very few.
2. Jones K et al., **Jamieson LM**. Development and psychometric validation of a Health Literacy in Dentistry scale (HeLD). Community Dent Health. 2014;31:37-43.An instrument that captures functional oral health literacy and which is acceptable for use among vulnerable populations is in demand. This paper has drawn enormous international interest from those working in the field.
3. Kapellas K et al., **Jamieson LM**. Effects of full-mouth scaling on the periodontal health of Indigenous Australians: a randomized controlled trial. J Clin Periodontol. 2013;40:1016-1024. Periodontal therapy improved periodontal health in the absence of tooth brushing; demonstrating periodontal therapy is a pragmatic means of improving periodontal health in this vulnerable group.
4. **Jamieson LM** et al. Substance use and periodontal disease among Australian Aboriginal young adults. Addiction. 2010;105:719-726. Generated marked media interest, as it was the first time that substance use had been linked with periodontal disease in an Australian Aboriginal population, and the first time that petrol sniffing had been linked with periodontal disease in any population.
5. Parker EJ, **Jamieson LM** et al. The oral health of Indigenous children: a review of four nations. *J Paed Child Health*. 2010;46:483-486. Invited review from a journal with a strong reputation in child public health. There was a special interest to focus on Indigenous children at an international level, hence the collaboration with investigators in New Zealand, Canada and the US.

Overall Track Record in the last 5 years

CI Jamieson is a highly regarded Indigenous oral health researcher, being invited to take part in symposia at international meetings at an exponential level over the last 5 years (2 invitations for 2015 thus far). In 2011 she established the University of Adelaide’s Indigenous Oral Health Unit (www.arcpoh.adelaide.edu.au/iohu/); the 1st in Australia and 1 of 2 in the world. In the last 5 years she has organised national workshops, international conferences and co-ordinated 3 special issues of journals relating to Indigenous oral health. The number of Indigenous staff in her Unit has doubled every year since 2011 (currently 7), and she is actively sought as a higher degree research supervisor from Aboriginal and non-Aboriginal students at state, national and international levels. Lisa has published 121 papers, (61 in the last 5 years), 82 related to Indigenous oral health (26 in last five years), 53 as 1st author (17 in last 5 years) and 45 as senior (last) author (25 in last five years). In the past 5 years she has presented on Indigenous oral health at 13 international conferences, 4 invited.

In the last 5 years, CI Jamieson has been CIA on 4 NHMRC project grants; all randomised controlled trials. The PerioCardio study (APP627100; $1.3M over 3 years) was an intervention involving periodontal treatment to Aboriginal adults in the NT in a bid to reduce risk of cardiovascular events. Fifteen publications have resulted, including a number in high impact cardiovascular journals (for example, Hypertension). We employed 6 Aboriginal staff and have 1 completed PhD. The Oral Health Literacy study (APP627101; $0.6M over 3 years) was an intervention among Aboriginal adults in a regional South Australia location that aimed to increase knowledge around context-specific barriers, such as access to local publicly-funded dental services, that impact oral health. Four Aboriginal staff were employed, 5 papers have been published and 1 PhD student is currently writing 4 additional papers. The Baby Teeth Talk study (APP627350; $1.4M over 5 years) is an international collaboration that aims to determine if a behavioural intervention comprising 4 components prevents early childhood caries among Indigenous children in Australia, New Zealand and Canada. There are currently 9 papers published, 1 PhD student working on an additional 4 papers and 6 Aboriginal staff employed. The Perio-CKD study (APP1078077; $1M over 4 years) is another periodontal intervention, this time among Aboriginal

adults residing in Central Australia with chronic kidney disease. We will be employing two Aboriginal research officers, one project manager and have one PhD student about to enrol. CI Jamieson has additionally, in the last 5 years, been CIB (APP1087986, Personalised Medicine, $1M over 3 year) and CID (APP1081320, early childhood caries intervention, $1M over 3 years) on other NHMRC project grants involving Aboriginal Australians.

Since 2005, CI Jamieson has been an independently-funded researcher; NHMRC Early Career Fellowship followed by 2x Career Development Fellowships. She is currently President of the International Association of Dental Research's Behavioural, Epidemiologic, Health Services Research scientific group and convenor of the University of Adelaide's Animal Ethics Committee. She has sat on 3 NHMRC GRPs in the last 5 years and regularly reviews for other funding bodies. CI Jamieson is on the Editorial Board of BMC Public Health and is a reviewer for 16 international journals, among them the American Journal of Public Health.

RESEARCH AND SCHOLARSHIP IN PAST FIVE YEARS:

Establishing an Indigenous Oral Health Unit within the University of Adelaide.

As the Unit's Founding Director, much of Lisa's activity has focused on fostering collaborative relationships that allow the Unit's research to have a direct impact on Indigenous oral health-related policies, and on creating the research output most likely to reach our target audiences. Running a Unit and establishing its credibility requires ongoing consultation with experts in the field, negotiating funding agreements, determining staffing and outputs, policy advice, governance structures and briefings. We hope to establish a reputation as one of the best centres for Indigenous oral health in the world; with key mandates being research, education and advocacy.

Collaborative research activity

Lisa is a strong advocate of collaborative research, which is vital in the field of Indigenous oral health. Current collaborators include: Menzies School of Health Research, University of Otago, University of Toronto, University of Colorado, University of North Carolina, University of Sydney, Baker IDI Heart and Diabetes Institute, University of South Australia, University of Maryland, University of New Mexico, Flinders University, Edith Cowan University, South Australian Health and Medical Research Institute and the University of Melbourne's Indigenous Eye Health Unit.

PROFESSIONAL ACTIVITY, INCLUDING SERVICES TO COMMUNITY

International, national and state

- President; Behavioural, Epidemiological, Health Science Research Group of the IADR
- Editorial board member of BMC Public Health, reviewer for 16 international journals.
- Reviewer for New Zealand's Health Research Council Funding applications
- Member of NHMRC's Indigenous GRP, Population Health GRP and external reviewer
- Substantial media involvement, with 2014 including a televised interview for an Indigenous oral health documentary, lead article in 'Bite' magazine, article in The Australian newspaper
- Consultancy services for the Australian Indigenous Health Infonet.
- Finalist in the 2010 South Australian Science Excellence in Public Good awards
- Co-ordinator, Aboriginal reference groups for two SA-based oral health research projects.
- Board member and on monthly volunteer roster for homeless soup kitchen (organised through the Mary Magdalene Centre).

SUMMARY: In the last 5 years Lisa has demonstrated academic excellence and outstanding contribution to research in the field of Indigenous oral health. She has been successful in obtaining competitive international and national grant funding; secured a large proportion of her own salary; published extensively; currently manages 24 staff, PhD and Honours students; has organised symposia and special journal issues for international conferences, been invited to present at international conferences and invited to collaborate with colleagues at many levels.

RESUME

Ohnmar K. Tut BDS, MPhil

Education: 1968 – 1974 Bachelor of Dental Surgery, Institute of Dental Medicine, Yangon, Myanmar
2006 - 2009 Master of Philosophy, Higher Degree Research (Dentistry), University of Queensland, Australia
2005 – 2007 Maternal and Child Health Leadership Summer Program, University of Hawaii, John A. Burns School of Medicine, Honolulu, U.S.A.

Awards and Honors

International Association of Dental Research, Aubrey Sheiham Award for Distinguished Research in Dental Public Health Sciences, 2010

Employment History

2012 – to date Adjunct Senior Research Fellow, Griffith Health Institute, Griffith University, Australia
2011 – to date Oral Health Consultant, US Affiliated Pacific Islands Jurisdictions
2007 - 2011 Project Director, TOHSS, RMI
2001 to 2010 Chief of Dental Preventive Services, Republic of Marshall Islands
2004 to date - Faculty and Adjunct Instructor, Oral Health Sciences Department, University of Washington
2006 to 2009 - Honorary Lecturer, Dental School, Otago University, New Zealand
1997 to 1999 – Dentist, Pohnpei State Hospital, Federated States of Micronesia
1993 to 1996 – Dentist, Nauru General Hospital, Republic of Nauru
1984 to 1992 – Clinical Demonstrator, Department of Children's Dentistry and Orthodontics, Institute of Dental Medicine, Myanmar
1975 to 1984 – Dentist, Directorate of Health, Myanmar
1974 to 1975 – Internship, Yangon General Hospital and Institute of Dental Medicine, Myanmar

Consulting for Government, Peer Review Panels:

2005 World Health Organization Short Term Oral Health Consultant and Director, In-service Training Course for the Department of Public Health, Commonwealth of the Northern Mariana Islands
2008 – to date Objective Grant Reviewer, Health Resources and Services Administration (HRSA), U.S.A.

Teaching and Training Experience in the Pacific Region:

2006 - to date Regional Oral Health cross-disciplinary trainer for US Affiliated Pacific Islands.
2005 Director and Principal Trainer, 15-week Full-time Dental Assistant Training Program, Ministry of Health, Republic of Marshall Islands
2003 Co-trainer, Health Assistant Training Program, Ministry of Health, Republic of Marshall Islands

Research:

Project Director, Pacific Islands Early Childhood Caries Prevention Project AC F, Head Start Innovation and Improvement Projects, Department of Health and Human Services, PI Milgrom, P.
Local Project Director and Clinical Scientist, 'Xylitol for the Prevention of Acute Otitis Media and Early Childhood Dental Caries' MCHRB, HSRA. PI Milgrom, P
Project Director, The effect of periodontal treatment and the use of a triclosan containing dentifrice on glycaemic control in Diabetics, The University of Queensland, Australia. PI Seymour, G

Grant Support:

9/01/03 - 8/31/07 Oral Health Promotion in the Marshall Islands State Oral Health Collaborative Systems Grant, U.S. HRSA, MCHB. Role: Wrote grant application and served as PI.
7/01/05 - 6/30/08 Xylitol for Acute Otitis Media & Early Childhood Caries (Randomized Trial). HRSA Maternal and Child Health Research Bureau. Role: Served as local on-site director, clinical examiner, and Principal Investigator (PI) of subcontract to RMI.
9/01/05 – 8/31/08 Pacific Islands Early Childhood Caries Prevention Project. Administration for Children and Families, HRSA. Role: Project Director and PI of subcontract to RMI.
9/01/07 - 8/31/08 Planning Phase Grant, Development of Regional Dental Paraprofessional Training Program US HRSA. Role: Conducted feasibility study for PIHOA.
9/01/07 - 8/31/11 Development of Model Training Site and Delivery of Oral Health Prevention for Well babies and Children with Special Health Care Needs, Targeted Maternal and Child Oral Health Care Service Systems Grant, U.S. HRSA. Role: Wrote grant application and served as PI, RMI
9/01/15 – 8/31/18 Oral Health Workforce Activities Grant, U.S. HRSA. Role: Wrote grant application and serve as Program Consultant Investigator, FSM

Publications:

1. NW Johnson, R Lalloo, J Kroon, S Fernando, O Tut, 'Effectiveness of water fluoridation in caries reduction in a remote Indigenous community in Far North Queensland' Australian Dental Journal 2014; 59: 1–6
2. Chi Donald, Tut Ohnmar, Milgrom Peter, "Cluster-randomized Xylitol Toothpaste Trial for Early Childhood Caries Prevention", Journal of Dentistry for Children (JDCHILD-2012-10-1107) Jan-April, 2014
3. Milgrom, P., Tut, Ohnmar K., Gilmatam, J., Gallen, M., & Chi, D.L. Areca use among adolescents in Yap and Pohnpei, the Federated States of Micronesia. Harm Reduction Journal, *10*, 26, 2013
4. Peter M Milgrom DDS, Ohnmar K Tut BDS MPhil, Lloyd Mancl PhD "Topical Iodine And Fluoride Varnish Effectiveness In The Primary Dentition: A Quasi-Experimental Study". Journal of Dentistry for Children 78:3, 2011
5. Tut OK. Milgrom P, "Topical Iodine And Fluoride Varnish Combined Is More Effective Than Fluoride Varnish Alone For Protecting Erupting First Permanent Molars: A Retrospective Cohort Study". J Public Health Dent, 2010 Summer;70(3):249-52
6. Milgrom P, Ly K, Tut O, et al. 'Xylitol Pediatric Topical Oral Syrup to Prevent Dental Caries'. Arch Pediatr Adolesc Med/Vol 163 (7) 2009 July
7. Milgrom P, Tut OK. Evaluation of Pacific Islands Early Childhood Caries Prevention Project: Republic of the Marshall Islands. J Public Health Dent. 2009 Mar 31
8. Milgrom P, Weinstein P, Huebner C, Graves J, Tut O. 'Empowering Head Start to improve access to good oral health for children from low income families'. J Maternal and Child Health 2008 Feb
9. Tut OK, Langidrik JR, Milgrom PM, 'Dental manpower development in the Pacific: Case study in the Republic of the Marshall Islands'. Pacific Health Dialog 2007, 'Developing Human Resources for Health in the Pacific' ;14(1)
10. Tut OK, Lefagopal C, Milgrom PM. 'Brief report on oral health prevention training in Yap State, Federated States of Micronesia'. Pacific Health Dialog 2007 'Developing Human Resources for Health in the Pacific';14(1)
11. Tut OK, Greer M.H.K, Milgrom P, 'Republic of the Marshall Islands: Planning and Implementation of a dental caries prevention program for an island nation'. Pacific Health Dialog, J Community Health Clin Med Pacific. 2005 March; 12(1)

Book Chapter

12. Milgrom P, Tut O, Chi D, Draye M, Acker M. Oral Health, Chapter 33 in Burns et al. Pediatric Primary Care 4th edition Handbook for Nurse Practitioners. St. Louis: Elsevier, 2008 - 5th edition issued in 2011, 6th edition in 2015

Unpublished Masters Thesis:

13. The effect of periodontal treatment and the use of a triclosan containing dentifrice on glycaemic control in Diabetics, 2009

Presentations

1. Milgrom P, Tut OK, Mancl LA. 'Combined Therapy For Tooth Decay With PVP-Iodine And Fluoride Varnish', International Association of Dental Research (IADR), Barcelona, Spain, July 2010. Poster #137968
2. Milgrom P, Tut O, Berkowitz R, 'New Technology for Prevention of Tooth Decay - Combining New Agents with Fluoride' National Oral Health Conference, Portland, Oregon, USA, April 2010
3. Tut OK, Briand K, Milgrom P. 'Integration of Primary Dental Prevention into Well-baby Program: Marshall Islands' World Congress on Preventive Dentistry/IADR, Phuket, Thailand, Sept 2009. Poster #124373
4. Milgrom P, Tut O, Ly K, et al. 'Xylitol Topical Oral Syrup Prevents Early Childhood Caries: A RCT'. Poster presented at IADR, Toronto, Spring 2008. #19722
5. Milgrom P, Huebner C, Tut O. 'Empowering Head Start to improve access to good oral health', Nat. Head Start Association 12th Annual Birth to Three Institute, Washington DC August, 2008
6. Milgrom P, Tut O. 'Empowering parents to improve access to good oral health', Nat Head Start Association, 24th Annual Parent Training Conference, Indianapolis, December, 2007
7. Graves J, Milgrom P, Tut O. 'Development of a sustainable and prevention-oriented oral health intervention for early childhood programs in the Pacific Islands'. APHA, Washington, D.C., Fall 2007.Poster #160787
8. Tut O, Ly K, Heima M, Milgrom P. 'Pilot testing xylitol snack intervention in Head Start': Baseline Data. IADR, Brisbane, Spring 2006. Poster #0153
9. Tut O, Milgrom P. 'Prevalence of Early Childhood Caries in Majuro, RMI' National Oral Health Conference, Boston, MA, 2002

Sanjeewa Kularatna CV

Current Position Research Fellow, Centre for Applied Health Economics, School of Medicine, Griffith University;

Contact Details School of Medicine, Logan Campus, Griffith University, University Drive, Meadowbrook, Queensland 4131;Phone: +61 (0) 733821567 / +61450574376;Fax: +61 (0) 33821338;Email: s.kularatna@griffith.edu.au

Academic Qualifications

Degree	*Institution*	*Field*	*Year*
PhD(under examination)	Griffith University	Health Economics	2014
Doctorof Medicine	University of Colombo	Public health/ Health economics	2010
Master of Science	University of Colombo	Community Dentistry	2005
Bachelor of Dental Surgery	University of Peradeniya	Dental Surgery	2001

Academic Awards

Griffith University Vice Chancellors Award for best research group 2012

President's Award for scientific Publication January 2014. Colombo. Sri Lanka

Professional Registration; Sri Lanka Medical Association (Registration number 1744)

Memberships

International Health Economic Association (IHEA)

ISPOR Australia Chapter / Asia Pacific

College of Community Dentistry Sri Lanka

Sri Lanka Dental Association

Publication and reports

Refereed Journal Papers

1. Sanjeewa Kularatna, Jennifer A Whitty, Newell W Johnson, Ruwan Jayasinghe. Paul A. Scuffham. EQ-5D derived population norms for Sri Lanka. PlosOne November 03.2014.DOI: 10.1371/journal.pone.0108434
2. Sanjeewa Kularatna, Jennifer A Whitty, Newell W Johnson, Ruwan Jayasinghe. Paul A. Scuffham. Valuing EQ-5D Health Statesfor Sri Lanka. Quality of Life Research. 2014 Dec 28. [Epub ahead of print]

3. Sanjeewa Kularatna, Jennifer A Whitty, Newell W Johnson, Ruwan Jayasinghe. Paul A. Scuffham.Development of an EORTC-8D utility algorithm for Sri Lanka. Medical Decision Making. November 17 2014. DOI: 10.1371/journal.pone.0108434
4. Sanjeewa Kularatna, Jennifer A Whitty, Newell W Johnson, Paul A. Scuffham. Study protocol for valuing EQ-5D-3L and EORTC-8D health states in a representative population sample in Sri Lanka. Health and quality of life outcomes 2013; 11(1).149
5. Sanjeewa Kularatna, Jennifer A Whitty, Newell W Johnson, Paul A. Scuffham. Health State Valuation in Low and Middle Income Countries: A Systematic Review of the Literature. Value in Health 2103; 16(6), 1091-1099
6. Sanjeewa Kularatna, Joshua Byrnes, Paul A Scuffham. Study protocol: Comparison of inconsistency between Time Trade Off and Discrete Choice Experiments in EQ-5D- 3 L health state valuations.Journal of health Economics and Outcomes Research, 2(1) p87-95
7. Margaret Pukallus, Kathryn Plonka, Sanjeewa Kularatna, Louisa Gordon, Adrian G Barnett, Laurence Walsh, W. Kim Seow. Cost-effectiveness of a telephone-delivered education program to prevent early childhood caries in a disadvantaged area: A cohort study: BMJ open 3 (5).
8. Kularatna S, Whitty JA, Scuffham PA. Comments on the use of vignettes and the EQ-5D to value disease specific health states. Value in Health 2012; 15: 591-592
9. Kumarihami SL, Subasinghe LD, Jayasekara P, Kularatna SM, Palipana PD, The prevalence of early childhood caries in 1-2 years olds in a semi-urban area of Sri Lanka. BMC research notes, ISSN: 1756-0500, 2011 Sep 09; 04: 336
10. SB Agampodi, MSM Kularathna, PMRBI Pathiraja. Intra-rater and inter-rater reliability of hemoglobin colour scale method. Indian Journal of Community medicine. 2009;34(4):367-368
11. Kularatna S. Ekanayake L. Root surface caries on older individuals for Sri Lanka. Caries Research.2007;41:252-256
12. Rongzhen Koh, Margaret Pukallus, Sanjeewa Kularatna, Louisa G Gordon, Adrian G Barnett, and W. Kim Seow. Cost-effectiveness of home visits compared with telephone contacts to prevent early childhood caries. Submitted to Community Dentistry and Oral Epidemiology. June 2014

Professor Paul A. Scuffham, BA (Otago), PhD (Otago)

Current Clinical and/or Academic Roles and Affiliations
2006-current: Professor, Health Economics, School of Medicine, Griffith University
2011-current: Director, Population of Health Research Program, Griffith Health Institute, Griffith University

Current and Previous Leadership Roles
In 2006, I moved to Griffith University and established the Centre for Applied Health Economics. This centre is now one of the 3 largest health economics groups in Australia, employing more than 20 staff with an income exceeding $2 million annual in competitive research funds. I have major contracts and lead a team of health economists to provide top level technical advice to the Australian Department of Health through the Pharmaceutical Benefits Advisory Committee and the Medical Services Advisory Committee, and to state health departments through HealthPACT.

In 2011 I took on the role of Director of the Population & Social Health Research program at Griffith. This research program has 84 academic staff and 88 PhD students from a range of clinical and social science disciplines. My role is to develop processes to facilitate high quality research in this area.

My leadership at the local level includes founding member of the Queensland Policy Advisory Committee on new Technology (QPACT) and the Clinical Redesign and Innovation Board (CRIB) – both are top level committees in Queensland Health. I have lead the development of decision-making processes to include assessment of the clinical and the economic evidence (including developing the economic evidence where this is absent) for both of these committees. This has provided a framework for assessing the potential return on investment from change in clinical practice and from introducing new technologies, as well as for monitoring and evaluating these changes during implementation.

Nationally, I established the Australian Chapter of the International Society for Pharmacoeconomics and Outcomes Research (ISPOR) and was elected as President for 3 years (2011-13) before stepping down. This society brings together academics, government and industry with a focus on providing education around health economics and health outcomes. The society is now fully operational and runs several workshops and meetings annually. I am on the review board for the National Heart Foundation, and a member of the Assigners Academy of the NHMRC.

Internationally, I am Co-Editor of Value in Health and Associate Editor of Medical Decision Making. These journals are leading journals in the field of health care sciences and services, and health policy and services.

Major Awards and Prizes
- Prize for Best Research Paper, American Heart Association – Cardiovascular Nursing Council (Dallas, 2013): Stewart S, …, Scuffham PA. Impact of home versus clinic based management of chronic heart failure: the Which Heart failure Intervention is most Cost-effective & consumer friendly in reducing Hospital care (WHICH?) multicentre, randomized trial. Journal of the American College of Cardiology 2012 2;60(14):1239-48.
- Award for Excellence of a Research Group ($10,000), PVC (Health) Awards 2011, Griffith University
- Academic Research Award, Academic and Clinical Excellence Awards, Griffith University, 2010 & 2011
- Outstanding Paper Award, Literati Network Awards for Excellence, 2009
- NHMRC Career Development Award, 2005-2008
- Health Research Council of New Zealand, Postdoctoral Research Fellowship, 1998-2000
- Donald Reid Economics Research Award, 1997

Research Outputs
- ***Competitive research funding***

Since 2004, I have obtained more than $21million in nationally competitive grants, with $9m as Principal Investigator and $12m as Chief Investigator, including a NHMRC Program grant. In addition, I am an Associate Investigator on another 7 grants ($9m). Funding from industry partners is an important aspect of my research that ensures there is an avenue to implement research findings. I have obtained industry funding from various sources including Bupa Australia, Edwards LifeSciences, the Department of Health and Aging, South Australia Health and Queensland Health.

- ***Publications***

I have published over 200 articles in peer reviewed journals, economics discussion papers and commissioned reports. In

the last 5 years I have published >80 original research articles (lead or senior author on 45%), and prepared 8 major reports to governments (lead author of 3 and senior author of 5). My *h*-index is 32 with more than 3500 citations; 21 of these articles have more than 50 citations.

- ***Mentoring and Training***

I have developed a core staff of 22, including 14 health economists and an Assoc Prof in statistics. My team of health economists includes 12 with PhDs of which 3 have progressed to Senior Research Fellow. Others I have mentored have moved on to take up positions such as Assoc Prof in Health Economics at UQ. Through PSHRP I mentor 3 Professors and 2 Assoc Professors. Currently supervise 5 PhD students and have supervised 7 Masters students and 6 PhD students to completion.

Research Translation

A fundamental part of my research, and development of implementation science, is getting research into practice through QPACT and CRIB in Queensland Health. Having played a lead role in developing the processes for these committees, I am encouraged to see many "field trials" of technologies and healthcare services being tested for feasibility and requirements for adaptation to Australia. These field trials require an evidence base (i.e. moving beyond establishing efficacy) and focus on additional needs for implementation such as training of staff and increases/reduction in use of other services. A key component of determining which interventions to test and of evaluating these trials is estimating of the return on investment; I have lead the development of the tools to calculate this for clinical/senior administrative staff in Queensland Health. To date we (QPACT and CRIB) have funded 19 implementation trials costing $25m.

My role in HealthPACT has a direct influence on healthcare in Australia. For example, following the results of a recently reported clinical trial, we issued a notice to all State Health departments advising them not to undertake renal denervation. Other examples are include the national workshop on the use of Transcatheter Aortic Valve Implantation (TAVI), information on screening for atrial fibrillation, MRI screening for prostate cancer, endobronchial valves for emphysematous bullae, and numerous other interventions.

Moreover, my reviews commissioned by the Australian Department of Health for the PBAC on statins, biologics for rheumatoid arthritis and triple therapy for diabetes has resulted in over $1bn of cost-savings to the PBS. These reviews extend from the molecular action, indicated population, synthesis of the clinical evidence using indirect comparisons and network analyses, estimates of the value for money, the cost to the PBS and a myriad of scenarios of changes to the clinical algorithm with estimates of the effect on the healthcare budget, have determined the most efficient use of these classes of medications. The key scenarios are presented to the PBAC and recommendations are made to the Minister. The review of biologics resulted in major changes to the clinical algorithm (i.e. choice of 1st-line, 2nd-line etc therapy) and removal of ineffective medications from the PBS which save the PBS $600m over the forward estimates (4 years); and the review of statins resulted in immediate cost reductions in Atorvastatin and Rosuvastatin of 25% with planned reductions to maintain a 12.5% relativity to the price of Simvastatin. This is expected to save the PBS more than $800m over forward estimates.

My most recent research – engaging the public in healthcare policy making – funded by an ARC Linkage grant, has seen Citizen Juries become routinely used in the Metro South HHS, Queensland. This study is not yet complete but this approach, compared with other methods for engaging the public, has proven highly useful for informing health policy on difficult matters, such as priority setting for bariatric surgery. I expect approach this will become widely used once the study has finished and the dissemination of results commenced.

Curriculum Vitae

Robyn Margaret Boase BDS, BSc, FRACDS.

**Address**	: 12 / 18 Janett St, Yorkeys Knob, Queensland, 4878
**Phone**	: H (07) 40810 868 : W (07) 42321295: M 0409392816
**Postal Address**	Home : As Above
	Work : College of Medicine and Dentistry, James Cook University, PO Box 6811, Cairns, Qld, 4870
**e-mail**	rmboase@bigpond.net.au; robyn.boase@jcu.edu.au
**Nationality**	: Australian
**Tertiary Education**	: University of Adelaide,
	: Royal Australasian College of Dental Surgeons
	: QUT-Graduate Certificate in Management
**Dental Registration**	: Dental Board of Australia - DEN0001378829

<u>Tertiary Academic Record</u>

1977-80	Undergraduate studies, **Graduated BDS, Dec 1980** **Distinctions**; Genetics, General and Dental Histology, **Credits**; Physics, Oral Anatomy, Restorative Dentistry 11, Human Physiology, Microbiology, General Pathology, Pharmacology and Therapeutics, Oral Pathology, General Surgery, Restorative Dentistry V, and Oral Surgery.
1983	Royal Australasian College of Dental Surgeons **Primary Examination FRACDS** (passed at the first attempt)
1987	Royal Australasian College of Dental Surgeons **Final Examination FRACDS** (Children's Dentistry) passed at the first attempt.
1989-1990	**Graduated BSc**; **Distinctions** in Reproductive Biology and Neuroanatomy and neuroendocrinology, **Credits** in Special Sense Organs and Social Biology
2003	**Graduate Certificate in Management, Queensland University of Technology**

Employment Record

Current Employment

2012 – 2015

Associate Professor Preventive Dentistry and Indigenous Oral Health
James Cook University, Full time 2012 - 2013, Part-time 2014-current
Smithfield Cairns Qld.

Duties include
Providing Preventive Dentistry, General Dentistry and Dental Public Health lectures to Dentistry students in Years 1,2,3,4,5.

Providing clinical supervision to students in the JCU Dental Clinic and Simulation Clinic, Year 3 Subject Coordinator 2012 – 2014
Supervision of 4th year Research Projects
Committee member, Clinical Governance Committee, Teaching and Learning Committee.

2001-2011

Previous Employment

Director of Oral Health, Cairns & Hinterland Health Service District

The Director of Oral Health CHHSD has responsibility for over 100 staff and a significant budget.

Adjunct Appointment – 2009 - 2011

Associate Professor (Clinical) at James Cook University School of Dentistry
Duties Lectures and Tutorials to Dental Students

Work History 1990 - 2001

Work in various locations including Cairns Base Hospital, Atherton Hospital, Mareeba Clinic, Cooktown Clinic, various School Dental vans and Cape York clinics as well as in a private practice at Smithfield.

Work History 1983 – 1989 South Australia

1989-90
Head of Unit, Gilles Plains Community Dental Clinic, SADS
The Gilles Plains Clinic is a busy regional public dental clinic.
1988 Acting Vice Principal, School of Dental Therapy, Somerton Pk, SA
1985-87 Tutor Dentist, School Of Dental Therapy,
1983-84 Dental Officer, Community Dental Service, Somerton Pk

Work History 1981 - 1983

1981-83 Solo **Private practice** in Hyde park, and as an assistant to Dr J. Burke at Clovercrest and North Haven, suburbs of Adelaide, SA

University experience

1975-90	**University Tutor**
1989-90	Dental Materials Research, a Project with Dr Owen Makinson to select a fissure sealant for the SADS.

Pre-dental employment

Education Dept of SA

1969-75	**Secondary School Teacher** in Biology and Science.
1976	Part time Matriculation Biology teacher at St. Peters Girls Collegiate School.

Other Professional Activities

- Current and long term Member of the ADA (Member ROHAP)
- Leader during the consultation and formulation of the new Rural and Remote section of the new National Oral Health Plan
- Member of the Dental Advancement Society (1982- 1990)
- Member of the Dental Health Education Committee of the ADA (1984-1990)

Referees on request

Yvonne Cadet-James
Curriculum Vitae Outline – April 2015

Contact: (07) 47815594 work; 0428 180 246 mob; (07) 47814033fax; yvonne.cadetjames@jcu.edu.au
Nationality: Australian - Aboriginal Language Group – Gugu Badhun People -Valley of Lagoons, Qld

Qualifications

- Grad Dip of Education (Adult) NTU; Bachelor of Nursing NTU; Dip of App Science NTU; RN, RM; FACN

Professional Membership and Scholarship

- Fellow of the Australian College of Nursing (current)
- Member – Queensland Indigenous Education Consultative Committee (current)
- Member – National Aboriginal and Torres Strait Islander Higher Education Consortium (current)
- Chancellor – Batchelor Institute of Indigenous Tertiary Education (2008 – 2012).
- National Health and Medical Research Council: Member and Chair – Indigenous Health Research Grant Review Panel – Project and Strategic Grants (2001 – 2010) Indigenous Grant Review Panel 2012; 2013

Current Employment

Professor - Chair of Indigenous Australian Studies; School of Indigenous Australian Studies; JCU

Indigenous Research Scholarship

- Input into the NHMRC Values and Ethics: Guidelines for Ethical Conduct in Aboriginal & Torres Strait Islander health research
- Member of Committee for the development of NHMRC Keeping Research on Track: a guide for Aboriginal & Torres Strait Islander peoples about health research ethics
- Member of Committee for the development of the NHMRC Road Map 11 strategic framework for improving the health of Aboriginal & Torres Strait Islander people through research.
- Member JCU Human Ethics Committee 2003 – 2012; JCU Human Research Ethics Monitor for Indigenous research (current)
- Grant Reviewer – NHMRC and ARC
- Facilitator - JCU Indigenous Research Protocol Workshops; Community Research Workshops

Grants 2010 - 2015

- Year 2015 – 2017. Amount $612,845. ARC. Developing a framework for measuring Indigenous research benefit. Felecia Watkin Lui, Roxanne Bainbridge, **Yvonne Cadet-James**, Komla Tsey, Janya Mccalman.
- Year 2014 – 2018. Amount $824,875. NHMRC. Psycho-social resilience, vulnerability and suicide prevention: a mentoring approach to modifying suicide risk for remote Indigenous students who are compelled to relocate to boarding schools Roxanne Bainbridge, Janya McCalman, Komla Tsey, Ernest Hunter, Patrick McGorry, Mark Wenitong, **Yvonne Cadet-James**, Anthony Shakeshaft, Christopher Doran, Christopher LaLonde.
- Year 2013 – 2014. Amount $171,231. Menzies School of Health Research. Health Promotion capacity enhancement. Komla Tsey, Janya McCalman, **Yvonne Cadet-James**, Roxanne Bainbridge, Catherine Brown and Mary Whiteside
- Year 2012 – 2013. Amount $50,000. ACT for Kids. Safe House Program. Family Wellbeing Evaluation Framework. Komla Tsey; Janya McCalman, Catherine Brown, **Yvonne Cadet-James**, Andrew Searles, Mary Whiteside.
- Year 2012 – 2013. Amount $34,000. Qld Health. Project: Health Priority setting for Cape York Family-Centred Health Project. CIs Komla Tsey, Chris Doran, Anthony Shakeshaft, Andrew Searles, **Yvonne Cadet-James**, Stephen Begg, Janya McCalman.
- Year 2011 - 2015 years. Amount $1,113,000. Project: Qld Health.A Program to enhance the social and emotional wellbeing of Indigenous Australians in Queensland. CIs **Yvonne Cadet-James**, Komla Tsey, Melissa Haswell-Elkins.
- Year 2011 - 2013. Amount $279,375. NHMRC. Project Cannabis withdrawal among Indigenous inmates in north Qld. CIs Alan Clough, Jan Copeland, Petra Buettner, **Yvonne Cadet-James**.
- Year 2010 - 2012. Amount $568,500. NHMRC. Project: Economic evaluation of interventions to reduce the burden of harm from alcohol misuse in Indigenous Australians. CIs Chris Doran, Eric Vox, **Yvonne Cadet-James**, Anthony Shakeshaft, Komla Tsey, Melissa Haswell-Elkins.
- Year 2010 - 2012. Amount $784,875. NHMRC. Project Indigenous community action to reduce harms associated with heavy cannabis use in Cape York. CIs Alan Clough, Darlene McNaughton, Jan Copeland, Ernest Hunter, **Yvonne Cadet-James**, Petra Buettner, Peter D'Abbs.
- Year 2009 - 2010. Amount $10,000. Office of the Public Trustee. Project: Indigenous Queenslanders and Impaired Decision Making. CIs. **Yvonne Cadet-James**, Valda Wallace.
- Year 2008 - 2009, Amount $12,750 Project: Indigenous Land Corporation. Evaluation of the Jumbun Market Garden. Indigenous Land Corporation Grant. CIs. **Yvonne Cadet-James**, Sue McGinty, Agnes Hannan, Dallas Young.
- Year 2007 - 2010, Amount $295,144.00. NHMRC. Project: Palliative Care for Aboriginal and Torres Strait Islander people with end-stage renal disease: an action research initiative. NHMRC grant. CIs Stephen Margolis, Komla Tsey, Valmae Ypinazar, **Yvonne Cadet-James**, Murty Mantha, Sue McGinty, Will Cairns.

Publications 2011 - 2015

- Madden, Dianna., Atkinson, Ian**., Cadet-James, Yvonne**., Watkin-Lui, Felecia. (2014). Probes and Prototypes: A Participatory Action Research Approach to CoDesign, 10(1). pp.31-45.

- Bainbridge, Roxanne., Tsey, Komla., Brown, Catherine., McCalman, Janya**.,** **Cadet-James, Yvonne**., Margolis, Steven., Ypinazar, Valmae. (2013).Coming to an ethics of research practice in a remote Australian Aboriginal community. Contemporary Nurse. 46(1): 18-27.
- Gould, Gillian S., Munn, Joanne, Avuri, Sandra, Hoff, Susan, **Cadet-James, Yvonne**, McEwen, Andy, and Clough, Alan R. (2013). "Nobody smokes in the house if there's a new baby in it": Aboriginal perspectives on smoking in pregnancy and in the household in regional NSW Australia. Women and Birth, 26(4).pp.246-253.
- Campbell S, Bohanna I, Swinbourne A, **Cadet-James Y**, McKeown D, McDermott R. Stages of Change, Smoking Behaviour and Readiness to Quit in a Large Sample of Indigenous Australians Living in Eight Remote North Queensland Communities. *International Journal of Environmental Research and Public Health*. 2013; 10(4):1562-1571.
- Rogerson, B.J., Copeland, J., Buttner, P., Bohanna, I.K., **Cadet-James, Y.L**., McEwan, Andy, and Clough, A.R. (2013). An explorative study of cannabis withdrawal among Indigenous Australian prison inmates; study protocol. MBM Open, 3(5).pp.1-8.
- Madden, Dianna, **Cadet-James, Yvonne**, Watkin-Lui, Felecia, and Atkinson, Ian (2012) Healing through ICT: enhancing wellbeing in an Aboriginal community. Journal of Tropical Psychology, 2 . pp. 1-9.
- Kilcullen, M., Swinbourne, A., and **Cadet-James, Y**. (2012) Cultural identity and mental health: Australian Aboriginal and Torres Strait Islander health and wellbeing. Psychology & Health, 27 (s1). pp. 67-68.
- Kilcullen, Meegan, Swinbourne, Anne, and **Cadet-James, Yvonne** (2012) Factors affecting resilience of Aboriginal and Torres Strait Islander grandmothers raising their grandchildren. In: Urban health: strengthening our voices, culture and partnerships. Australian Institute of Aboriginal and Torres Strait Islander Studies (AIATSIS), Canberrra, ACT, pp. 31-46.
- Kilcullen, M.L., Swinbourne, A., and **Cadet-James, Y**. (2012) Mental health and connectedness: exploring Aboriginal and Torres Strait Islander perspectives. Journal of Paediatrics and Child Health, 48 (s1). p. 62.
- Whiteside, Mary, Tsey, Komla, and **Cadet-James, Yvonne** (2011) A theoretical empowerment framework for transdisciplinary team building. Australian Social Work, 64 (2). pp. 228-232.
- Haswell, Melissa R., Kavanagh, David, Tsey, Komla, Reilly, Lyndon, **Cadet-James, Yvonne**, Laliberte, Arlene, Wilson, Andrew, and Doran, Chris (2010) Psychometric validation of the Growth and Empowerment Measure (GEM) applied with Indigenous Australians. Australian and New Zealand Journal of Psychiatry, 44 (9). 791-799.
- Berry, Helen, Butler, James R.A., Burgess, C. Paul, King, Ursula G., Tsey, Komla, **Cadet-James, Yvonne L**., Rigby, C. Wayne, and Raphael, Beverley (2010) Mind, body, spirit: co-benefits for mental health from climate change adaptation and caring for country in remote Aboriginal Australian communities. NSW Public Health Bulletin, 21 (5-6). pp. 139-145.
- McCalman, Janya, Tsey, Komla, Wenitong, Mark, Wilson, Andrew, McEwan, Alexandra, **Cadet-James, Yvonne**, and Whiteside, Mary (2010) Indigenous men's support groups and social and emotional wellbeing: a meta-synthesis of the evidence. Australian Journal of Primary Health

Conference and Forum Presentations 2010 - 2014

- 2013 Townsville – JCU School of Environmental and Earth Sciences Post Graduate Conference. Indigenous Research Perspectives and Ethics. Invited Speaker
- 2013 Bateman's Bay NSW. Healing the Scars Conference: Aboriginal and Torres Strait Rural and Remote Drug and Alcohol Conference. Empowerment addressing drug and alcohol issues. Invited Speaker.
- 2013 Cairns – International Indigenous Research for Sustainability Conference. Challenges of working in multi disciplinary and multi cultural research and research education supervision teams.
- 2012 Auckland NZ. International Indigenous Development Research Conference. Enhancing Indigenous Distinctiveness – Understanding the relationship between Indigenous Knowledge Creation, Research and Indigenous Transformation. Yvonne Cadet-James and Felecia Watkin-Lui.
- 2012 Townsville – JCU Public Lecture Series. Captain Cook, Transits of Venus and the Exploration of North Queensland. "Encountering the Other: Indigenous North Queensland and the Endeavour Voyage" Yvonne Cadet-James and Agnes Hannan.
- 2012 Magnetic Island – Australian Workforce Council – Early Childhood Conference – Learning and understanding from listening to Indigenous people who experience social exclusion - Keynote speaker.
- 2011 Cairns – Australian Workforce Council – Early Childhood Professional Development forum – Presentation and facilitation - Social Inclusion in the workplace.
- 2011 Brisbane – My life journey – success – Motivational key note speaker for Qld Health Nurses and Midwives Conference.
- 2010 Geneva - International Health Promotion and Education Conference. Evaluation of the Implementation of a Market Garden in an Indigenous Community. Dallas Young and Yvonne Cadet-James.

References

Professor Komla Tsey
Tropical Leader – Cairns Institute. School of Education
Faculty of Arts, Education & Social Sciences, JCU
Ph: (07) 40421257; 0428282806m; komla.tsey@jcu.edu.au

Professor Sue McGinty
Indigenous Australian Studies
Office of the SDVC, JCU
Ph: (07)47814642; 0422744090m; sue.mcginty@jcu.edu.au

Surani Fernando (s2853563)

Official Address
9.15, Building G40,
Griffith University, Gold Coast
QLD 4222, Australia

Residence
62/3, Arundel Links Estate, Arundel
QLD 4214, Australia
Email: surani.fernando@griffith.edu.au
Office +61 (0) 7 55527695
Mobile +61 (0) 4 10219693

I was a full time engaged visiting research fellow at Griffith Health Institute, Griffith University Gold Coast Campus since 2012. At present, as a full time PhD student I am leading the oral health arm of the Environments for Healthy Living prospective cohort study which aims to investigate the association of dental caries with a multitude of risk factors and behaviours including heritability for dental caries.

**2014 February**	Full time PhD student, Griffith University, Gold Coast Campus
**2012-2014**	Research Fellow, Griffith Health Institute, Griffith University, Gold Coast, Australia
**2011-2012**	Senior Registrar, Health Education Bureau, Colombo, Sri Lanka
**2008-2011**	MD (Community Dentistry), Post Graduate Institute of Medicine, Faculty of Medicine, Colombo, Sri Lanka
**2007-2008**	Community Dental Surgeon, Health Education Bureau, Sri Lanka
**2006-2007**	MSc (Community Dentistry), Postgraduate Institute of Medicine, Faculty of Medicine, Colombo, Sri Lanka
**2004-2006**	Dental Surgeon, District Hospital Thangalla, Sri Lanka
**2001-2004**	General Dental Practitioner, Sri Lanka
**2001**	BDS, University of Peradeniya, Sri Lanka

**Professional Qualifications**	Trainer in Behaviour Change Communication (BCC) and Communication for Behavioural Impact (COMBI)<br>Visiting Lecturer at the Faculty of Medicine, University of Sri Jayewardenepura, Sri Lanka<br>Sessional Staff member at Griffith Dental School

Post-doctoral research fellow, Griffith University Australia, 2012 May – 2014 (February)

Senior Registrar, Health Education Bureau, Colombo, Sri Lanka 2011-2012 (May)

- duties and responsibilities included Health Promotion of communities
- Oral health promotion
- Prevention and Control of communicable diseases
- Reproductive Health
- Production of IEC materials
- Heath Promotion Policy for Sri Lanka
- Mass media and Communication
- Post of Visiting Lecturer Visiting lecturer, the Faculty of Medicine, University of Sri Jayewardenepura

Griffith UNIVERSITY

Northern Peninsula Area (NPA) Child Oral Health Survey

Campus (circle)

Injinoo Junior	Bamaga Junior	Bamaga Senior

Student name: ______ Class: ______

Date of examination (dd/mm/yy) | | | | | | |

Identification number | | | | |

Examiner | | |

Duplicate | |

ICDAS-II ENTRY

DECIDUOUS DENTITION

QUADRANT 1

	MES	OCC	DIS	BUC	LIN
55					
54					
53					
52					
51					

QUADRANT 2

	MES	OCC	DIS	BUC	LIN
61					
62					
63					
64					
65					

QUADRANT 4

	MES	OCC	DIS	BUC	LIN
81					
82					
83					
84					
85					

QUADRANT 3

	MES	OCC	DIS	BUC	LIN
75					
74					
73					
72					
71					

PERMANENT DENTITION

QUADRANT 1

	MES	OCC	DIS	BUC	LIN
18					
17					
16					
15					
14					
13					
12					
11					

QUADRANT 2

	MES	OCC	DIS	BUC	LIN
21					
22					
23					
24					
25					
26					
27					
28					

QUADRANT 4

	MES	OCC	DIS	BUC	LIN
41					
42					
43					
44					
45					
46					
47					
48					

QUADRANT 3

	MES	OCC	DIS	BUC	LIN
38					
37					
36					
35					
34					
33					
32					
31					

TREATMENT NEED

Treatment Required?

Yes	
No	

Urgent?

Yes	
No	

INFORMATION SHEET: PARENTS/GUARDIANS

Project title:
Effectiveness, cost-effectiveness and cost-benefit of a single annual professional intervention for the prevention of childhood dental caries in a remote rural Indigenous community

Project team:

- **Chief Investigator:**
  Professor Newell Johnson (Griffith University)
  Contact Phone: 07-5552 9306 ; Mobile: 0448 954 344
  Contact E-mail: n.johnson@griffith.edu.au

- **Principal investigators:**
  Professor Ratilal Lalloo (Griffith University), Professor Jeroen Kroon (Griffith University), Mrs Valda Wallace (James Cook University), Associate Professor Lisa Jamieson (University of Adelaide), Dr Ohnmar Tut (Griffith University), Dr Sanjeewa Kularatna (Griffith University)

Why is the project being undertaken?
To reduce tooth decay in primary and secondary school-going children in the Northern Peninsula Area by application of a single annual dental preventive intervention.

What will you and your child be asked to do?
Your child will receive a separate Information Sheet to explain the project. Kindly ensure that your child understands what they will be asked to do and to also respect their decision should they not wish to take part.
Should you agree to their participation, kindly sign the consent form and request your child to hand this back to their teacher by [date to be inserted]
Your child's involvement will involve a simple examination of his/her mouth and teeth completion of a questionnaire, treatment in the Bamaga Hospital dental clinic if required, and the application of the preventive intervention.

The basis by which participants will be selected or screened
This project is undertaken with the full permission of the Northern Peninsula Area State College and the Queensland Department of Education and The Queensland Health Torres and Cape Hospital and Health Service. Participants will consist of school-going children who attend one of the three campuses on the days the dentists come, and whose parents/guardians have given consent for participation in the project.

The expected benefits of the project
Expected benefits of this study include improved oral and general health of children within the community and an understanding of the costs associated with the intervention provided. If your child needs any treatment we will tell you.

Risks to your child
Over and above this treatment being provided to participants, the preventive intervention will result in improved oral and general health of children within the community.

Confidentiality
The information about your child as his/her mouth will be stored under the strict control of the Chief Investigator and your privacy will be upheld. Any data on treatment need as found in our survey will be shared with the Well Persons' Health Check survey team to ensure immediate treatment.

Participation is voluntary
Participation is completely voluntary and if you have any questions or concerns feel free to ask any member of the survey team. Your child is free to withdraw without comment or penalty.

Mechanism for distribution and return
Kindly ensure that your child returns the completed informed consent form to their class teacher at the Northern Peninsula Area State College by **[date to be inserted].**

Questions / further information
Should you have any questions or require further information, please do not hesitate to ask a member of the survey team or contact the Chief Investigator.

The ethical conduct of this project
Griffith University conduct research in accordance with the *National Statement on Ethical Conduct in Human Research*. If potential participants have any concerns or complaints about the ethical conduct of the research project they should contact the Manager, Research Ethics on 07 3735 5585 or **research-ethics@griffith.edu.au**.

Feedback to you
The results of the project will be made available to the Bamaga community, Queensland Department of Education and The Queensland Health Torres and Cape Hospital and Health Service. You may ask Professor Johnson to see the results if you wish. No participant will be identified in the final report.

Expressing Consent
By signing, completing the front page of the attached Consent Form and returning it to the Northern Peninsula State College, you confirm that you have read and understood the Information Sheet and wish for your child to participate in this survey.

PLEASE DETACH THIS INFORMATION SHEET AND RETAIN IT FOR YOUR LATER REFERENCE

Griffith UNIVERSITY

CONSENT FORM

Project Title:
Effectiveness, cost-effectiveness and cost-benefit of a single annual professional intervention for the prevention of childhood dental caries in a remote rural Indigenous community

By signing below and returning the form to the Northern Peninsula State College I confirm that I have read and understood the information sheet and in particular have noted that:

- I understand that my child's participation in this project is voluntary;
- I understand that my child's involvement will include a simple examination of his/her mouth and teeth, treatment if required and application of a preventive intervention;
- I understand the risks involved;
- I have had any questions answered to my satisfaction and understand that if I have any additional questions I can contact the survey team and/or Chief Investigator;
- I understand that my child is free to withdraw at any time, without comment or penalty; and
- I understand that I can contact the Manager, Research Ethics, at Griffith University Human Research Ethics Committee on 3735 5585 (or research-ethics@griffith.edu.au) if I have any concerns about the ethical conduct of the project.

Parent/Guardian First and Last Name	
Parent/Guardian Signature	
Date	

Please complete the following detail on this page only for your child:

Child's First Name		
Child's Last Name		
Child's Date of Birth		
Child's actual age		
Child's Sex (circle)	Male	Female

**I agree for my child to participate in this project (circle)**	Yes	No

Kindly return this form to your class teacher at NPA State College by [date to be inserted].

INFORMATION SHEET: STUDENTS

Project title:

Effectiveness, cost-effectiveness and cost-benefit of a single annual professional intervention for the prevention of childhood dental caries in a remote rural Indigenous community

Project team:

- **Chief Investigator:**
  Professor Newell Johnson (Griffith University)
  Contact Phone: 07-5552 9306 ; Mobile: 0448 954 344
  Contact E-mail: n.johnson@griffith.edu.au

- **Principal investigators:**
  Professor Ratilal Lalloo (Griffith University), Professor Jeroen Kroon (Griffith University), Mrs Valda Wallace (James Cook University), Associate Professor Lisa Jamieson (University of Adelaide), Dr Ohnmar Tut (Griffith University), Dr Sanjeewa Kularatna (Griffith University)

Why are we doing this project?

We wish to treat any tooth decay and prevent this from happening again in future. It is important for us to see if this has made a difference to the health of your teeth.

What you will be asked to do?

Please hand the papers given to you at school to your parents/guardians to read.
Please read this paper and ask your parent/guardian to explain to you if you do not understand anything it says.
Please bring back to school the form that your parent/guardian will give you and give it to your teacher by **[date to be inserted]**.
On the day of our visit we will have a quick look at your teeth. This should not take longer than 10 minutes.
Should we find that you need any treatment we will ask the dental clinic to fix your teeth.

Who will take part?

We will look at the teeth of all children from the Northern Peninsula Area State College

Have we been given permission to do this project?

Yes, this project has the full permission of Queensland Health and Education Queensland and we will also ask your parent/guardian to give us permission to look at your teeth.

Do I have to take part?

You can tell us at any time if you do not want to take part. But is will be very important to us that you do. It will also be important to you to know if your teeth are healthy and if you will need to have anything done to fix them.

What if I have any questions?

If you are not sure about anything please ask us when we visit the school or ask your parent guardian to contact us.

Griffith UNIVERSITY

Northern Peninsula Area (NPA) Child Oral Health Survey

INTERVIEWER: PLEASE READ THIS TO THE PARTICIPANT BEFORE STARTING THE QUESTIONNAIRE

- We would like your help. We wish to collect information about your dental health as part of the prevention program we are conducting
- All information you provide will be strictly confidential
- All of the questions here refer to 'you'
- All of the questions are valuable to us, so please answer them to the best of your knowledge
- Please tell me if you do not understand any of the questions

THIS IS THE START OF THE QUESTIONNAIRE

1. About you

1.1 Gender	Male ☐1	Female ☐2
1.2 Your date of birth *(day/month/year)*	/ /	
1.3 Your age at last birthday *(in years)*		
1.4 Name of the school you attend		
1.5 Your current school year level		

2. Residential history

Have you lived all your life in the NPA?		
Yes ☐1	From which year?	To which year?
No ☐2 Where else have you lived?		
Name of town/city?	From which year?	To which year?

3. Perceived general and oral health

	Excellent	Very Good	Good	Fair	Poor
3.1 Do you think your general health is……….	☐1	☐2	☐3	☐4	☐5
3.2 Do you think your dental health is……….	☐1	☐2	☐3	☐4	☐5
3.3 On a scale of 1 to 10, where would you rate the importance of your dental health?	☐1 ☐2 ☐3 ☐4 ☐5 ☐6 ☐7 ☐8 ☐9 ☐10<br>*1=most important and 10=least important*				

4. Oral health behaviours

4.1 Do you have a toothbrush?	☐1 Yes<br>☐2 No → *Go to question 4.6*
4.2 How often do you brush your teeth?	☐1 Less than once a day<br>☐2 Once a day<br>☐3 Twice a day<br>☐4 More than twice a day
4.3 In the last week how often did you brush your teeth?	☐1 Less than once a day<br>☐2 Once a day<br>☐3 Twice a day<br>☐4 More than twice a day
4.4 How often do you use toothpaste when brushing your teeth?	☐1 Always<br>☐2 Most times<br>☐3 Sometimes<br>☐4 Never → *Go to question 4.6*
4.5 What brand of toothpaste do you use?	
4.6 Do you use bush medicine/food to look after your teeth?	☐1 Yes → *Go to question 4.7*<br>☐2 No

4.7 Can you give examples of the bush medicine/food you use to look after your teeth?

……

……

……..

……

……

……..

5. Dental visits

5.1 In the last 12 months, how many times have you visited the dentist?	
5.2 What is the reason why you usually visit the dental clinic?	☐1 Check-up<br>☐2 Relief of pain<br>☐3 Problem<br>☐4 Other → *Please specify below:*
Other reasons:<br>……………………………………………………………………………………………………<br>……………………………………………………………………………………………………	
5.3 When was the last time you visited the dental clinic?	☐1 Less than 6 months ago<br>☐2 6 months to less than 12 months ago<br>☐3 1 year to 2 years ago<br>☐4 More than 2 years ago
5.4 The last time you went to the dental clinic, what was the reason?	☐1 Check-up<br>☐2 Relief of pain<br>☐3 Problem<br>☐4 Other → *Please specify below:*
Other reasons:<br>……………………………………………………………………………………………………<br>……………………………………………………………………………………………………	

6. Diet

On a typical day, do you have any of the following:

6.1 Fruit (for example apple, banana, pear)	☐1 Yes<br>☐2 No
6.2 Vegetables (for example, potato, beans, carrots)	☐1 Yes<br>☐2 No
6.3 Meat (for example chicken, beef, fish)	☐1 Yes<br>☐2 No
6.4 Dairy (for example plain milk, cheese, yoghurt)	☐1 Yes<br>☐2 No
6.5 Soft drink (for example coke, fanta, lemonade)	☐1 Yes<br>☐2 No
6.6 Fruit juice (for example orange, tropical, pineapple)	☐1 Yes<br>☐2 No
6.7 Sweets and lollies (for example cake, biscuits, chocolate)	☐1 Yes<br>☐2 No
6.8 Syrups, jams and sweet spreads (for example nutella, honey, maple syrup)	☐1 Yes<br>☐2 No
6.9 Do you add sugar to your cereal, tea, coffee or milo?	☐1 Yes → *Go to question 6.10*<br>☐2 No
6.10 How many teaspoons do you add in total?	

7. General Child Quality of Life (CHU-9D)

Instructions: These questions ask about how the participant is **today**

7.1 Worried

☐ 1	You don't feel worried today
☐ 2	You feel a little bit worried today
☐ 3	You feel a bit worried today
☐ 4	You feel quite worried today
☐ 5	You feel very worried today

7.2 Sad

☐ 1	You don't feel sad today
☐ 2	You feel a little bit sad today
☐ 3	You feel a bit sad today
☐ 4	You feel quite sad today
☐ 5	You feel very sad today

7.3 Pain

☐ 1	You don't have any pain today
☐ 2	You have a little bit of pain today
☐ 3	You have a bit of pain today
☐ 4	You have quite a lot of pain today
☐ 5	You have a lot of pain today

7.4 Tired

☐ 1	You don't feel tired today
☐ 2	You feel a little bit tired today
☐ 3	You feel a bit tired today
☐ 4	You feel quite tired today
☐ 5	You feel very tired today

7.5 Annoyed

☐ 1	You don't feel annoyed today
☐ 2	You feel a little bit annoyed today
☐ 3	You feel a bit annoyed today
☐ 4	You feel quite annoyed today
☐ 5	You feel very annoyed today

7.6 School Work/Homework (such as reading, writing, doing lessons)

☐ 1	You have no problems with you schoolwork/homework today
☐ 2	You have a few problems with your schoolwork/homework today
☐ 3	You have some problems with your schoolwork/homework today
☐ 4	You have many problems with your schoolwork/homework today
☐ 5	You can't do your schoolwork/ homework today

7.7 Sleep

☐ 1	Last night you had no problems sleeping
☐ 2	Last night you had a few problems sleeping
☐ 3	Last night you had some problems sleeping
☐ 4	Last night you had many problems sleeping
☐ 5	Last night you couldn't sleep at all

7.8 Daily routine (things like eating, having a bath/shower, getting dressed)

☐ 1	You have no problems with your daily routine today
☐ 2	You have a few problems with your daily routine today
☐ 3	You have some problems you're your daily routine today
☐ 4	You have many problems with your daily routine today
☐ 5	You can't do your daily routine today

7.9 Able to join in activities (things like playing out with their friends, doing sports, joining in things)

☐ 1	You can join in with any activities today
☐ 2	You can join in with most activities today
☐ 3	You can join in with some activities today
☐ 4	You can join in with a few activities today
☐ 5	You can join in with no activities today

8. Oral Health Impact Profile (OHIP–14)

0=Never; 1=Hardly ever; 2=Occasionally; 3=Fairly often; 4=Very often

HOW OFTEN during the PAST 4 WEEKS …	0	1	2	3	4
8.1 have you had trouble pronouncing any words because of problems with your teeth, mouth or dentures	☐0	☐1	☐2	☐3	☐4
8.2 have you felt that your sense of taste has worsened because of problems with your teeth, mouth or dentures?	☐0	☐1	☐2	☐3	☐4
8.3 have you had painful aching in your mouth?	☐0	☐1	☐2	☐3	☐4
8.4 have you found it uncomfortable to eat any foods because of problems with your teeth, mouth or dentures?	☐0	☐1	☐2	☐3	☐4
8.5 have you felt self-conscious because of problems with your teeth, mouth or dentures?	☐0	☐1	☐2	☐3	☐4
8.6 have you felt tense because of problems with your teeth, mouth or dentures?	☐0	☐1	☐2	☐3	☐4
8.7 has your diet been unsatisfactory because of problems with your teeth, mouth or dentures?	☐0	☐1	☐2	☐3	☐4
8.8 have you had to interrupt meals because of problems with your teeth, mouth or dentures?	☐0	☐1	☐2	☐3	☐4
8.9 have you found it difficult to relax because of problems with your teeth, mouth or dentures?	☐0	☐1	☐2	☐3	☐4
8.10 have you been a bit embarrassed because of problems with your teeth, mouth or dentures?	☐0	☐1	☐2	☐3	☐4
8.11 have you been a bit irritable with other people because of problems with your teeth, mouth or dentures?	☐0	☐1	☐2	☐3	☐4
8.12 have you had difficulty doing your usual jobs because of problems with your teeth, mouth or dentures?	☐0	☐1	☐2	☐3	☐4
8.13 have you felt that life in general was less satisfying because of problems with your teeth, mouth or dentures?	☐0	☐1	☐2	☐3	☐4
8.14 have you been totally unable to function because of problems with your teeth, mouth or dentures?	☐0	☐1	☐2	☐3	☐4

9. About your household

9.1 How many people do you live with?	☐1 One to five<br>☐2 Six to ten<br>☐3 More than 10
9.2 How many people that you live with go to work?	☐1 None<br>☐2 One or more
9.3 Who looks after you?<br>*Describe, for example mother, father, grandmother, aunty, etc.*	

10. Your comment

Please write any other comments the participant might have about their oral health in the space below.

……

……

……

……

……

……

……

………..

THIS IS THE END OF THE QUESTIONNAIRE

Effectiveness, cost-effectiveness and cost-benefit of a single annual professional intervention for the prevention of childhood dental caries in a remote rural Indigenous community

Letters of support:

- Executive Manager: Torres and Cape Hospital and Health Service
- Health Service Chief Executive: Torre Strait Northern Peninsula Hospital and Health Service
- Chief Dental Officer: Queensland Government
- The Mayor: Northern Peninsula Area Regional Council
- Principal: Northern Peninsula Area State College
- Chief Executive Officer: Apunipima Cape Your Health Council

Australian Government
National Health and Medical Research Council

NHMRC

GPO Box 1421 | Canberra ACT 2601
16 Marcus Clarke Street, Canberra City ACT 2600
T. 13 000 NHMRC (13 000 64672) or +61 2 6217 9000
F. +61 2 6217 9100
E. nhmrc@nhmrc.gov.au
ABN 88 601 010 284
www.nhmrc.gov.au

Emeritus Professor Newell Johnson
n.johnson@griffith.edu.au

Dear Emeritus Professor Johnson

********* UNDER EMBARGO *********

This advice and the document/s referred to below are provided under strict **embargo** with the understanding that **they are not to be made public at this time by institutions or recipients**. Any breach of this embargo may be seen as a breach of the *Australian Code for the Responsible Conduct of Research*. NHMRC will notify your Administering Institution when your outcome is no longer under embargo.

****

Application ID: APP1081320
Type: Project
Application Title: Effectiveness, cost-effectiveness and cost-benefit of a single annual professional intervention for the prevention of childhood dental caries in a remote rural Indigenous community

I am pleased to advise that the Minister for Health, the Hon. Peter Dutton MP has approved your application (APP1081320) for National Health and Medical Research Council (NHMRC) Project funding commencing in 2015.

This letter provides you with important information about the offer of Funding made to Griffith University for this application.

Assessment Details

Where available[1], information about the assessment of your application is provided in a separate Application Assessment Summary. This can be accessed via RGMS following the instructions for accessing feedback in the *RGMS User Guide – Awarding Grants*.

Accepting this offer

The offer of Funding for your Application is made under the NHMRC Funding Agreement (the Funding Agreement) between the Australian Government and your Administering Institution. Your Administering Institution is responsible for informing you about the requirements of the Funding Agreement (including its Schedules) the Direct Research Costs guidelines and other applicable policies[2].

[1] An assessment summary is not available for schemes where the NHMRC does not perform the peer review, including NHMRC – EU, CIRM and NIHR. Guidance on interpreting Project Grants Assessment Summaries is available at http://www.nhmrc.gov.au/grants/outcomes-funding-rounds.
[2] Copies of the Funding Agreement, and Direct Research Costs guidelines and other policies are available at http://www.nhmrc.gov.au/grants/administering-grants/nhmrc-funding-agreement

Your Administering Institution has until 30/01/2015 to certify that the information required prior to payment being made (see below) has been entered into RGMS, and to advise NHMRC of its acceptance of the offer. If the offer is not accepted by this date it may lapse. If you wish to discuss this offer of Funding, or have any queries, please contact your Research Administration Officer (RAO).

Information required prior to payment being made

Successful CIAs are required to provide additional ethics information, where applicable, as well as a plain English summary before payments commence. This information must be entered into RGMS by the CIA and certified by the RAO. The *RGMS User Guide - Awarding Grants* provides details on how to enter and certify this data at award. Please note that payments will not be made until this information has been entered into RGMS and all other approvals and conditions have been met. Should you have any questions concerning the provision of such information, please speak to your RAO.

Funding

As set out in the Schedule to the Funding Agreement, the Project APP1081320, has been awarded a total of $1,020,590.20 in funding. Any conditions relevant to receiving the funding are set out in the Schedule to the Funding Agreement and, where applicable, the associated Funding Rules. All expenditure must be in accordance with the requirements of the Funding Agreement.

Participation in NHMRC Peer Review

The NHMRC relies on the participation of the research community to continue to ensure that every application receives expert peer review. The NHMRC is grateful for this enormous contribution which is acknowledged through our website's peer review honour roll.

To ensure that applications for future rounds are appropriately assessed, we are reminding all Chief Investigators, Fellows and Scholars working on NHMRC Funded Research Activities of their obligation to contribute to the peer review process, as set out in clause 23.1 of the Funding Agreement.

As such, we ask that you ensure your CV/Profile information is up to date in RGMS to assist in the identification of appropriate peer reviewers.

Yours sincerely

[Authorised for electronic transmission]

Dr Tony Willis
Acting Executive Director
Research Programs

Enquiries to:	Kathleen O'Brien – ESO
Telephone:	4069 0205
Fax:	4069 0604
Our Ref No:	Receipt EGM #00092
Your Ref No:	

Emeritus Professor Newell W Johnson CMG, FMedSci
Professor of Dental Research
Griffith Health Institute
Building G40, Room 9.16
Gold Coast Campus, Griffith University
Queensland 4222

Dear Professor Johnson

Re: NHMRC project to improve oral health among Indigenous children in the Northern Peninsular Area

Thank you for visiting the Northern Peninsula Area recently and introducing the NHMRC project aimed at improving oral health among Indigenous children in the Northern Peninsula Area.

I am writing in support of your proposal regarding "Effectiveness, cost-effectiveness and cost-benefit of a single annual professional intervention for the prevention of childhood dental caries in a remote rural Indigenous community".

As I indicated in our meeting, I believe this is an excellent proposal and the health service will lend its full support to the initiative. In particular I welcome the proposed survey and Wellness checks, the groove sealing on molars, the application of topical fluoride, together with the range of oral health education and promotion and potential. I believe the program will be highly regarded by the community and inform future programs and policy regarding the oral health of Indigenous children remote communities.

In conclusion the health service applauds the program, it's energetic schedule, and is grateful for the opportunity to support your team in delivering the program.

Kind regards

Andy Marshall
Executive General Manager – North
Torres and Cape Hospital and Health Service

10 March 2015

Executive Office T.I Hospital
163 Douglas Street
Thursday Island Q 4875
Torres and Cape Hospital and Health Service

Contact Details
Phone: 4069 0205
Fax: 4069 0604
Mobile: 0423 127 883
Email: TCHHS-North-EGM@health.qld.gov.au
Web: health.qld.gov.au/torres-cape

Postal Address
PO Box 391
Thursday Island QLD 4870

Research Governance
Governance & Policy

Queensland
Government

Torres Strait–Northern Peninsula
Hospital and Health Service

10th January 2014

Professor N Johnson
School of Dentistry & Oral Health,
Gold Coast Campus,
Griffith University,
QLD 4222

Dear Professor Johnson,

Re: "In Principle" Support for NHMRC Project Grant Application

Thank you for submitting the summary description of the NHMRC project grant application to measure the effectiveness, cost-effectiveness and cost-benefit of a single annual professional intervention for the prevention of childhood dental caries in a remote rural indigenous community.

It is understood that the aim of this study would be to reduce the high prevalence of tooth decay in all school children in the Northern Peninsula Area (NPA) by implementing a single annual dental preventive intervention. This outcome would provide a significant improvement in the oral health status of communities in the Torres Strait – Northern Peninsula area. Currently the dental preventive intervention, as described that require regular re-applications (3 monthly), are not provided in these communities.

Along with the Torres Strait- Northern Peninsula Hospital Health Service - Executive Director of Medical Services and the Senior Dentist I am able to provide "in principal" support for this research project which will involve the invitation to children (600-650) attending the four school campuses in the Northern Peninsula Area to participate in this intervention.

As you have noted in your previous correspondence, the Torres Strait- Northern Peninsula Hospital Health Service Research Governance authorisation for this project is yet to be obtained through the submission and authorisation of Site Specific Approvals (SSAs), this letter of "in principal" support will not negate this requirement. Should you have any queries regarding my advice to you, Rosemary Schmidt, Research Governance Officer, will be pleased to assist you and can be contacted by email on TS-NP-HHS-Research-Governance@health.qld.gov.au

Yours sincerely

Simone Kolaric
Health Service Chief Executive

Thursday Island Hospital
PO Box 391,
Thursday Island
Queensland 4875 Australia
Telephone +61 740690217
Facsimile +61 **7 40690604**
Website www.torres-north.health.qld.gov.au

Queensland Government

Enquiries to: Dr Mark Brown
Chief Dental Officer
Department of Health
Telephone: 3328 9297
File Ref: HC004287

Emeritus Professor Newell Johnson
Professor of Dental Research
Griffith Health Institute
Building G40, Room 9.16
Gold Coast Campus
GRIFFITH UNIVERSITY QLD 4222

Dear Professor Johnson

Thank you for providing me with an overview of your National Health and Medical Research Council project *'Effectiveness, cost-effectiveness and cost-benefit of a single annual professional intervention for the prevention of childhood dental caries in a remote rural Indigenous community'.*

The delivery of oral health services in rural and remote locations continues to be a challenge for Queensland Health, particularly in Indigenous communities. I am pleased to offer my support for your project and applaud your multi-strategy approach to improving oral health outcomes for children in the Northern Peninsula Area. I understand your project has a strong community focus which is so critical.

I am also pleased to support this Griffith University proposal as it further builds on the already strong links in oral health between Queensland Health and Griffith University. I am further pleased that with your extensive research record you will lead this research project. This is a timely initiative as it comes with Queensland Health recognising the oral health needs in the Torres and Cape area and seeking to actively address these issues. I note *The Health of Queenslanders, 2014, Fifth report of the Chief Health Officer, Queensland* identified indigenous Queenslanders carry a greater burden of ill health and early death than non-indigenous Queenslanders and the disparity is greater than any other population group. I am confident your research will assist in developing strategies that will help to reduce the burden of oral disease across those communities at risk.

I wish you all the best for your project and look forward to being kept aware of your progress.

Yours sincerely

Dr Mark Brown
Chief Dental Officer
6 / 3 / 15

Office
Office of the Chief Dental Officer
Department of Health
Level 1
15 Butterfield Street
HERSTON QLD 4006

Postal
Office of the Chief Dental Officer
Department of Health
PO Box 2368
FORTITUDE VALLEY QLD 4006

Phone
07 3328 9873

SEISIA
NEW MAPOON
BAMAGA
UMAGICO
INJINOO
NPARC
NORTHERN PENINSULA AREA REGIONAL COUNCIL

Northern Peninsula Area Regional Council

PO Box 200 Bamaga, Qld, 4876
Telephone: (07) 40693211
Fax: (07) 4069 3264
ABN: 27 853 926 592

Emeritus Professor Newell W Johnson CMG, FMedSci
Professor of Dental Research
Griffith Health Institute
Building G40, Room 9.16
Gold Coast Campus, Griffith University
Queensland 4222

Tuesday 10th March 2015

Dear Professor Johnson,

NHMRC APPROVED FUNDING- Letter of Support

Please be advised that the Northern Peninsula Area Regional Council writes in full support of the NHMRC Approved Funding and the project that will be carried out throughout the communities in the northern peninsula area.

Council understands that the aim of this project facilitated by The Griffith University is to improve the oral health of indigenous children by (a) assess the effectiveness of an annual oral health preventive intervention in slowing the incidence of dental caries in children in a remote, rural Indigenous setting. (2): To identify the mediating role of known risk factors for dental caries. (3) To assess the cost-effectiveness and cost-benefit of the intervention. Therefore, council writes in support given the importance of our children oral and dental health education; and for Griffith University to implement this project within the required timeframe.

If you have an queries, then please do not hesitate to contact myself or the Mr Danny Sebasio (Deputy CEO) here on 0740 904100.

Yours sincerely

BCharlie

Mr Bernard Charlie
The Mayor
Northern Peninsula Area Regional Council.

NORTHERN PENINSULA AREA STATE COLLEGE

..... Good Pasin ~ Learner ~ Safe ~ Respectful .....

COLLEGE ADMINISTRATION
Sagaukaz Street
BAMAGA QLD 4876

Phone 07 4090 4333
Fax 07 4090 4300

BAMAGA SECONDARY
Sagaukaz Street
BAMAGA QLD 4876

Phone 07 4090 4320
Fax 07 4090 4344

INJINOO PRIMARY
Francis Street
INJINOO QLD 4876

Phone 07 4090 4888
Fax 07 4069 3131

BAMAGA PRIMARY
Anu Street
BAMAGA QLD 4876

Phone 07 4090 4444
Fax 07 4090 4400

Emeritus Professor Newell W Johnson CMG, FMedSci
Professor of Dental Research
Griffith Health Institute
Building G40, Room 9.16
Gold Coast Campus, Griffith University
Queensland 4222

NHMRC PROJECT – Northern Peninsula Area

The Northern Peninsula Area State College has strived for many years to improve the social, emotional and academic outcomes of the 650 plus students in our care. Over the past 6 years in particular, we have made significant gains in fostering learning outcomes for our students.

However, even with the best instructional models, good attendance and a positive attitude, ongoing health issues can sometimes be a significant barrier to a student's success. With incidents of chronic illness high in remote Indigenous communities, it is heartening to see initiatives aimed at finding cost effective and simple solutions to long term poor health.

The NHMRC project, with its focus on preventative intervention is a project worthy of success. This College is happy to give its whole-hearted support to any initiative aimed at reducing the impact of poor health on the learning outcomes of our students.

With that in mind, I have no hesitation in supporting this worthwhile project.

Yours Sincerely,

Gordon Herbertson
Principal

apunipima
CAPE YORK HEALTH COUNCIL

11th March 2014

Emeritus Professor Newel Johnson
Professor of Dental Research
Griffith Health Institute, Griffith University
Building G40, Room 9.16
GOLD COAST CAMPUS QLD 4222

Dear Emeritus Professor Johnson

Your letter of support request was tabled at our Research Governance Committee meeting recently regarding the project titled "Effectiveness, cost-effectiveness and cost –benefit of a single annual professional intervention for the prevention of childhood dental caries in a remote rural Indigenous community".

Apunipima Cape York Health Council are hopeful that the long term outcomes of this project will mean important oral health benefits for Indigenous people. As dental caries is an independent risk factor for development of diabetes, and remains in its own right one of the most common chronic diseases of Cape York Indigenous people, we definitely agree this is a priority health area for Indigenous Australians.

This proposal fits in our own aims to improve oral health in Cape York, which is very poor by Australian standards. We look forward to active participation with the project should the proposal be successful.

We would appreciate the proposal outcome being fed back to Apunipima Cape York Health Council once it is known.

Yours sincerely

CLEVELAND FAGAN
Chief Executive Officer

1. Substance / Preparation and Company name

Product Name: Conseal-Clear, Conseal-Light Grey, Conseal F (White)

Recommended use: For the protection of pits and fissures.

Manufacturer / Supplier

SDI Limited
3-13 Brunsdon Street, Bayswater
Victoria, 3153, Australia

Telephone:

+61 3 8727 7111 (Business hours)

SDI Inc.
729 N.Route 83, Suite 315
Bensenville 60106 IL, USA

Telephone:

630 238 8300 (Business hours)

Southern Dental Industries Ltd
Block 8, St Johns Court
Swords Road
Santry, Dublin 9, Ireland

Telephone:

+353 1 886 9577 (Business Hours)

SDI Brasil Indústria e Comércio Ltda
Rua Dr. Virgílio de Carvalho Pinto, 612
Pinheiros, São Paulo, 05415-020
Brasil

Telephone:

+55 11 3092 7100 (Business Hours)

Emergency contact number: +61 3 8727 7111

2. Composition / Information on ingredients

Composition:	CAS No.	Wt. %
**Conseal-Clear**		
Acrylic monomer	-	100.0
**Conseal-Light Grey**		
Acrylic monomer	-	80.0
Balance ingredient (non-hazardous)		20.0
**Conseal F (White)**		
Acrylic monomer	-	93.0
Balance ingredient (non-hazardous)		7.0

3. Hazard Identification

Products may cause irritation to the skin, eye and mucous membrane. Ingestion of unpolymerised material may cause gastric irritation. In isolated cases, contact allergies have been reported with acrylic resins. Anyone with known history of resin allergies are advised to seek the advise of a specialist before use.

Risk phrases - **36/37/38**:	Irritating to eyes, respiratory system and skin.
Safety phrases - **26/28**:	In case of contact with eyes, rinse immediately with plenty of water and seek medical advice. After contact with skin, wash immediately with soap and water.
- **3/15/16**:	Keep in a cool place, away from heat and sources of ignition.
- **2**:	Keep out of reach of children.

4. First Aid Measures

Eye (contact):	Flush opened eye with running water for at least 5 minutes. Seek medical attention.
Skin (contact):	Remove contaminated clothing. Wash skin with soap and water. In case of allergic reaction, seek medical attention.
Ingestion:	Seek medical attention.

5. Fire Fighting Measures

Suitable extinguishing media:	Dry powder, vapourizing liquid or foam extinguisher.
Unusual Fire and Explosion Hazards:	Heat can cause polymerization with rapid release of energy which may melt the container.
Special protective equipment:	No special measures required for small quantity. Use water spray to cool container.

6. Accidental Release Measures

Personal precautions:	Not required.
Environmental precautions:	Prevent any spillage from entering waterways, drains or sewage system.
Methods for cleaning up:	Mop up spillage with absorbance paper/cloth soaked in ethanol/acetone.

7. Handling and storage

Handling
Replace caps immediately after use.

Storage
Store in a cool place at temperatures between 10°C and 25°C (50° - 77°F). Keep out of direct light.

8. Exposure controls and personal protection

Respiratory protection:	None required under normal conditions of use.
Hand protection:	Rubber, latex or PVC gloves.
Eye protection:	Safety glasses, goggles or face shield.
General safety and hygiene measures:	Follow good housekeeping practices and good industrial hygiene in handling this material. Remove any naked lights or strong heat sources.

9. Physical and chemical properties

Appearance:	Clear, pale yellow liquid - **Conseal-Clear**.<br>Tooth coloured semi-translucent liquid - **Conseal-Light Grey**<br>White liquid - **Conseal F (White)**.
Odour:	Ester like.
Boiling point:	Gel before boiling.
Melting point:	Not established.
Specific gravity:	1.1 - 1.2
Flash point:	Not established.
Flammable:	Not established.
Autoflammability:	Do not self ignite at room temperature.
Explosive properties:	Do not present an explosion hazard.
Oxidizing properties:	Not established.
Vapour pressure (@ 20°C):	Not established.
Relative density:	Not established.
Solubility:	Insoluble in water.

10. Stability and Reactivity

Stability:	Stable under normal conditions.
Conditions to avoid:	Avoid heat, ignition sources, aging, contamination and intense visible light.

10. Stability and Reactivity (Cont'd)

Materials to avoid: Free radical formers, e.g. peroxides, reducing substances and / or heavy metals ions.

Hazardous decomposition products: None under normal conditions; oxides of carbon when burned.

Hazardous reactivity (polymerization): Heat and intense light can cause polymerization.

11. Toxicological information

Acute toxicity: Irritating to skin, eye and mucous membrane.

Sensitization: No sensitizing effect known. In isolated cases contact allergies have been reported.

12. Ecological information

Self assessment: Slightly hazardous for water. Do not allow large quantities to reach sewage system and waterways.

13. Disposal considerations

Dispose of in accordance with local official regulations.

14. Transport information

Conseal-Clear, Conseal-Light Grey and Conseal F (White) are not classified as Dangerous Goods for air, sea, rail and road transport.

15. Regulatory information

These products are regulated by

TGA
Medical Devices Directive 93/42/EEC
FDA
National regulations

16. Other information

The information provided herein is given in good faith, but no warranty expressed or implied is made.

Prepared by: SDI Limited
3-13 Brunsdon Street, Bayswater
Victoria, 3153, Australia

Phone Number:
+61 3 8727 7111

Department issuing MSDS: Research and Development
Contact: Operations Director

PRODUCT INFORMATION INSTRUCTIONS REORDER CODES

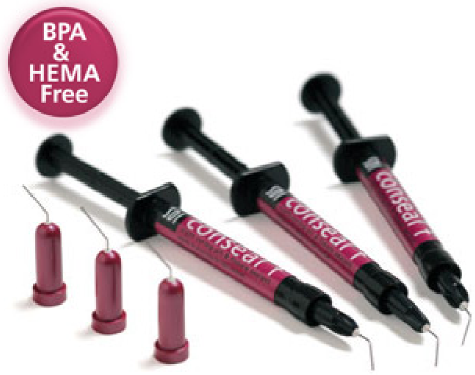

lowest viscosity, deepest penetration

Releases fluoride

Conseal f combines the proven SDI sealant technology with an intensive initial fluoride boost in conjunction with long term fluoride treatment. The unique Conseal f filler component, with its blend of particles and high surface-to-area volume ratios, enables high fluoride release. Fluoride enhances caries prevention, remineralization, and the inhibition of enamel demineralization.

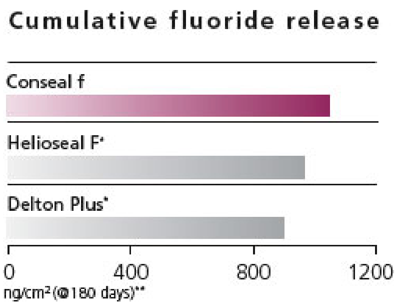

Lowest viscosity

Conseal f is the lowest viscosity sealant.
The ideal low viscosity allows Conseal f to flow faster and deeper into the prepared pits and fissures. A recognized cause of pit and fissure sealant failure is an inability to seal. The tighter seal optimizes Conseal f's mechanical retention and eliminates the space required for bacteria to grow.
Conseal f's tight seal is further enhanced by its unique UDMA resin system's lower shrinkage and by being BIS-GMA free, avoiding the controversy of Bisphenol A.

Conseal f syringe and complet 27 gauge tip

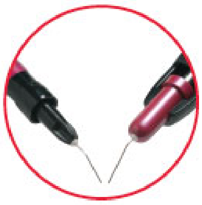

Tip size comparison**

Conseal f's syringe and complet tip diameter opening is only 0.41mm; significantly smaller than the alternatives of 1.5mm or 1.3mm.

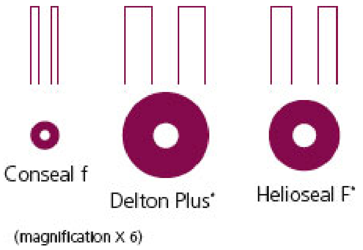

Range of colors

Conseal f is available in opaque white.
Conseal is available in clear and light grey.

Hygienic placement

The Conseal f complets offer a direct and hygienic way of dispensing the sealant, eliminating the risk of cross contamination.

Bio compatible

Conseal f's low water solubility minimizes breakdown of the sealant in the oral environment.

Contains filler

Conseal f is 7% filled with a submicron filler size of 0.04 microns to withstand surface wear.

Clinically proven

Conseal pit and fissure sealants are clinically proven. 5,363 Conseal sealants placed in 774 children found no failures due to the material and established that "sealants contribute significantly to dental public health".[2]

Before treatment

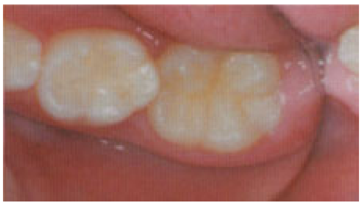

Instructions

We keep an up-to-date archive of all our product instruction sheets.

View the product instruction sheets

MSDS

We keep an up-to-date archive of all our material safety data sheets.

View all MSDS

Brochures

Download and view brochures for the entire range of SDI products.

View brochures

Specials

Click here to get information on special offers in your country.

View current specials

Dental Events

SDI regularly exhibits at dental events all around the world, click here to find one in your area.

Go to the events section

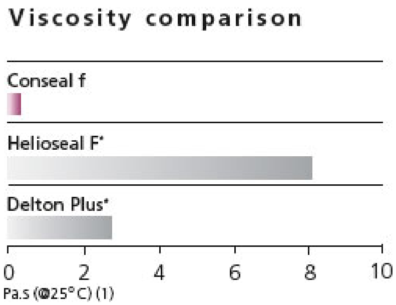

Low viscosity Super Etch LV

SDI has created a specialized blue gel 37% phosphoric acid etchant for sealants, in a direct delivery system. Super Etch LV's lower viscosity improves the acid's surface contact for deeper etchant penetration into the pits and fissures. The deeper acid penetration strengthens the mechanical bond and the retention success of Conseal f sealants.

Deepest penetration

Conseal f is available in direct placement syringes and single dose complets. Both have pre-bent, superfine dispensing tips which facilitate controlled, direct extrusion for faster procedures and allow direct dispensing into previously difficult to reach areas.

The Conseal f nozzle, one third the size of other brands, enhances complete sealing and retention by allowing penetration into the deepest pits and fissures; crevasses that will not admit the dispensing tips of other materials.

Etching with Super Etch LV

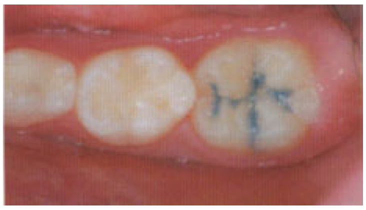

Conseal f application

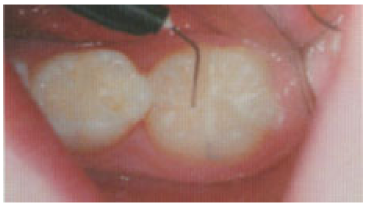

After treatment

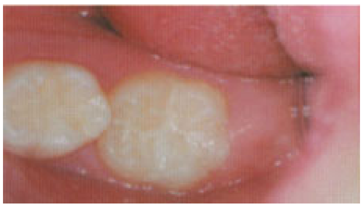

Photos courtesy of Dr Mitsuru Sonoda, D.D.S Hokkaido, Japan

(1) Chryss A (1998), Royal Melbourne Institute of Technology University Rheology and Materials Processing Centre.
(2) Messer LB, Calach H, Morgan MV (1997). The retention of pit and fissure sealants placed in primary school children by Dental Health Services, Victoria. Australian Dental Journal 42:(4);223-239.
* Delton Plus and Helioseal F are not the registered trademarks of SDI Limited.

** Source-Published and SDI test data.

MATERIAL SAFETY DATA SHEET

Number: A-0016-P (E)
Date prepared: January 28, 2003
Date revised: December 20, 2010
Revised detail: Format Changed

1. Chemical Product and Company Identification

Product code: -
Product name: GC Fuji VII / Fuji VII Capsule - Powder
Company Identification:
GC Corporation, 76-1 Hasunuma-Cho, Itabashi-Ku, Tokyo, Japan
Postal code 174-8585, Phone 81-3-3965-1388

Australian supplier:	New Zealand supplier:
Henry Schein Halas Dental Co., Ltd.,	Henry Schein Regional Ltd
Sydney – Head Office	23 William Pickering Drive
44 O'Dea Ave, Waterloo, NSW 2017	Albany, Auckland
Emergency Phone (02) 9697-6288 / 1300 658822	Emergency Phone 0800 764 766
Fax (02) 9697 6250	Fax 0800 808 555

2. Composition / Information on Ingredients

(% chemical components by WT)

Alumino-silicate glass (CAS not listed)	100 %
Iron (III) oxide (CAS 1309-37-1)	<0.1

(See section 8 for exposure guidelines)

3. Hazards Identification

This material is not hazardous according to health criteria of NOHSC Australia.

This material is not classified as Dangerous Goods as per ADG Code

Poison schedule (SUSDP): None

Hazard Classification
None

R/S Phrase
R42/43: May cause sensitization by inhalation and skin contact
S22: Do not breathe dust
S24: Avoid contact with skin
S37: Wear suitable gloves
S45: In the case of accident or if you feel unwell, seek medical advice immediately

4. First Aid Measures

Eyes: Flush eyes with plenty of water for at least 15 minutes, lifting lower and upper eyelids occasionally. Get medical attention.
Skin: Flush skin with plenty of soap and water for at least 15 minutes. Get medical attention if irritation persists.
Ingestion: Do not induce vomiting. Give plenty of water. Get medical attention.

5. Fire Fighting Measures

Flammable Properties
Flash Point: Not flammable

Flammable Limits: Not applicable
Auto Ignition Temperature: Not applicable
Hazardous Combustion Products: Not known
Extinguishing Media: Chemical foam, carbon dioxide, and dry chemical
Fire Fighting Instructions: No special measures required.

6. Accidental Release Measures
Person-related safety precautions: Not required.
Measures for environmental protection: No special measures required.
Measures for cleaning/collecting: Ensure adequate ventilation.

7. Handling and Storage
Handling: Keep container closed when not in use.
Storage: Store in room temperature. Keep away from heat and high humidity.

8. Exposure Controls, Personal Protection
The product does not contain any relevant quantities of materials with critical values that have to be monitored at the workplace.
Respiratory Protection: In case of brief exposure or low pollution use respiratory filter device. In case of intensive or longer exposure use self-contained respiratory protective device.
Skin Protection: Use protective gloves to prevent contact.
Eye Protection: Use tightly sealed goggles to prevent contact.
Exposure Guideline(s): No data
Engineering Controls: No special controls are need.

9. Physical and Chemical Properties
Appearance: Pink or White Powder
Odor: Odorless
Boiling Point: No data
Vapor Pressure: No data
Vapor Density: No data
Solubility in Water: Insoluble
Specific Gravity: No data
Freezing Point: No data
pH: No data
Volatile: No data

10. Stability and Reactivity
Stability: Stable
Incompatibility: Non
Hazardous Decomposition Products: Non
Hazardous Polymerization: Non

11. Toxicological Information
Acute Toxicity:
Primary irritant effect:
On the skin: Irritant to skin and mucous membranes.
On the eye: Irritating effect
Additional toxicological information:
Carcinogenicity: (NTP) No, (IARC) No

12. Ecological Information
Generally not hazardous for water

13. Disposal Considerations
Must not be disposed together with household garbage. Do not allow product to reach sewage

system.
It is recommended to consult local or state regulations regarding proper disposal.

14. Transport Information

IMO Regulations: Not IMO hazardous material
ICAO and IATA Regulations: Not ICAO/IATA hazardous material

15. Regulatory Information

According to the Japanese regulations or code, this material is not classified as a hazardous material.
It is recommended however to consult local regulations of the region or state to determine its identification.

16. Other Information

No specific information available.

The information herein is given in good faith, but no warranty expressed or implied, is made.

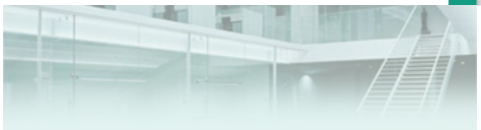

Products

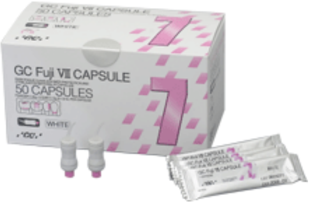

GC Fuji VII CAPSULE (pink or white)

Radiopaque Glass Ionomer Protection Material

Simple and serious protection for teeth at need. GC Fuji VII protects, seals & fortifies.

GC Fuji VII is a high fluoride releasing glass ionomer cement for surface protection of at risk surfaces, provisional restorations and minimal occlusal restorations (white shade)

Advantages

- Very high level of fluoride release 6 times higher then any other glass ionomer
- Can be applied when saliva control is not possible to treat newly erupted molars (partially) covered by tissue
- Chemical adhesion to tooth structure no etching, no bonding
- Self curing conventional GIC with optional command set with VLC unit Indications
- Fissure protection
- Hypersensitivity prevention and control
- Root surface protection
- Intermediate endodontic sealing

Packaging

GC Fuji VII CAPSULE (mixed volume per capsule 0.13 ml)
Pink, box of 50 capsules
White, box of 50 capules

- **Instructions for use Capsules**
- **MSDS Powder**
- **MSDS Liquid**
- **Brochure: For all round protection**
- **References**
- **GC Fuji Glass Ionomer Usage Guide**

© GC Australia 2010 - All rights reserved | Terms and Conditions of Use | Privacy Policy

Material Safety Data Sheet

Infosafe No™ LPXPP	Issue Date :October 2008	ISSUED by COLGATE	CS: 1.4.22

Product Name **COLGATE DURAPHAT**

1. IDENTIFICATION OF THE MATERIAL AND SUPPLIER

**Product Name**	COLGATE DURAPHAT
**Company Name**	Colgate-Palmolive Pty Ltd (ABN 002 792 163)
**Address**	Australia: Level 14, 345 George Street, Sydney NSW 2000 Australia
**Emergency Tel.**	AUS 1800 638 556, NZ: 0800 764766
**Telephone/Fax Number**	Tel: AUS (02) 9229 5600 NZ: 04 576 6700<br>Fax: AUS (02) 9229 5700, NZ: 04 568 8835
**Recommended Use**	Tooth fluoride preparation.
**Other Information**	New Zealand Address: Level 4, 45 Knights Road, Lower Hutt.

2. HAZARDS IDENTIFICATION

**Hazard Classification**	HAZARDOUS SUBSTANCE.<br>DANGEROUS GOODS.<br>Hazard classification according to the criteria of NOHSC.<br>Dangerous goods classification according to the Australia Dangerous Goods Code.
**Risk Phrase(s)**	R11 Highly flammable.<br>R22 Harmful if swallowed.<br>R32 Contact with acids liberates very toxic gas.<br>R43 May cause sensitization by skin contact.
**Safety Phrase(s)**	S16 Keep away from sources of ignition - No smoking.<br>S20/21 When using, do not eat, drink or smoke.<br>S23(2) Do not breathe vapour.<br>S24 Avoid contact with skin.<br>S36/37/39 Wear suitable protective clothing, gloves and eye/face protection.<br>S45 In case of accident or if you feel unwell seek medical advice immediately<br>S7 Keep container tightly closed.

3. COMPOSITION/INFORMATION ON INGREDIENTS

Ingredients

Name	CAS	Proportion
Colophonium	8050-09-7	30-60 %
Ethanol	64-17-5	10-30 %
Sodium Fluoride	7681-49-4	<10 %
Ingredients determined not to be hazardous	Not required	Balance

4. FIRST AID MEASURES

**Inhalation**	If inhaled, remove from contaminated area. Apply artificial respiration if not breathing. Seek medical attention.
**Ingestion**	Do NOT induce vomiting. Immediately wash out mouth with water. Seek medical attention.
**Skin**	Remove all clothing from affected area. Wash skin with running water and mild soap. Wash removed clothing before reuse or discard. If irritation develops, seek medical attention.
**Eye**	If in eyes, hold eyelids apart and flush the eyes continuously with running water. Continue flushing for several minutes until all contaminants are washed off completely. Seek medical attention.
**First Aid Facilities**	Eye wash and normal washroom facilities.
**Advice to Doctor**	Treat symptomatically.

5. FIRE FIGHTING MEASURES

**Suitable Extinguishing Media**	Use water spray, carbon dioxide or dry chemical.

Infosafe No™ LPXPP	Issue Date :October 2008	ISSUED by COLGATE	CS: 1.4.22

Product Name **COLGATE DURAPHAT**

**Hazards from Combustion Products**	Under fire conditions this product may emit toxic and/or irritating fumes including carbon monoxide and carbon dioxide.
**Specific Hazards**	Highly flammable. Vapours will travel considerable distances to sources of ignition.
**Hazchem Code**	3[Y]
**Precautions in connection with Fire**	Fire-fighters should wear full protective clothing and self contained breathing apparatus (SCBA) operated in positive pressure mode. Water spray may be used to keep fire exposed containers cool.

6. ACCIDENTAL RELEASE MEASURES

**Emergency Procedures**	For small spills, clean up with suitable absorbent and wash area with water. For large spills, remove sources of ignition. Evacuate all unprotected personnel. Use proper protective equipment to reduce exposure. Place inert absorbent onto material. Collect the material with non-sparking tools and place into a suitable labelled container. Mop up the remaining material and place into the same container. If large quantities of this material enters the environment, contact the relevant regulatory authorities.

7. HANDLING AND STORAGE

**Precautions for Safe Handling**	Use only in a well ventilated area. Do not mix with acids or oxidising agents. Keep tank covered and containers sealed when not in use. Build up of mists or vapours in the atmosphere must be prevented. Do not use near welding or other ignition sources. Do not inhale vapours. When dealing with large quantities, repeated or prolonged exposure without protection should be prevented. Practice good personal hygiene, that is, always wash hands after handling, and before eating, drinking, smoking or using the toilet facilities.
**Conditions for Safe Storage**	Store in a cool, dry, well ventilated area, away from sources of ignition, acids and oxidising agents. Keep containers closed when not in use, securely sealed and protected against physical damage. Inspect regularly for deficiencies such as damage or leaks. Have appropriate fire extinguishers available in and near the storage area. Take precautions against static electricity discharges. Use proper grounding procedures. Do NOT pressurise, cut, heat or weld empty containers as they may contain hazardous residues.
**Storage Regulations**	For information on the design of the storeroom, reference should be made to Australian Standard AS1940 - The storage and handling of flammable and combustible liquids. Reference should also be made to all Local, State and Federal regulations.

8. EXPOSURE CONTROLS/PERSONAL PROTECTION

National Exposure Standards

No exposure standards have been established for this material, however, the TWA National Occupational Health & Safety Commission (NOHSC) exposure standards for ethanol are as follows;

SUBSTANCE	TWA		STEL	
	ppm	mg/m3	ppm	mg/m3
Ethanol	1000	1800	-	-

TWA (Time Weighted Average): The average airborne concentration of a particular substance when calculated over a normal eight-hour working day, for a five-day week.
STEL (Short Term Exposure Limit): The average airborne concentration over a 15 minute period which should not be exceeded at any time during a normal eight-hour workday.

**Biological Limit Values**	No biological limit available.
**Engineering Controls**	Provide sufficient ventilation. Where vapours or mists are generated, the use of respiratory protection, or a local exhaust ventilation system is recommended.
**Respiratory Protection**	Not necessary under normal conditions of use. However, under industrial conditions, where ventilation is inadequate and vapours or mists are generated the use of a respirator is recommended. Refer AS/NZS 1715 and AS/NZS 1716.
**Eye Protection**	Not required under normal conditions of use. Industrial Applications: The use of safety glasses as appropriate when handling large quantities. Refer to Australian Standard AS/NZS 1337 - Eye Protectors for Industrial Applications.

**Hand Protection**	Impervious gloves should be worn to avoid prolonged skin contact. Reference should be made to AS/NZS 2161.1: Occupational protective gloves - Selection, use and maintenance.
**Body Protection**	Not required under normal conditions of use.<br>Industrial Applications: Suitable impervious protective clothing. Final choice is dependent on individual circumstances.

9. PHYSICAL AND CHEMICAL PROPERTIES

**Appearance**	Straw coloured gel
**Odour**	Not available
**Melting Point**	Not available
**Boiling Point**	Not available
**Solubility in Water**	Soluble
**Specific Gravity**	Not available
**pH Value**	Not available
**Vapour Pressure**	Not available
**Flash Point**	Not available
**Flammability**	Flammable liquid.
**Auto-Ignition Temperature**	Not available
**Flammable Limits - Lower**	Not available
**Flammable Limits - Upper**	Not available

10. STABILITY AND REACTIVITY

**Chemical Stability**	Stable under normal conditions of storage and handling.
**Incompatible Materials**	Strong oxidising agents and acids.
**Hazardous Decomposition Products**	Hazardous decomposition of this product may emit toxic and/or irritating fumes including carbon monoxide and carbon dioxide.
**Hazardous Reactions**	Contact with acids liberates very toxic gas.
**Hazardous Polymerization**	Will not occur.

11. TOXICOLOGICAL INFORMATION

**Toxicology Information**	No toxicity data is available for this material.
**Inhalation**	May cause respiratory tract irritation. Overexposure to high concentrations may result in similar central nervous system effects to those of ingestion ie. headaches, dizziness, drowsiness, nausea, leading to possible unconsciousness and coma.
**Ingestion**	Harmful if swallowed. May cause irritation to the gastrointestinal system. Symptoms cmay include nausea, vomiting and diarrhoea.
**Skin**	May cause irritation in contact with the skin, which may result in redness and itchiness. May cause sensitization by skin contact.
**Eye**	May cause irritation in contact with the eyes, including redness, stinging and lachrymation.
**Chronic Effects**	Prolonged contact may cause sensitization by skin contact.

12. ECOLOGICAL INFORMATION

**Ecotoxicity**	No ecological information available for this product.
**Persistence / Degradability**	Not available

Material Safety Data Sheet

**Mobility**	Not available
**Bioaccumulative Potential**	Not available
**Environ. Protection**	Prevent this material entering waterways, drains or sewers.

13. DISPOSAL CONSIDERATIONS

**Disposal Considerations**	Dispose of in accordance with relevant local, state and federal government regulations.

14. TRANSPORT INFORMATION

**Transport Information**	This material is a Class 3 - Flammable Liquid according to The Australian Code for the Transport of Dangerous Goods by Road and Rail.<br>Class 3 - Flammable Liquids are incompatible in a placard load with any of the following:<br>- Class 1, Explosives<br>- Class 2.1, Flammable Gases, if both the Class 3 and Class 2.1 dangerous goods are in bulk<br>- Class 2.3, Toxic Gases<br>- Class 4.2 Spontaneously Combustible Substances<br>- Class 5.1 Oxidising Agents and Class 5.2, Organic Peroxides<br>- Class 6 Toxic Substances (where the flammable liquid is nitromethane)<br>- Class 7 Radioactive Substances.
**U.N. Number**	1993
**Proper Shipping Name**	FLAMMABLE LIQUID, N.O.S. - Contains Ethanol
**DG Class**	3
**Hazchem Code**	3[Y]
**Packaging Method**	3.8.3RT1
**Packing Group**	III
**EPG Number**	3A1
**IERG Number**	14

15. REGULATORY INFORMATION

**Regulatory Information**	Classified as Hazardous according to criteria of National Occupational Health & Safety Commission (NOHSC).<br>Classified as a Scheduled Poison S4 according to the Standard for the Uniform Scheduling of Drugs and Poisons (SUSDP).
**Poisons Schedule**	S4
**Hazard Category**	Harmful, Irritant, Highly Flammable

16. OTHER INFORMATION

**Date of preparation or last revision of MSDS**	Date MSDS created: October 2008
**Contact Person/Point**	24Hr Emergency Response<br>Australia- 1800 638 556<br>New Zealand- 0800 764 766<br>...End Of MSDS...

© Copyright ACOHS Pty Ltd
Copyright in the source code of the HTML, PDF, XML, XFO and any other electronic files rendered by an Infosafe system for Infosafe MSDS displayed on this site is the intellectual property of Acohs Pty Ltd.
Copyright in the layout, presentation and appearance of each Infosafe MSDS displayed on this site is the intellectual property of Acohs Pty Ltd.
The compilation of MSDS's displayed on this site is the intellectual property of Acohs Pty Ltd.
Copying of any MSDS displayed on this site is permitted for personal use only and otherwise is not permitted. In particular the MSDS's displayed on this site cannot be copied for the purpose of sale or licence or for inclusion as part of a collection of MSDS without the express written consent of Acohs Pty Ltd.

Overview FAQs Information

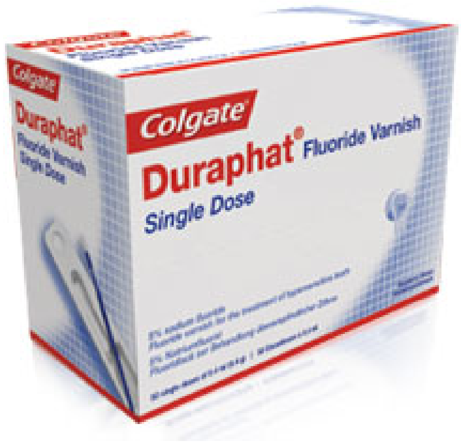

Colgate® Duraphat® Varnish Single Dose
5% Sodium Fluoride (22,600 ppm)

Always read the label. Use only as directed. See your dentist if symptoms persist. For Professional Dental application only

See how it works See list of distributors

Distributors

Australian Dental Professionals - Available from Henry Schein Halas

Colgate Oral Care - Australia	Phone: 1800 262 111<br>Fax: 1800 262 329
Henry Schein Halas - Australia	Phone: 1300 658 822<br>Fax: 1300 658 810

New Zealand Dental Professionals - Available from Henry Schein Shalfoon

Henry Schein Shalfoon - New Zealand<br><br>*Colgate Pro Clinical not available in New Zealand	Phone: 0800 808 855<br>Fax: 0508 808 555
Colgate Oral Care - New Zealand<br><br>*Colgate Pro Clinical not available in New Zealand	Phone: 0508 265 4283<br>Fax: 0508 866 842 7874

High fluoride concentration for dentine hypersensitivity relief

**Product Characteristics**	**Product Benefits**
High Fluoride concentration 22,600ppm F (NaF) for dentine Hypersensitivity relief	High adhesion even on moist surfaces for quick and easy application<br>Good taste for enhanced patient acceptance

Application control and white finish
50 x 0.4ml single doses in a carton

How it Works

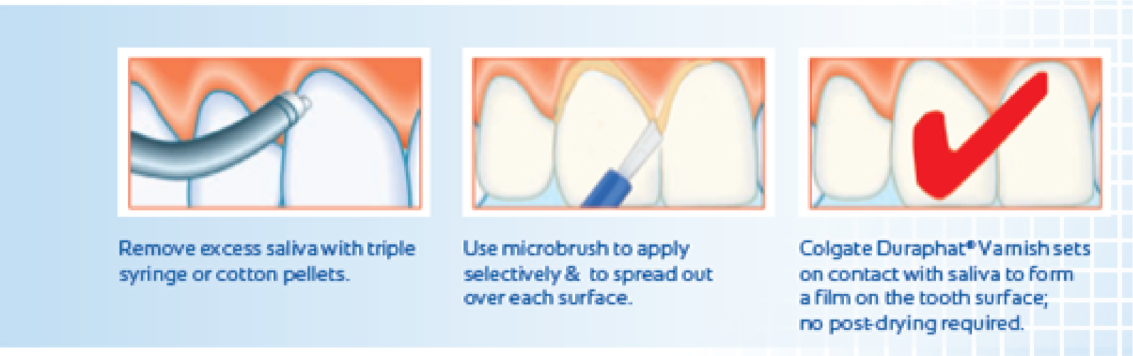

Overview FAQs Information

Colgate® Duraphat® Varnish Single Dose
5% Sodium Fluoride (22,600 ppm)

Always read the label. Use only as directed. See your dentist if symptoms persist. For Professional Dental application only

See how it works See list of distributors

Distributors

**Australian Dental Professionals - Available from Henry Schein Halas**	
Colgate Oral Care - Australia	Phone: 1800 262 111<br>Fax: 1800 262 329
Henry Schein Halas - Australia	Phone: 1300 658 822<br>Fax: 1300 658 810

**New Zealand Dental Professionals - Available from Henry Schein Shalfoon**	
Henry Schein Shalfoon - New Zealand	Phone: 0800 808 855<br>Fax: 0508 808 555
*Colgate Pro Clinical not available in New Zealand	
Colgate Oral Care - New Zealand	Phone: 0508 265 4283<br>Fax: 0508 866 842 7874
*Colgate Pro Clinical not available in New Zealand	

1. Composition

1mL suspension contains 50mg sodium fluoride, equivalent to 22600 ppm F, in an alcoholic solution of resins.

2. Indications

For the treatment of dentine hypersensitivity.

3. Method of Application

Colgate® Duraphat ®Varnish Single Dose is designed to be applied by the dental professional, and is not for self-medication by the patient.

4. Contraindications

Ulcerative gingivitis and stomatitis. Product should not be used in patients allergic to colophony and colophony-derived ingredients.

5. Precautions

Edematous swellings have been reported only in rare instances in some fluoride varnish products, especially after application to extensive surfaces. Nausea has been reported when extensive applications have been made. If required, varnish film can be removed with a thorough brushing. Dyspnea, although extremely rare, has occurred in asthmatic children.

6. Interactions with Other Drugs

When Colgate® Duraphat ® Varnish Single Dose is applied, other fluoride preparations such as fluoride gels should not be administered during the same day.

7. Instructions for Use

Before applying Colgate® Duraphat ®Varnish Single Dose, excess plaque should be removed and the teeth dried. Mix well prior to application. Apply product with supplied brush in the conventional manner. Instruct the patient to avoid solid foods, alcohol, brushing and flossing for 4 hours after application.

8. Presentation

Box contains 50 single doses of 0.4 mL (0.4g), raspberry flavour.

9. Storage

Store at room temperature. Do not use after expiry date printed on the package. Keep out of reach and sight of children

MATERIAL SAFETY DATA SHEET

Professional Disposables International, Inc.

1. Product and Company Identification

**Product Name**	Povidone Iodine Pads and Swabsticks	**MSDS No. 0054**
**CAS #**	Mixture	
**Product use**	Antiseptic	

Manufacturer

Professional Disposables International, Inc.
Two Nice-Pak Park
Orangeburg, NY 10962-1376
or Distributed by:
Professional Disposables International Ltd, Ontario CA
Phone (USA) 1-845-365-1700 (M-F 8am - 5pm)
Phone (CANADA) 1-800-263-7067

LEGEND HMIS/NFPA	
Severe	4
Serious	3
Moderate	2
Slight	1
Minimal	0

Health	*	1
Flammability		0
Physical Hazard		0
Personal Protection		

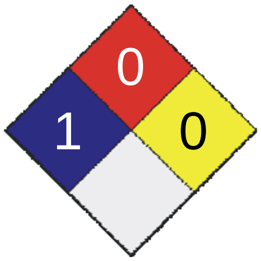

2. Hazards Identification

Emergency overview

CAUTION
CAUSES EYE IRRITATION.
May cause chronic toxic effects.

Potential short term health effects

**Routes of exposure**	Eye, Skin contact, Skin absorption, Inhalation, Ingestion.
**Eyes**	Causes irritation.
**Skin**	In case of skin irritation, discontinue use of the product. Topical application of povidone-iodine elevates serum concentrations of iodine.
**Inhalation**	Not a normal route of exposure. May cause respiratory tract irritation.
**Ingestion**	Not a normal route of exposure. May cause stomach distress, nausea or vomiting.
**Target organs**	Eyes. Skin.
**Chronic effects**	Prolonged or repeated exposure can cause drying, defatting and dermatitis.
**Signs and symptoms**	Symptoms may include redness, edema, drying, defatting and cracking of the skin. Symptoms of overexposure may be headache, dizziness, tiredness, nausea and vomiting.

3. Composition / Information on Ingredients

Ingredient(s)	CAS #	Percent
2-Pyrrolidinone, 1-ethenyl-, homopolymer, compound with iodine	25655-41-8	7 - 13

4. First Aid Measures

First aid procedures

**Eye contact**	Flush with cool water. Remove contact lenses, if applicable, and continue flushing. Obtain medical attention if irritation persists.
**Skin contact**	In case of skin irritation, discontinue use of product.
**Inhalation**	Not a normal route of exposure. If symptoms develop move victim to fresh air. If symptoms persist, obtain medical attention.
**Ingestion**	Not a normal route of exposure. Do not induce vomiting. Never give anything by mouth if victim is unconscious, or is convulsing. Obtain medical attention.
**Notes to physician**	Symptoms may be delayed.

**General advice**	If you feel unwell, seek medical advice (show the label where possible). Ensure that medical personnel are aware of the material(s) involved, and take precautions to protect themselves. Show this safety data sheet to the doctor in attendance. Avoid contact with eyes and skin. Keep out of reach of children. Avoid contact with eyes.

5. Fire Fighting Measures

**Flammable properties**	Not flammable by WHMIS/OSHA criteria.
**Extinguishing media**	
**Suitable extinguishing media**	Treat for surrounding material.
**Unsuitable extinguishing media**	Not available
**Protection of firefighters**	
**Specific hazards arising from the chemical**	Not available
**Protective equipment for firefighters**	Firefighters should wear full protective clothing including self contained breathing apparatus.
**Hazardous combustion products**	May include and are not limited to: Oxides of carbon. Oxides of nitrogen. Iodine.
**Explosion data**	
**Sensitivity to mechanical impact**	Not available
**Sensitivity to static discharge**	Not available

6. Accidental Release Measures

**Personal precautions**	Keep unnecessary personnel away. Do not touch or walk through spilled material. Do not touch damaged containers or spilled material unless wearing appropriate protective clothing. Keep people away from and upwind of spill/leak.
**Methods for containment**	Prevent entry into waterways, sewers, basements or confined areas.
**Methods for cleaning up**	Pick up and discard.

7. Handling and Storage

**Handling**	Use good industrial hygiene practices in handling this material.
**Storage**	Keep out of reach of children. Store in a closed container away from incompatible materials.

8. Exposure Controls / Personal Protection

Exposure limits

**Ingredient(s)**	**Exposure Limits**
2-Pyrrolidinone, 1-ethenyl-, homopolymer, compound with iodine	**ACGIH-TLV**<br>Not established<br>**OSHA-PEL**<br>Not established

**Engineering controls**	General ventilation normally adequate.
**Personal protective equipment**	
**Eye / face protection**	Follow standard industrial hygiene practices.
**Hand protection**	Follow standard industrial hygiene practices.
**Skin and body protection**	As required by employer code.
**Respiratory protection**	Where exposure guideline levels may be exceeded, use an approved NIOSH respirator.
**General hygiene considerations**	Handle in accordance with good industrial hygiene and safety practice. When using do not eat or drink. Wash hands before breaks and immediately after handling the product.

9. Physical & Chemical Properties

**Appearance**	Liquid saturated on pad / Swabstick saturated with liquid
**Color**	Yellow to Dark reddish brown
**Form**	Swabstick saturated with liquid / Liquid saturated on pad

**Odor**	iodine
**Odor threshold**	Not available
**Physical state**	Solid
**pH**	Not available
**Melting point**	Not available
**Freezing point**	Not available
**Boiling point**	97.22 °C (207 °F)
**Flash point**	Not available
**Evaporation rate**	Not available
**Flammability limits in air, lower, % by volume**	Not available
**Flammability limits in air, upper, % by volume**	Not available
**Vapor pressure**	Not available
**Vapor density**	Not available
**Specific gravity**	Not available
**Octanol/water coefficient**	Not available
**Solubility (H2O)**	Pad is not soluble/Stick is not soluble
**Auto-ignition temperature**	Not available
**VOC (Weight %)**	Not available
**Viscosity**	Not available
**Percent volatile**	Not available

10. Chemical Stability & Reactivity Information

**Chemical stability**	Stable under recommended storage conditions.
**Conditions to avoid**	Do not mix with other chemicals.
**Incompatible materials**	Caustics. Oxidizers. Reducing agents.
**Hazardous decomposition products**	May include and are not limited to: Oxides of carbon. Oxides of nitrogen. Iodine.
**Possibility of hazardous reactions**	Hazardous polymerization does not occur.

11. Toxicological Information

Component analysis - LC50

Ingredient(s)	LC50
2-Pyrrolidinone, 1-ethenyl-, homopolymer, compound with iodine	Not available

Component analysis - Oral LD50

Ingredient(s)	LD50
2-Pyrrolidinone, 1-ethenyl-, homopolymer, compound with iodine	8100 mg/kg mouse; 8000.1 mg/kg rat

Effects of acute exposure

**Eye**	Causes irritation.
**Skin**	In case of skin irritation, discontinue use of the product.<br>Topical application of povidone-iodine elevates serum concentrations of iodine.
**Inhalation**	Not a normal route of exposure. May cause respiratory tract irritation.
**Ingestion**	Not a normal route of exposure. May cause stomach distress, nausea or vomiting.
**Sensitization**	Non-hazardous by WHMIS/OSHA criteria.
**Chronic effects**	Chronic ingestion of iodides may produce 'iodism' which is characterized by skin rash, nasal discharge, sneezing, fever, headaches, weakness, anemia and loss of weight.
**Carcinogenicity**	Non-hazardous by WHMIS/OSHA criteria.
**Mutagenicity**	Non-hazardous by WHMIS/OSHA criteria.
**Reproductive effects**	Non-hazardous by WHMIS/OSHA criteria.
**Teratogenicity**	Non-hazardous by WHMIS/OSHA criteria.

12. Ecological Information

**Ecotoxicity**	Not available
**Environmental effects**	Not available
**Aquatic toxicity**	Not available
**Persistence / degradability**	Not available
**Bioaccumulation / accumulation**	Not available
**Partition coefficient**	Not available
**Mobility in environmental media**	Not available
**Chemical fate information**	Not available
**Other adverse effects**	Not available

13. Disposal Considerations

**Waste codes**	Not available
**Disposal instructions**	Discard after single use.<br>Review federal, state/provincial, and local government requirements prior to disposal. Discard with solid waste. Dispose in accordance with all applicable regulations.
**Waste from residues / unused products**	Not available
**Contaminated packaging**	Not available

14. Transport Information

U.S. Department of Transportation (DOT)

Not regulated as dangerous goods.

Transportation of Dangerous Goods (TDG - Canada)

Not regulated as dangerous goods.

15. Regulatory Information

**Canadian federal regulations**	This product has been classified in accordance with the hazard criteria of the Controlled Products Regulations and the MSDS contains all the information required by the Controlled Products Regulations.<br><br>NPN 00489948 - Pad/ NPN 00489964 - Swabstick
**US Federal regulations**	This product is a "Hazardous Chemical" as defined by the OSHA Hazard Communication Standard, 29 CFR 1910.1200 due to the presence of listed sensitizers. All components are on the U.S. EPA TSCA Inventory List.

Occupational Safety and Health Administration (OSHA)

**29 CFR 1910.1200 hazardous chemical**	Yes

CERCLA (Superfund) reportable quantity

Sodium hydroxide: 1000.0000

Superfund Amendments and Reauthorization Act of 1986 (SARA)

**Hazard categories**	Immediate Hazard - Yes<br>Delayed Hazard - Yes<br>Fire Hazard - No<br>Pressure Hazard - No<br>Reactivity Hazard - No
**Section 302 extremely hazardous substance**	No
**Section 311 hazardous chemical**	Yes
**Clean Air Act (CAA)**	Not available
**Clean Water Act (CWA)**	Not available
**Safe Drinking Water Act (SDWA)**	Not available
**Drug Enforcement Agency (DEA)**	Not available
**Food and Drug Administration (FDA)**	Not available

**WHMIS classification**	Exempt - Registered product - (NHP see above)
**State regulations**	This product does not contain a chemical known to the State of California to cause cancer, birth defects or other reproductive harm.

Inventory name

Country(s) or region	Inventory name	On inventory (yes/no)*
Canada	Domestic Substances List (DSL)	Yes
Canada	Non-Domestic Substances List (NDSL)	No
United States & Puerto Rico	Toxic Substances Control Act (TSCA) Inventory	Yes

A "Yes" indicates that all components of this product comply with the inventory requirements administered by the governing country(s)

16. Other Information

**Disclaimer**	Information contained herein was obtained from sources considered technically accurate and reliable. While every effort has been made to ensure full disclosure of product hazards, in some cases data is not available and is so stated. Since conditions of actual product use are beyond control of the supplier, it is assumed that users of this material have been fully trained according to the requirements of all applicable legislation and regulatory instruments. No warranty, expressed or implied, is made and supplier will not be liable for any losses, injuries or consequential damages which may result from the use of or reliance on any information contained in this document.
**Recommended use**	For external use only.
**Issue date**	27-Feb-2009
**Effective date**	01-Mar-2009
**Expiry date**	01-Mar-2012
**Prepared by**	Dell Tech Laboratories Ltd. (519) 858-5021

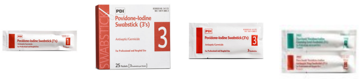

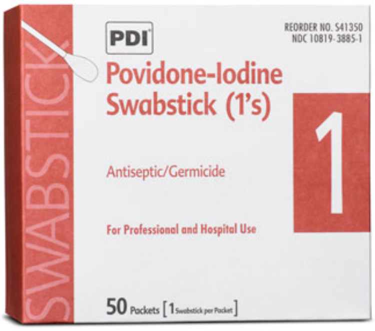

Skin Preparations

PVP Iodine Prep Pads and Swabsticks

OVERVIEW | SIZES & FORMATS | MSDS/SDS & PRODUCT INFO | ACCESSORIES & COMPLIANCE TOOLS

Provides longer germicidal activity than ordinary Iodine Solution. Non-irritating and non-stinging for better patient comfort. Cleansing scrub swabsticks are saturated with a 7.5% Povodine-Iodine cleansing solution to scrub away dead skin, dirt and oil can be more effective. Prep Pads and Swabsticks saturated with a 10% USP Povidone-Iodine prep solution provide proven antisepsis. Duo-Swab contains one cleansing scrub swabstick and one antiseptic swabstick in a convenient 2-step package.

Learn More

How to Buy   Contact Us

Share

Email

© 2014 PDI Healthcare
