# Supplementary material for: Carious lesions in permanent dentitions are reduced in remote Indigenous Australian children taking part in a non-randomised preventive trial
Source: PLoS One. 2021 Jan 28;16(1):e0244927. doi: 10.1371/journal.pone.0244927 (PMC7842954; doi:10.1371/journal.pone.0244927)
Supplement: S2 File — (PDF) [file pone.0244927.s006.pdf]

Queensland Government

HREC/15/QCH/39 – 970
12.06 BJ:jld
Further Information Received
Study Approved

Telephone: *(07) 4226 5513*
Facsimile: *N/A*
Email: Cairns_Ethics@health.qld.gov.au

Far North Queensland
Human Research Ethics Committee

1 June 2015

Email: j.kroon@griffith.edu.au

Prof Newell Johnson
Menzies Health Institute of Queensland
Griffith University
Southport QLD 4222

Dear Prof Johnson

HREC Reference number: HREC/15/QCH/39 - 970
Project title: Effectiveness, cost-effectiveness and cost-benefit of a single annual professional intervention for the prevention of childhood dental caries in a remote rural Indigenous community

Thank you for submitting the above project for ethical and scientific review. This project was first considered by the Far North Queensland Human Research Ethics Committee (FNQ HREC) on 14 May 2015. Your reply to FNQ HREC queries dated 25 May 2015 was received and considered out of session. I am pleased to advise that **final ethical approval** is now granted to your application.

You are reminded that this letter constitutes ethical approval only. You must not commence this research project at a *Queensland Health site, utilize Queensland Health staff or Queensland Health resources* until separate authorisation from the District CEO or Delegate of that site has been obtained.

Thank you for providing a signed HREC Only Form of Indemnity for your non-Queensland Health sites. The document has been escalated through to the CE of Cairns and Hinterland, Hospital and Health Service for signature. Once signed, one original HREC Only Form of Indemnity will be provided back to the Principal of Northern Peninsula Area State College and a copy of the executed document will be sent to you for file.

A copy of this approval letter must be submitted to the Research Governance Officer/Delegated Personnel which govern the Queensland Health site/s listed in your approved NEAF. This letter must be accompanied with a completed Site Specific Assessment (SSA) Form, along with all of your ethically approved documentation for authorisation from the Chief Executive or Delegate of the relevant Health Service.

Send documents to:	**Research Governance Officer – Rosemary Schmidt**
Address:	Level 6, William McCormack Place 2, 5B Sheridan Street, Cairns
Email:	Torres-Cape-Research-Governance@health.qld.gov.au
Phone:	(07) 4226 3033

William McCormack Place
Level 7 East, 5B Sheridan Street
Cairns QLD 4870
PO Box 902 CAIRNS
Queensland 4870 Australia

Queensland Government

HREC/15/QCH/39 – 970
12.06 BJ:jld
Further Information Received
Study Approved

Telephone: *(07) 4226 5513*
Facsimile: *N/A*
Email: Cairns_Ethics@health.qld.gov.au

Far North Queensland
Human Research Ethics Committee

This HREC is constituted and operates in accordance with the National Health and Medical Research Council's (NHMRC) *National Statement on Ethical Conduct in Human Research (2007), NHMRC and Universities Australia Australian Code for the Responsible Conduct of Research (2007)* and the *CPMP/ICH Note for Guidance on Good Clinical Practice*.

The ethically approved documents are:

Document	*Document Version/ Appendix Version*	*Document Date/ Appendix Date*
NEAF Application (AU/1/05FD114)	2.2	27 March 2015
Original Cover Letter	*N/A*	9 April 2015
Reply to HREC Queries Letter	*N/A*	25 May 2015
Information Sheet: Parents/Guardians (clean and tracked)	*3*	25 May 2015
Consent Form (clean and tracked)	*3*	25 May 2015
Information Sheet: Parents/Guardians (clean and tracked)	*3*	25 May 2015
Information Sheet: Students (clean and tracked)	*3*	25 May 2015
2 x HREC Only Form of Indemnity	N/A	N/A
Research Study Protocol	1	9 April 2015
CV Professor Newell Johnson	N/A	*Undated*
CV Ratilal Lallo	N/A	*Undated*
CV Jeroen Kroon	N/A	*Undated*
CV Valda Wallace	N/A	*Undated*
CV Tut K Ohnmar	N/A	*Undated*
CV Sanjeewa Kularatna	N/A	*Undated*
CV Paul Scuffham	N/A	*Undated*
CV Robyn Margaret Boase	N/A	*Undated*
CV Yvonne Cadet-James	N/A	*Undated*
CV Surani Fernando	N/A	*Undated*
Data Collection Form: Northern Peninsula Area (NPA) Child Oral Health Survey	1	9 April 2015
Baseline Questionnaire: Northern Peninsula Area (NPA) Child Oral Health Survey	1	9 April 2015
Letter of Support: Executive General Manager: Torres and Cape Hospital and Health Service	N/A	10 March 2015

William McCormack Place
Level 7 East, 5B Sheridan Street
Cairns QLD 4870
PO Box 902 CAIRNS
Queensland 4870 Australia

Telephone: *(07) 4226 5513*
Facsimile: *N/A*
Email: Cairns_Ethics@health.qld.gov.au

Far North Queensland
Human Research Ethics Committee

Letter of Support: Health Service Chief Executive: Torres Strait Northern Peninsula Hospital and Health Service	N/A	10 January 2014
Letter of Support: Chief Dental Officer: Queensland Government	N/A	6 March 2015
Letter of Support: The Mayor: Northern Peninsula Regional Council	N/A	10 March 2015
Letter of Support: Principal: Northern Peninsula State College	N/A	*Undated*
Letter of Support: Chief Executive Officer: Apunipima Cape York Health Council	N/A	11 March 2014
Funding Approval Letter from NHMRC	N/A	*Undated*
SDI Material Safety Data Sheet for Conseal-Clear, Conseal-Light Grey, Conseal F (White)	N/A	1 August 2012
Product Information Sheet Conseal F	N/A	*Undated*
Material Safety Data Sheet GC Fuji VII / Fuji VII Capsule – Powder	N/A	20 December 2010
Product Information Sheet GC Fuji VII Capsule (pink or white)	N/A	26 March 2015
Material Safety Data Sheet Colgate Duraphat	N/A	October 2008
Product Information Sheet Colgate® Duraphat® Varnish Single Dose 5% Sodium Floride (22,600 ppm)	N/A	*Undated*
Material Safety Data Sheet Povidone Iodine Pads and Swabsticks No. 0054	N/A	27 February 2009
Product Information Sheet PVP iodine Prep Pads and Swabsticks	N/A	26 March 2015

Ethically Approved Queensland Health study sites as per page 14 of your NEAF (submission code AU/1/05FD114):

- Bamaga Hospital
- Bamaga Mobile Dental Clinic

Ethically Approved Non-Queensland Health study sites as per page 14 of your NEAF (submission code AU/1/05FD114) are:

- Northern Peninsula Area (NPA) State College which includes:
  - Injinoo Junior Campus (Pre-prep to Year 7)
  - Bamaga Junior Campus (Pre-prep to Year 7)
  - Bamaga Senior Campus

As noted on page 1, the HREC Only Indemnity Form for your non-Queensland Health sites has been escalated to the CE of Cairns and Hinterland Hospital and Health Service for her signature.

William McCormack Place
Level 7 East, 5B Sheridan Street
Cairns QLD 4870
PO Box 902 CAIRNS
Queensland 4870 Australia

Queensland Government

HREC/15/QCH/39 – 970
12.06 BJ:jld
Further Information Received
Study Approved

Telephone: *(07) 4226 5513*
Facsimile: *N/A*
Email: Cairns_Ethics@health.qld.gov.au

Far North Queensland
Human Research Ethics Committee

(If you are a student researcher you are obliged to advise your Supervisor/s as to the current status of your research application.)

Please note the following conditions of approval:

1. If relevant to your project, your attention is drawn to standards for clinical trials reporting as enunciated in the CONSORT statement *(hhpt://www.consort-statement.ort/?o=1001)* and a requirement by many journals for certain categories of clinical trials to be registered (see: *http://www.anzctr.org.au*/Support/HowToAdd.aspx).

2. The Principal Investigator will immediately report anything which might warrant review of ethical approval of the project in the specified format, including:

    a. Unforeseen events that might affect continued ethical acceptability of the project.
    Serious Adverse Events must be notified to the Committee as soon as possible. In addition the Investigator must provide a summary of the adverse events, in the specified format, including a comment as to suspected causality and whether changes are required to the Patient Information and Consent Form. In the case of Serious Adverse Events occurring at the local site, a full report is required from the Principal Investigator, including duration of treatment and outcome of event.

3. Amendments to the research project which may affect the ongoing ethical acceptability of a project must be submitted to the HREC for review. Major amendments should be reflected in a revised online NEAF (accompanied by all relevant updated documentation and a cover letter from the principal investigator, providing a brief description of the changes, the rationale for the changes, and their implications for the ongoing conduct of the study). Hard copies of the revised NEAF, the cover letter and all relevant updated documents with tracked changes must also be submitted to the HREC coordinator as per standard HREC SOP. Further advice on submitting amendments is available from.

    http://www.health.qld.gov.au/cpic/documents/ethics/researcher_userguide.pdf
4. Amendments to the research project which only affect the ongoing site acceptability of the project are not required to be submitted to the HREC for review. These amendment requests should be submitted directly to the Research Governance Office/r (by-passing the HREC).

5. Proposed amendments to the research project which may affect both the ethical acceptability and site suitability of the project must be submitted firstly to the HREC for review and, once HREC approval has been granted, then submitted to the RGO.

6. Amendments which do not affect either the ethical acceptability or site acceptability of the project(e.g. typographical errors) should be submitted in hard copy to the HREC coordinator. These should include a cover letter from the principal investigator providing a brief description of

William McCormack Place
Level 7 East, 5B Sheridan Street
Cairns QLD 4870
PO Box 902 CAIRNS
Queensland 4870 Australia

HREC/15/QCH/39 – 970
12.06 BJ:jld
Further Information Received
Study Approved

Telephone: *(07) 4226 5513*
Facsimile: *N/A*
Email: *Cairns_Ethics@health.qld.gov.au*

Far North Queensland
Human Research Ethics Committee

the changes and the rationale for the changes, and accompanied by all relevant updated documents with tracked changes.

7. The HREC will be notified, giving reasons, if the project is discontinued at a site before the expected date of completion.

8. The Principal Investigator will provide an annual report to the HREC and at completion of the study in the specified format.

9. The Health Service administration and the Human Research Ethics Committee may inquire into the conduct of any research or purported research, whether approved or not and regardless of the source of funding, being conducted on hospital premises or claiming any association with the Hospital; or which the Committee has approved if conducted outside of Queensland Health however within the jurisdiction of the Committee (Cairns, Cape & Torres Hospital & Health Services).

10. If research is occurring in Indigenous Communities please ensure that you provide progress and feedback to relevant Indigenous Groups.

HREC approval is valid for **three years** from the date of this letter.

Should you have any queries about the HREC's consideration of your project please contact the HREC Administrator on 07 4226 5513. The HREC terms of Reference, Standard Operating Procedures, membership and standard forms are available from:
http://www.health.qld.gov.au/cpic/ethics/reagu_homepage.asp

The HREC wishes you every success in your research.

Yours sincerely

Dr Robert Jones – Acting Chair
Far North Queensland
Human Research Ethics Committee

William McCormack Place
Level 7 East, 5B Sheridan Street
Cairns QLD 4870
PO Box 902 CAIRNS
Queensland 4870 Australia
