# Supplementary material for: Carious lesions in permanent dentitions are reduced in remote Indigenous Australian children taking part in a non-randomised preventive trial
Source: PLoS One. 2021 Jan 28;16(1):e0244927. doi: 10.1371/journal.pone.0244927 (PMC7842954; doi:10.1371/journal.pone.0244927)
Supplement: Attachment — Submitted filename: Review.docx [file pone.0244927.s008.docx]

I would like to thank the authors and the editors for the chance to review this manuscript. Dental caries in high risk, hard to reach populations is a critical research area and one that this reviewer has particular interest in. As such, rigorous research is a welcome contribution.

The authors report on a non-randomized study of the combined effects of fluoride varnish and pit/fissure sealants for the prevention of dental caries in indigenous populations. The individual effectiveness of these agents in the prevention of caries is well known and has been thoroughly studied, as well as being formally recommended by multiple national agents for this purpose. The authors argue that the combined effects of these agents have not been evaluated, but I believe there are studies currently in operation that have still been studied. Some have investigated this combination both in clinical and pragmatic studies, though in varying forms (e.g., 10.1186/s12903- 018-0514-6; 10.1002/14651858.CD003067.pub4; 10.5005/jp-journals-10005-1491; 10.1016/j.adaj.2017.05.028), which may be useful to cite in the literature of the study. This reviewer knows also of two ongoing clinical trials looking at the impact of the combination of therapies, one arm of which is FV+sealants.

That said, there is still need for further evidence of the combined effects of these agents in high-risk alternative populations. Thus, the overall scientific contribution from this type of research can be positive. The authors do a wonderful job of presenting their study in detail. However, there are substantial methodological concerns as described below that weakened enthusiasm for this manuscript.

This reviewer disagrees with non-consenting children being considered a “natural” comparison group. They can be treated as a comparator but this language is, I believe, too confusing and similar to that of natural experiments. Further, I was unable to find a comparison (baseline or otherwise) between the consenting and non-consenting children. The obvious concern is that there are systematic differences in patients that consent and do not consent to the study. In fact, there seem to be no tables or results anywhere regarding the sociodemographic breakdown of the participants in either group. There is a sentence in results saying they “largely matched”, but there is no table and it needs to be shown.

The outcome of caries incidence is complicated by exfoliation. The age group on the low end means there may have been instances wherein primary teeth were exfoliated during the study and should this data have been carious, it is biased.

I am confused by a sentence in results about extraction due to caries, I thought this was a program due to a lack of care access for the patient population (typical for this type of intervention). Yet children with carious lesions have access to extractions? Could the authors clarify?

Post hoc sample size calculation for power is generally not recommended.

As the intervention was not randomized and the children effectively self-selected into the study intervention and comparator groups, it is unclear if the effect is true. E.g., if consenting children have greater wealth (as a simple example) or more attentive parents, the home environment may be more conducive to a healthier diet and/or hygiene behavior. This is probably the most problematic limitation.

Please justify the MAR assumption.

The MW test is a fairly basic nonparametric test. Given the non-randomization of the design this can lead to serious concerns over confounding, and the authors did not seem to do any adjustment (e.g., propensity score matching or inverse probability weighting) to alleviate this concern.

The reviewer cannot find tables on the baseline measures for caries between groups.

There are a number of MWU tests performed here, yet no treatment or discussion is given to the possibly large experiment wise error rate. The authors should consider nonparametric regression or multivariate regression of transformed outcomes, which would also allow them to consider the varying sociodemographic variables in the model.

ITT and PPA was conducted, and the authors went with PPA, yet no justification is given.

Results: please provide confidence intervals. The authors note the non-normality of outcome data yet did not describe the severity of this departure. Given the MW test, the authors could use a Wilcoxon test CI for medians or log-transform their data and calculate a modified Cox confidence interval. These will be more useful than with P-values.

The authors should be careful of the use of “effective” and “effectiveness” given the nature of the design of the study.

In the Discussion, it is confusing to read a paragraph about SSBs. This reviewer understands that SSBs are a significant contributor to caries, but the authors’ reversal here to focus on social determinants when the paper was focused on care provision seems out of place.

One of the concerns in this report is the authors’ own admission that while sealants can be a “set it and forget it” intervention, general clinical recommendations include the multiple reapplication of fluoride varnish. The authors admit, understandably, that this is infeasible for the specific population, however this creates an issue with the interpretation of the results. It is unable to be identified whether the major preventive effect on the observed incidence of caries is due to the longer-lasting sealants, an atypical response to a single application of FV, or a combination of both. Ideally, this study would have had additional treatment groups for just FV and just sealants, but this absence means there is a notable limitation in the impact of the paper.

Overall, despite the nice design and excellent presentation of the manuscript, the methodological limitations lead the reviewer to believe it is not of a high enough standard for publication in PLOS One. As a non-randomized trial, one would expect to see more rigorous treatment of internal and external validity threats in analysis, but the (over)use of MWU tests was considered weak. Additionally, the self-selection of participants into treatment and control was also considered a major limitation.

Criteria notes:

- The results are original research and have not been published elsewhere.
- Overall, the article is intelligible and well-written.
- The study provided acceptable ethical approval
- The paper follows standard reporting guidelines
