# Supplementary material for: Carious lesions in permanent dentitions are reduced in remote Indigenous Australian children taking part in a non-randomised preventive trial
Source: PLoS One. 2021 Jan 28;16(1):e0244927. doi: 10.1371/journal.pone.0244927 (PMC7842954; doi:10.1371/journal.pone.0244927)
Supplement: Attachment — Submitted filename: Response_to_reviewers_PloSOne_SKT&NWJ.070220 (2).docx [file pone.0244927.s009.docx]

| **Reviewer 1:** | **Responses:** |
| --- | --- |
| The manuscript addresses an interesting topic. The research questions are well-posed and the data contain a rich set of information. The employed statistical methods are very basic and not all the data features have been accounted properly. Some comments follow. | The full statistical analysis has been re-worked, based on detailed comments from both reviewers. Additional details of the changes are provided with the relevant comments.  In summary the statistical analysis now focused on the 2-year follow-up, and the 1-year follow-up findings have been removed. The all surface (combination of two dentitions) have also been removed to avoid confusion.  We have also removed the comparison of experimental group children who received one and two preventive intervention over the study period as this is also possibly confusing but also adds little value to the overall analyses conducted.  We have now included the baseline comparison findings for the two groups.  We have reported Medians (95% Confidence intervals) instead of Means (SDs).  We have conducted and reported the per-protocol multivariate analysis to assess the effect of the intervention after adjusting for potential confounders, focusing on the important outcome (as suggested by reviewer 3) of advanced caries increment in the permanent dentition.  We have replicated the per-protocol analysis on Intention to Treat (ITT) basis for both bivariate and multivariate analyses. |
| The data are not fully available. This is not in line with the journal's guidelines. Moreover, this does not allow the reviewer to reproduce the results. This is particularly important as several sentence must be verified, e.g. the fact the mathcing is properly performed,"largely matched the characteristics of the comparison group" is quite a strong sentence if not supported by any evidence. | Access to raw data will be considered on request. Because of cultural sensitivities these will be referred to the Griffith University Research Ethics Committee and the Community will be contacted for advice.  A detailed table presenting the baseline comparison of the two groups is now included with supporting text in the Results section. |
| As missing data arise, more details on the multiple imputation procedure should be provided, pointing out the potential bias and/or increase in the uncertainty (e.g. standard errors of the estimates) due to the imputation approach. As a general matter, an analysis of the imputed data should be provided. It is important to inspect the imputations. In general, a good imputed value is a value that could have been observed had it not been missing. | Data from the imputed analysis are now included in the revised manuscript. Standard errors are presented in the multivariate analysis tables. |
| A major concern refers to the analysis of the main outcome. The ICDAS classification leads to a categorical ordered variable. The use of a non-parametric test is sound, though reporting the "mean" is rather misleading. Moreover, the data are rich of information. A longitudinal data structure is available and completely neglected. A mixed effects model must be employed to better appreciate the differences between the groups, taking into account possible individual-specific confounders and a possible trend/growth effect. Heterogeneity may be quite an issue, in particular in small/medium sample size analyses as few observation may strongly affect inferential procedures. | The outcome in this study was the incidence of new lesions, i.e., the count of incipient and advanced caries which developed on tooth surfaces that were caries free at baseline. Means were reported as it is easy to interpret. We have now reported medians with 95% CIs, as recommended.  We have discussed conducting a mixed effects model with our resident statistics Professor who had some reservations in relation to use of mixed effects model on this data where the outcome is incidence (new lesions). A mixed effects model would undermine the use of valid incidence data by only considering the prevalence of dental caries at each timepoint. We have now conducted a negative binomial regression with log link and have taken into account potential confounders including baseline caries as suggested. |
| As a minor point, it is rather unclear why the Permanent surface and All surfaces values differ as they are computed on the same number of subject, with the All surfaces values that are often greater than the others. | Apologies, if we did not present this adequately. Yes, all surface values are greater than permanent surfaces as ‘all surfaces” is a sum of both deciduous and permanent surfaces. The mean of ‘all surfaces’ is dependent on deciduous and permanent surfaces but is not the total sum as the number of individuals with deciduous and permanent dentitions is not the same.  We have however removed the all surface findings to avoid any confusion. |
|  |  |
| **Reviewer 2:** | **Responses:** |
| I would like to thank the authors and the editors for the chance to review this manuscript. Dental caries in high risk, hard to reach populations is a critical research area and one that this reviewer has particular interest in. As such, rigorous research is a welcome contribution. | We thank the referee for his understanding and support |
| The authors report on a non-randomized study of the combined effects of fluoride varnish and pit/fissure sealants for the prevention of dental caries in indigenous populations. The individual effectiveness of these agents in the prevention of caries is well known and has been thoroughly studied, as well as being formally recommended by multiple national agents for this purpose. The authors argue that the combined effects of these agents have not been evaluated, but I believe there are studies currently in operation that have still been studied. Some have investigated this combination both in clinical and pragmatic studies, though in varying forms (e.g., 10.1186/s12903- 018-0514-6; 10.1002/14651858.CD003067.pub4; 10.5005/jp-journals-10005-1491; 10.1016/j.adaj.2017.05.028), which may be useful to cite in the literature of the study. This reviewer knows also of two ongoing clinical trials looking at the impact of the combination of therapies, one arm of which is FV+sealants. | The Introduction has been updated to include the suggested research evidence. |
| That said, there is still need for further evidence of the combined effects of these agents in high-risk alternative populations. Thus, the overall scientific contribution from this type of research can be positive. The authors do a wonderful job of presenting their study in detail. However, there are substantial methodological concerns as described below that weakened enthusiasm for this manuscript. |  |
| This reviewer disagrees with non-consenting children being considered a “natural” comparison group. They can be treated as a comparator but this language is, I believe, too confusing and similar to that of natural experiments. Further, I was unable to find a comparison (baseline or otherwise) between the consenting and non-consenting children. The obvious concern is that there are systematic differences in patients that consent and do not consent to the study. In fact, there seem to be no tables or results anywhere regarding the sociodemographic breakdown of the participants in either group. There is a sentence in results saying they “largely matched”, but there is no table and it needs to be shown. | A detailed table presenting the baseline comparison of the two groups is now included with supporting text in the Results section. |
| The outcome of caries incidence is complicated by exfoliation. The age group on the low end means there may have been instances wherein primary teeth were exfoliated during the study and should this data have been carious, it is biased. | When caries incidence was computed only those missing due to caries were considered. i.e., we used ICDAS code 97. Examiners inquired if the tooth was missing due to caries. However, it is possible that inaccuracies can arise if deciduous teeth developed caries but exfoliate and are replaced by a permanent tooth between the examination time points. We have now included a statement on this as a limitation. |
| I am confused by a sentence in results about extraction due to caries, I thought this was a program due to a lack of care access for the patient population (typical for this type of intervention). Yet children with carious lesions have access to extractions? Could the authors clarify? | There is a public dental service available to the community, albeit limited, in the community and a distant island, only accessible by ferry. These services are however essentially for adults in the community, although children are treated, usually in emergencies. The number of teeth extracted during the study period should be small and not impact on the findings of the study. An accurate account of services received outside of the study would require a complex and lengthy (up to six to 12 months) approval process to receive. There is no private dental service in this community or nearby. |
| Post hoc sample size calculation for power is generally not recommended. | Thanks for recommendation, we have deleted this information. |
| As the intervention was not randomized and the children effectively self-selected into the study intervention and comparator groups, it is unclear if the effect is true. E.g., if consenting children have greater wealth (as a simple example) or more attentive parents, the home environment may be more conducive to a healthier diet and/or hygiene behavior. This is probably the most problematic limitation. | A detailed table presenting the baseline comparison of the two groups is now included with supporting text in the Results section. |
| Please justify the MAR assumption. | We performed Littles MCAR test with the p-values being significant for all the variables with the missing data. With the p-values being significant, the missing data could not be MCAR. We also compared the baseline characteristics between the dropouts and those completing the experiment to assess the dropout bias. There was no dropout bias and we cannot conceptualize any unobserved variables that could predict the missingness of the data in this population, particularly the outcome data. Therefore, MNAR is out of question.  This leaves us with the assumption that the data is MAR. With the assumption of MAR, we have included all the variables that could be theoretically predictive of missing data while imputing the missing data as can be seen in the description in the statistical analyses. This is commonly accepted practice.  [Sterne JA](https://www.ncbi.nlm.nih.gov/pubmed/?term=Sterne%20JA%5BAuthor%5D&cauthor=true&cauthor_uid=19564179), [White IR](https://www.ncbi.nlm.nih.gov/pubmed/?term=White%20IR%5BAuthor%5D&cauthor=true&cauthor_uid=19564179), [Carlin JB](https://www.ncbi.nlm.nih.gov/pubmed/?term=Carlin%20JB%5BAuthor%5D&cauthor=true&cauthor_uid=19564179), [Spratt M](https://www.ncbi.nlm.nih.gov/pubmed/?term=Spratt%20M%5BAuthor%5D&cauthor=true&cauthor_uid=19564179), [Royston P](https://www.ncbi.nlm.nih.gov/pubmed/?term=Royston%20P%5BAuthor%5D&cauthor=true&cauthor_uid=19564179), [Kenward MG](https://www.ncbi.nlm.nih.gov/pubmed/?term=Kenward%20MG%5BAuthor%5D&cauthor=true&cauthor_uid=19564179), [Wood AM](https://www.ncbi.nlm.nih.gov/pubmed/?term=Wood%20AM%5BAuthor%5D&cauthor=true&cauthor_uid=19564179), [Carpenter JR](https://www.ncbi.nlm.nih.gov/pubmed/?term=Carpenter%20JR%5BAuthor%5D&cauthor=true&cauthor_uid=19564179). Multiple imputation for missing data in epidemiological and clinical research: potential and pitfalls. [BMJ.](https://www.ncbi.nlm.nih.gov/pubmed/19564179) 2009 Jun 29;338:b2393. doi: 10.1136/bmj.b2393.  <https://www.ncbi.nlm.nih.gov/pubmed/19564179>  [Dong Y](https://www.ncbi.nlm.nih.gov/pubmed/?term=Dong%20Y%5BAuthor%5D&cauthor=true&cauthor_uid=23853744), [Peng CY](https://www.ncbi.nlm.nih.gov/pubmed/?term=Peng%20CY%5BAuthor%5D&cauthor=true&cauthor_uid=23853744). Principled missing data methods for researchers. [Springerplus.](https://www.ncbi.nlm.nih.gov/pubmed/23853744) 2013 May 14;2(1):222. doi: 10.1186/2193-1801-2-222.  <https://www.ncbi.nlm.nih.gov/pubmed/23853744> |
| The MW test is a fairly basic nonparametric test. Given the non-randomization of the design this can lead to serious concerns over confounding, and the authors did not seem to do any adjustment (e.g., propensity score matching or inverse probability weighting) to alleviate this concern. | There is a plethora of literature supporting the use of regression analysis controlling the effect of confounding variables over matching. Also, matching is not as sensitive to the functional form of covariates as in regression. Therefore, we used multivariate regression to control the confounding.  Hettle R, Corbett M, Hinde S, et al. The assessment and appraisal of regenerative medicines and cell therapy products: an exploration of methods for review, economic evaluation and appraisal.  <https://www.ncbi.nlm.nih.gov/books/NBK424728/>  Brazauskas R, Logan BR. [Observational Studies: Matching or Regression?](https://www.ncbi.nlm.nih.gov/pubmed/26712591) Biol Blood Marrow Transplant. 2016 Mar;22(3):557-63. doi: 10.1016/j.bbmt.2015.12.005.  <https://www.ncbi.nlm.nih.gov/pmc/articles/PMC4756459/>  Alemayehu D, Alvir JM, Jones B, Willke RJ. Statistical issues with the analysis of nonrandomized studies in comparative effectiveness research. J Manag Care Pharm. 2011 Nov-Dec;17(9 Suppl A):S22-6.  <https://www.ncbi.nlm.nih.gov/pubmed/22074671>  We have included the results from negative binomial regression analysis with log link. Adjustments were made for potential confounding variables including baseline caries experience. |
| The reviewer cannot find tables on the baseline measures for caries between groups. | A detailed table presenting the baseline comparison of the two groups is now included with supporting text in the Results section. |
| There are a number of MWU tests performed here, yet no treatment or discussion is given to the possibly large experiment wise error rate. The authors should consider nonparametric regression or multivariate regression of transformed outcomes, which would also allow them to consider the varying sociodemographic variables in the model. | Thanks for the suggestion. We have conducted a negative binomial regression analysis with log link with caries increment as the outcome variable. |
| ITT and PPA was conducted, and the authors went with PPA, yet no justification is given. | Due to the analyses being similar and due to space limitation, we opted to only present the PPA findings in the original submission. However, we have now presented both the PPA and ITT findings for the bivariate and multivariate analyses. |
| Results: please provide confidence intervals. The authors note the non-normality of outcome data yet did not describe the severity of this departure. Given the MW test, the authors could use a Wilcoxon test CI for medians or log-transform their data and calculate a modified Cox confidence interval. These will be more useful than with P-values. | We have now provided medians with 95% CIs. |
| The authors should be careful of the use of “effective” and “effectiveness” given the nature of the design of the study. | We have used the word “efficacious” where appropriate. |
| In the Discussion, it is confusing to read a paragraph about SSBs. This reviewer understands that SSBs are a significant contributor to caries, but the authors’ reversal here to focus on social determinants when the paper was focused on care provision seems out of place. | This is a small component of the Discussion, and we believe that emphasizing addressing the social determinants of dental caries is important; and have therefore retained this in the Discussion. |
| One of the concerns in this report is the authors’ own admission that while sealants can be a “set it and forget it” intervention, general clinical recommendations include the multiple reapplication of fluoride varnish. The authors admit, understandably, that this is infeasible for the specific population, however this creates an issue with the interpretation of the results. It is unable to be identified whether the major preventive effect on the observed incidence of caries is due to the longer-lasting sealants, an atypical response to a single application of FV, or a combination of both. Ideally, this study would have had additional treatment groups for just FV and just sealants, but this absence means there is a notable limitation in the impact of the paper. | It is not ethical to create another group for just FV and FS when it is evident that both are effective. The study would probably not have been funded and receive ethics approval if such a design was planned. This study design was planned with consideration to the sensitivities for conducting research in a remote Indigenous community. We have added:  “ it must be emphasized that the purpose of the study was to determine the efficacy, and cost effectiveness, of professional interventions known to be effective individually, but deliberately applied together for maximum effect. The study is essentially a piece of health economics research: it was not designed to evaluate disinfectants, FV and sealants individually”. |
| Overall, despite the nice design and excellent presentation of the manuscript, the methodological limitations lead the reviewer to believe it is not of a high enough standard for publication in PLOS One. As a non-randomized trial, one would expect to see more rigorous treatment of internal and external validity threats in analysis, but the (over)use of MWU tests was considered weak. Additionally, the self-selection of participants into treatment and control was also considered a major limitation. | We have re-worked the full statistical analysis. Self-selection of participants is tied with the ethical approval and consent processes of the study.  Research in remote Indigenous communities is challenging. Whilst we had strong support from Elders and local school and health care personnel, it is difficult to reach and engage every single family. A degree of pragmatism is required. We are convinced that we have made a positive impact on the health and wellbeing of these children and this is supported by our econ omics evaluation, currently in press.  HQLO-D-19-00484R1 Demonstration of high value care to improve oral health of a remote Indigenous community in Australia Sanjeewa Kularatna, PhD; Ratilal Lalloo, PhD; Jeroen Kroon; Santosh K K Tadakamadla, PhD; Paul A scuffham, PhD; Newell W Johnson, PhD Health and Quality of Life Outcomes |
| Criteria notes:   - The results are original research and have not been published elsewhere. - Overall, the article is intelligible and well-written. - The study provided acceptable ethical approval - The paper follows standard reporting guidelines |  |
|  |  |
| **Reviewer 3:** | **Responses:** |
| I would like to congratulate the authors and declare that I am very thankful for the opportunity to revise this manuscript. It is well designed, conducted and reported. This topic is extremely important to improve the public health politic for disadvantaged populations. However, some points should be improved: |  |
| Previous studies have showed that initial noncavitated caries lesion have no higher risk of developing a severe increment in caries. Studies have also showed that many of these lesion arrest without a interevention by dentist. In this cases, the preventive politics should be considered in order to avoid frank dentin cavitation. Thus, the primary outcome should be focused in more severe caries lesion. Discussion should also be focused in results about moderate-advanced caries lesions. Well OK, and the res.t............ | We do separate incipient and advance caries increment in the analysis. The multivariate model only analyses advances caries increment in the permanent dentition. |
| Furtheremore, the conclusion should be based only in the results. Speculations or ideas should be added in discussion section. | The conclusion has been edited to focus on the findings, and speculations and ideas have been moved to the Discussion. |
