# Supplementary material for: Carious lesions in permanent dentitions are reduced in remote Indigenous Australian children taking part in a non-randomised preventive trial
Source: PLoS One. 2021 Jan 28;16(1):e0244927. doi: 10.1371/journal.pone.0244927 (PMC7842954; doi:10.1371/journal.pone.0244927)
Supplement: Attachment — Submitted filename: Review2.docx [file pone.0244927.s010.docx]

In the introduction, the authors again used “efficacy” in one sentence and “effectiveness” in another. These are different concepts and, one could argue, both incorrect for the study as presented. Later on they say “impact”, when really all they can say in this study is *association*.

The nonconsenting children *do not* constitute a “natural comparison group”. This needs to be changed, and was mentioned in my previous review.

It is a bit of a misnomer to call the treatment group the “experimental group”. It not as egregious as natural comparison but they should consider a different name. This is not an RCT.

It makes sense that caries increment is not normally distributed if it is a count variable.

This reviewer does not agree with the authors responses regarding their approach to confounding. First and foremost, I believe they are conflating typical regression adjustment for confounding with causal effects, particularly in their included citation of regression versus matching. Respectfully, that is not a statistical paper. Matching is meant to try to eliminate the potential selection bias by identifying all possible variables associated with the probability of treatment assignment, given the non-random assignment of the participants of the study. I would suggest the reviewers review Morgan & Winship, “Counterfactuals and Causal Inference”, as well as Hernan and Robins, “Causal Inference”, for a thorough discussion regarding—and comparison of—matching, regression adjustment, and weighting in observational research. There are interesting chapters on each that would greatly help the authors in constructing the rationale for their methods and for conducting the analyses. I believe you will find, generally, that when simply using covariate adjustment the results are not as robust as another method (e.g., IPW-MSM, PSM, IV estimation, etc.)

As a minor comment, *multivariate* regression is multiple outcomes. The authors more likely mean multiple regression (or less commonly, multivariable but that tends to only lead to greater confusion). This needs to be changed.

While there is now a table 1 included in the manuscript, this is of course just the distribution of *observable characteristics*, and the potential flaw of any cross-sectional (or observational study in general) is that unobservable characteristics still confound the relationship in your study.

Table 2 needs to have the 2-year comparison in the title

If I understand correctly, at baseline there were differences in teeth with incipient caries between treatment and control groups for permanent dentition and advanced caries for deciduous teeth. The authors then report significant differences at the end of the second follow-up in incipient caries increment of permanent surfaces. It does not seem that they did (or were able) to control for baseline differences. This would mean (again, assuming I read correctly) that the results in Table 2 are invalid.

Table 3 is not multivariate, but multiple.

In table 3, is the baseline caries experience predictor the total surfaces with caries? I would not think so, but then I wonder why the baseline caries surfaces from Table 1 (incipient and total) are not included in the model.

Regarding table 3, if the authors are saying 2^nd^ follow-up, then that implies they have baseline, first follow-up, and second follow-up. While I see that they included in their response to reviewer 1 that their resident statistician nixed the mixed model, you can in fact incorporate a GEE analysis for incidence or similar method to use all available data. This reviewer agrees with Reviewer 1, if you have longitudinal data, use it!

In the discussion, I would argue that the design of the study does not allow you to prove anything, only conclude associations.
