# Supplementary material for: Carious lesions in permanent dentitions are reduced in remote Indigenous Australian children taking part in a non-randomised preventive trial
Source: PLoS One. 2021 Jan 28;16(1):e0244927. doi: 10.1371/journal.pone.0244927 (PMC7842954; doi:10.1371/journal.pone.0244927)
Supplement: Attachment — Submitted filename: PLOS_One_review_responses_10.9.20.docx [file pone.0244927.s011.docx]

Our sincerest thank you to all the reviewers for their comments on our resubmission. Below we summarise our responses and highlight in yellow a selection of the additions we have made in the manuscript. Further additions are also highlighted in yellow in the revised manuscript.

***Reviewer 1:***

**Comment**: I have two minor points to be further discussed.
a. Some sensitivity analysis about the imputation data used must be included. How many imputed datasets did you consider? Of course, several must be considered.

**Response**: This is included in the methods under the statistical analysis section, lines 157-158 on page 8 - ten iterations were used, and five imputed datasets were obtained.

**Comment**: b. I appreciate the use of the negative binomial regression model. I feel that as you are modelling the new lesions, the inclusion of random effects is necessary to account for the longitudinal strucutre of the data. The book from Rainer Winkelmann "Econometric Analysis of Count Data" could be a good reference to get why random effects may be used. Of course, please provide the results of the likelihood ratio test to check if the random effects are needed or not.

**Response**: Thank you for your suggestion. To account for the repeated measures nature of the data we have adjusted all regression models for caries experience at baseline. This has the effect on including both measurements of the outcome under investigation in the same model.

Lines 144-153, page 7:

“The association between group and caries increment was investigated using negative binomial regression models. In the initial model, group was included as the main effect and baseline caries experience as the covariable. Multivariable analyses with full adjustment for all covariables were conducted for the caries outcomes that demonstrated significant associations with group in the initial model. Covariables included in the adjusted models were age, sex, baseline caries experience, soft drink, lolly, syrup/jam, sugar consumption, salivary mutans streptococci and salivary LB levels. The negative binomial model was chosen due to the overdispersion of the outcome data, and the consequent inappropriateness of a Poisson model for this count data. Effect estimates are presented as Incidence Rate Ratios (IRR) with 95% confidence intervals (CIs).”

***Reviewer 2:***

**Comment**: In the introduction, the authors again used “efficacy” in one sentence and “effectiveness” in another. These are different concepts and, one could argue, both incorrect for the study as presented. Later on they say “impact”, when really all they can say in this study is *association*.

**Response**: In the Introduction we have replaced ‘efficacy’ with association and “effectiveness” with impact. Effectiveness appears elsewhere in the manuscript, but these relate to the evidence reviewed.

**Comment**: The nonconsenting children *do not* constitute a “natural comparison group”. This needs to be changed, and was mentioned in my previous review.

**Response**: The word natural has been removed, the non-intervention group is now referred to as the Comparison group.

**Comment**: It is a bit of a misnomer to call the treatment group the “experimental group”. It not as egregious as natural comparison but they should consider a different name. This is not an RCT.

**Response**: We have changed the name of the “experimental group” to the Intervention group.

**Comment**: This reviewer does not agree with the authors responses regarding their approach to confounding. First and foremost, I believe they are conflating typical regression adjustment for confounding with causal effects, particularly in their included citation of regression versus matching. Respectfully, that is not a statistical paper. Matching is meant to try to eliminate the potential selection bias by identifying all possible variables associated with the probability of treatment assignment, given the non-random assignment of the participants of the study. I would suggest the reviewers review Morgan & Winship, “Counterfactuals and Causal Inference”, as well as Hernan and Robins, “Causal Inference”, for a thorough discussion regarding—and comparison of—matching, regression adjustment, and weighting in observational research. There are interesting chapters on each that would greatly help the authors in constructing the rationale for their methods and for conducting the analyses. I believe you will find, generally, that when simply using covariate adjustment the results are not as robust as another method (e.g., IPW-MSM, PSM, IV estimation, etc.)

**Response**: Following the reviewer’s suggestions we have refined the language in this manuscript to emphasize that we are measuring the association between children who received the intervention and children who did not in terms of their caries experience, and are not attempting to make any claims regarding causal effects. For key outcomes we have presented both unadjusted effect estimates, and also effect estimates adjusted for a number of potentially confounding variables. We have interpreted the resulting effect estimate (in this case an incidence rate ratio) in the standard manner – it represents the difference in incidence rates between the intervention and comparison groups when all covariables in the model are held constant. That is, it is a measure of the association between the intervention and comparison groups after adjusting for a string of potentially confounding variables

**Comment**: As a minor comment, *multivariate* regression is multiple outcomes. The authors more likely mean multiple regression (or less commonly, multivariable but that tends to only lead to greater confusion). This needs to be changed.

**Response***:* Thank you. We agree and have changed to the more correct term “multivariable”.

**Comment**: While there is now a table 1 included in the manuscript, this is of course just the distribution of *observable characteristics*, and the potential flaw of any cross-sectional (or observational study in general) is that unobservable characteristics still confound the relationship in your study.

**Response**: In any observational study there are a large number of unmeasured characteristics. These characteristics may, or may not, be balanced between groups (of course, without measuring them we cannot know if they are balanced or not). It is important to note that the association between groups and caries remained of the same magnitude after adjusting for potentially confounding variables, and that these variables included demographic characteristics, sugar consumption behavior, and baseline caries experience. Extensive literature reviews inform us that these are the key variables that need to be accounted for when adjusting regression models. Consequently, while it is possible unobserved confounders exist, we believe it is unlikely there are other unmeasured confounders that would significantly alter the magnitude of the associations reported in this manuscript.

**Comment**: Table 2 needs to have the 2-year comparison in the title

**Response**: Revised - Table 2: Association between group and caries increment at 2-year follow-up: per-protocol analysis.

**Comment:** If I understand correctly, at baseline there were differences in teeth with incipient caries between treatment and control groups for permanent dentition and advanced caries for deciduous teeth. The authors then report significant differences at the end of the second follow-up in incipient caries increment of permanent surfaces. It does not seem that they did (or were able) to control for baseline differences. This would mean (again, assuming I read correctly) that the results in Table 2 are invalid.

**Response:** It is true that there were significant differences between the intervention and comparison groups for incipient caries in the permanent dentition and advanced caries in the deciduous dentition. As suggested, we have therefore now conducted negative binomial regressions adjusted for ‘baseline caries experience’ for results in table 2 followed by a fully adjusted multivariable analysis in table 3. Also, it is to be noted that advanced caries in the permanent dentition was the only outcome that was significantly associated with the group allocation as can be seen in table 2. Therefore, a fully adjusted multivariable analysis was only conducted for this outcome as can be seen in table 3.

Lines 144-153, page 7:

The association between group and caries increment was investigated using negative binomial regression models. In the initial model, group was included as the main effect and baseline caries experience as the covariable. Multivariable analyses with full adjustment for all covariables were conducted for the caries outcomes that demonstrated significant associations with group in the initial model. Covariables included in the adjusted models were age, sex, baseline caries experience, soft drink, lolly, syrup/jam, sugar consumption, salivary mutans streptococci and salivary LB levels. The negative binomial model was chosen due to the overdispersion of the outcome data, and the consequent inappropriateness of a Poisson model for this count data. Effect estimates are presented as Incidence Rate Ratios (IRR) with 95% confidence intervals (CIs).

**Comment**: Table 3 is not multivariate, but multiple.

**Response**: Thank you for the suggestion. This is corrected as ‘multivariable’.

**Comment**: In table 3, is the baseline caries experience predictor the total surfaces with caries? I would not think so, but then I wonder why the baseline caries surfaces from Table 1 (incipient and total) are not included in the model.

**Response:** In tables 2 and 3, the variable ‘baseline caries experience’ relates to baseline caries on a specific surface and dentition conforming to the dentition and extent of caries in the outcome. For instance: when the outcome is advanced caries increment in permanent dentition, the variable baseline caries experience would only include number of surfaces with advanced caries in permanent dentition at baseline.

**Comment:** Regarding table 3, if the authors are saying 2^nd^ follow-up, then that implies they have baseline, first follow-up, and second follow-up. While I see that they included in their response to reviewer 1 that their resident statistician nixed the mixed model, you can in fact incorporate a GEE analysis for incidence or similar method to use all available data. This reviewer agrees with Reviewer 1, if you have longitudinal data, use it!

**Response**: We meant the “2-year follow-up” in table 3. To account for the repeated measures nature of the data we have adjusted all regression models for caries experience at baseline. This has the effect on including both measurements of the outcome under investigation in the same model.

**Comment**: In the discussion, I would argue that the design of the study does not allow you to prove anything, only conclude associations.

**Response:** We have reworded the Discussion to describe the findings found as associations only.
