# Supplementary material for: Carious lesions in permanent dentitions are reduced in remote Indigenous Australian children taking part in a non-randomised preventive trial
Source: PLoS One. 2021 Jan 28;16(1):e0244927. doi: 10.1371/journal.pone.0244927 (PMC7842954; doi:10.1371/journal.pone.0244927)
Supplement: Attachment — Submitted filename: Response to Reviewers_14.12.20.docx [file pone.0244927.s012.docx]

### PONE-D-19-20492R2

**Effect of a non-randomised caries preventive trial in remote Indigenous Australian children.**

Yann Benetrau, Senior Editor, PLOS ONE

Dear Yann;

We have edited the manuscript as per the recommendations of yourself and reviewer 2. We have replaced ‘impact’ with effect or effectiveness where appropriate, including in the title.

We have revised paragraph 1 of the Discussion.

Editor comments:

In particular, please note the concern raised by reviewer #2 in the review below, asking you to revise the language in the first paragraph of the discussion section. Namely, reviewer #2 asks that you do not make a claim of proving a hypothesis, but rather, of having evidence supportive of your hypothesis.

Relatedly, as reviewer #2 pointed out in an earlier version of this work, "impact" may be interpreted causally, which would not be appropriate given the study design and when reporting an association only. "Impact" also appears in your title and for the same reason can be misleading, as pointed out in this earlier review. I do note that you revised your manuscript to address this earlier comment, but claims of proof and impact remain in this latest version of your manuscript.

Reviewer 2 comment:

I think I'd just suggest the authors revise their language in Paragraph 1 of the discussion about "proving" their hypothesis. That hypothesis seems to be the standard alternative hypothesis. While I suppose one could argue the language isn't technically wrong, it is certainly...unusual. Proving something is difficult even in the best of circumstances, to say nothing of a nonrandomized single study. Your evidence is SUPPORTIVE of the hypothesis.

We hope this is acceptable.

Best wishes and regards,

**A/ Prof Ratilal Lalloo**

*Corresponding author*
